# Mitotic Machinery Dysregulation in Lung Cancer: Biological Roles, Therapeutic Targeting, and Combination Strategies

**DOI:** 10.3390/pharmaceutics18040402

**Published:** 2026-03-24

**Authors:** Bárbara Pinto, João P. N. Silva, Patrícia M. A. Silva, Bruno Sarmento, Juliana Carvalho-Tavares, Hassan Bousbaa

**Affiliations:** 1 UNIPRO—Oral Pathology and Rehabilitation Research Unit, University Institute of Health Sciences (IUCS), Cooperativa de Ensino Superior Politécnico e Universitário (CESPU), Rua Central de Gandra 1317, 4585-116 Gandra, Portugal; joaosilva_06@hotmail.com (J.P.N.S.); patricia.silva@cespu.pt (P.M.A.S.); 2 Department of Physiology and Biophysics, Institute of Biological Sciences, Federal University of Minas Gerais (UFMG), Av. Pres. Antônio Carlos 6627, Belo Horizonte 31270-901, Brazil; julianact2015@gmail.com; 3 Associate Laboratory i4HB—Institute for Health and Bioeconomy, University Institute of Health Sciences—CESPU, Rua Central de Gandra 1317, 4585-116 Gandra, Portugal; 4 UCIBIO—Applied Molecular Biosciences Unit, Translational Toxicology Research Laboratory, University Institute of Health Sciences (1H-TOXRUN, IUCS-CESPU), Rua Central de Gandra 1317, 4585-116 Gandra, Portugal; 5 i3S-Institute for Research and Innovation in Health, University of Porto, Rua Alfredo Allen 208, 4200-135 Porto, Portugal; bruno.sarmento@iics.cespu.pt; 6 INEB-Institute of Biomedical Engineering, University of Porto, Rua Alfredo Allen 208, 4200-393 Porto, Portugal

**Keywords:** lung cancer, mitotic regulators, kinase inhibitors, kinesin inhibitors, therapeutic resistance, combination therapy

## Abstract

Lung cancer remains the leading cause of cancer-related mortality worldwide and is characterized by high aggressiveness and therapeutic resistance, partly driven by mitotic dysregulation. Key mitotic regulators, including kinases such as PLK1, AURKA, AURKB, and MPS1 and kinesins such as CENPE and Eg5, are frequently overexpressed in NSCLC and SCLC, contributing to chromosomal instability, aneuploidy, and highly proliferative tumor phenotypes. Although multiple inhibitors targeting these proteins have been developed, their clinical efficacy as monotherapies has been limited. This is largely due to insufficient target dependency, adaptive resistance mechanisms, mitotic slippage, activation of compensatory pathways, and dose-limiting toxicity. This review integrates current knowledge on the physiological roles of major mitotic regulators, their dysregulation in lung tumorigenesis, and the biological and pharmacological barriers that underlie the limited success of antimitotic drugs. We further highlight preclinical and clinical evidence supporting rational combination strategies designed to enhance the antitumor activity of mitotic inhibitors while minimizing toxicity. Together, these insights underscore the need for refined therapeutic approaches that better exploit vulnerabilities in mitotic control to improve outcomes for patients with lung cancer.

## 1. Introduction

Lung cancer remains the leading cause of cancer-related mortality globally, accounting for approximately 2.5 million new cases and 1.8 million deaths in 2022. According to GLOBOCAN, one in eight cancers diagnosed globally and one in five cancer deaths are attributable to lung cancer [1]. Broadly, lung cancer is classified into two major histological categories: non-small cell lung cancer (NSCLC) and small cell lung cancer (SCLC). These subtypes differ markedly in biological features, clinical presentation, and therapeutic management [2].

NSCLC accounts for approximately 85% of all lung cancer cases, encompassing adenocarcinoma, squamous cell carcinoma, and large cell carcinoma. It is typically characterized by slower growth, frequent occurrence of actionable oncogenic drivers, and improved outcomes when diagnosed at an early stage. Despite significant advances in molecular diagnostics and targeted therapies, particularly those directed at Epidermal Growth Factor Receptor (EGFR), Anaplastic Lymphoma Kinase (ALK), and Kirsten Rat Sarcoma Viral Oncogene Homolog (KRAS) mutations, many patients are still diagnosed at advanced stages. This is largely due to nonspecific or insidious symptoms, which delays clinical detection and treatment initiation. As a result, prognosis remains poor, particularly in metastatic disease, where the median 5-year survival rate is below 5% [3,4]. In contrast, SCLC represents about 15% of all cases and is distinguished by its aggressive proliferation, early dissemination, and a strong association with tobacco exposure. Additional etiologic risk factors include radon radiation, air pollution, and advanced age [5,6]. Although SCLC initially responds to platinum-based chemotherapy and radiotherapy, relapse is nearly universal, and the overall 5-year survival rate rarely exceeds 5% [5,7]. Recent incorporation of immune checkpoint inhibitors has modestly improved outcomes, yet therapeutic resistance continues to limit long-term benefit [8]. Despite significant progress in early detection and targeted treatment of NSCLC, as well as immunotherapy advances in SCLC, both entities share a grim prognosis in their advanced stages. Tumor heterogeneity and rapid acquisition of drug resistance remain central obstacles, emphasizing the need for alternative strategies that target fundamental cellular processes involved in oncogenesis and tumor adaptive survival mechanisms [9].

The mitotic machinery, a tightly regulated network that orchestrates faithful chromosome segregation and maintains genomic integrity, has gained prominence as a critical axis in cancer biology and therapeutic innovation. Aberrant regulation of mitotic proteins has been strongly implicated in chromosomal instability and increased tumor aggressiveness. In lung cancer, key mitotic regulators such as Polo-like kinase 1 (PLK1), Aurora kinase A (AURKA) and B (AURKB), Monopolar spindle 1 kinase (MPS1), also known as Thr/Tyr kinase (TTK), and kinesin family members including Eg5, also known as Kinesin Family Member (KIF) 11 or Kinesin Spindle Protein (KSP) and Centromere-associated protein E (CENP-E), also known as KIF10 are frequently overexpressed, correlating with poor clinical outcomes and resistance to conventional therapies. Evidence from lung tumor tissue analyses supports this association. AURKB overexpression has been detected in 89% of primary NSCLC tumors compared with matched normal tissues and is associated with shorter progression-free survival (PFS) [10], while PLK1 protein levels are increased in 54.5% of lung squamous cell carcinoma tissues [11]. Transcriptomic analyses further revealed significant upregulation of mitotic genes such as AURKA, AURKB, TTK and Eg5 in NSCLC tissues compared with normal lung tissue, and AURKA and Eg5 overexpression has been reported in more than 96% of lung tumors [12,13,14]. In addition, CENP-E is significantly overexpressed in NSCLC tissues compared with normal controls, and its elevated expression correlates with poorer overall survival (OS) in lung cancer patients [15,16]. Importantly, increased expression of mitotic regulators has been associated with disease progression and worse patient prognosis, as higher AURKA and AURKB expression correlates with reduced OS and more advanced tumor stages in lung adenocarcinoma [17], while elevated PLK1 expression is significantly associated with advanced T, N and M stages and poorer OS in lung cancer patients [18]. Mechanistically, overexpression of mitotic regulators disrupts spindle assembly and centrosome homeostasis, promoting chromosomal instability and aneuploidy, which contribute to tumor initiation and progression [19]. Given their essential roles in sustaining uncontrolled proliferation, these mitotic components represent compelling targets for the development of innovative anticancer interventions [20].

Although multiple mitotic inhibitors have advanced into preclinical studies and clinical trials in lung cancer, their application as monotherapy has frequently been constrained by narrow therapeutic indices, dose-limiting toxicities such as hematologic suppression and neurotoxicity, and generally modest response rates [21]. Recent evidence suggests that combining mitotic inhibitors with chemotherapy, immunotherapy, radiotherapy and target therapy, may elicit synergistic antitumor effects while mitigating resistance pathways. This review provides a comprehensive and updated synthesis of the therapeutic landscape of lung cancer while delineating the biological foundations of mitosis and the aberrant activation of the mitotic machinery in tumorigenesis. We detail the functional roles of key mitotic regulators, such as PLK1, AURKA, AURKB, MPS1, CENP-E, and Eg5, in both normal cell division and lung cancer progression. Building on this mechanistic framework, we critically examine why mitotic inhibitors have underperformed as monotherapies in clinical settings and explore rational combination strategies that integrate these agents with current and emerging treatments. Finally, we highlight resistance mechanisms and future opportunities for exploiting mitotic vulnerabilities to achieve more durable therapeutic responses in lung cancer.

## 2. Current Therapeutic Landscape in Lung Cancer

**Figure 1:**
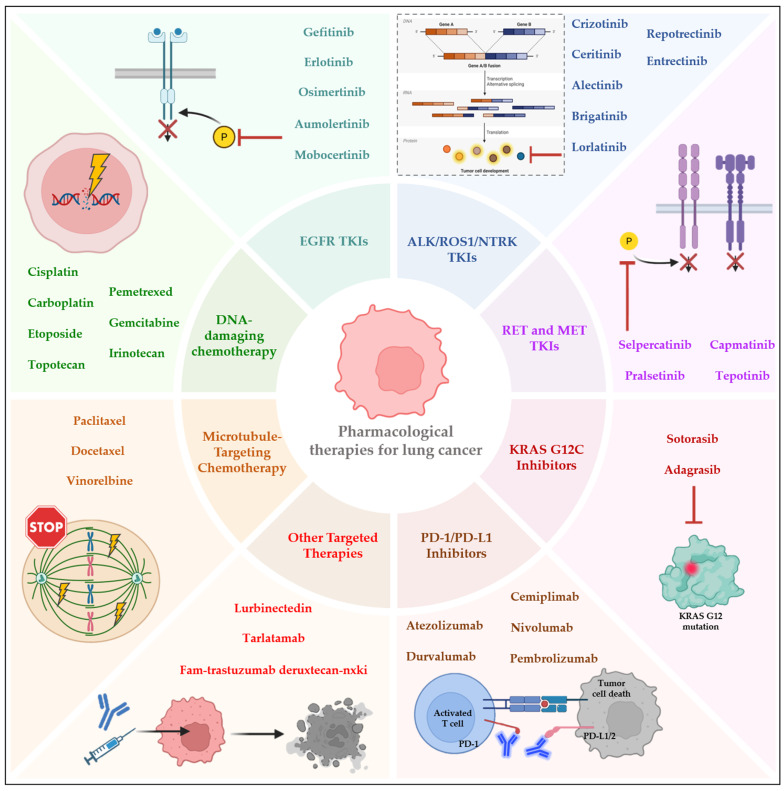
Systemic therapies for lung cancer and their main mechanisms of action. DNA-damaging chemotherapy includes agents that directly interfere with DNA structure or replication, inducing genomic damage and cell death. Platinum compounds (cisplatin and carboplatin) form DNA crosslinks, while antimetabolites (pemetrexed and gemcitabine) impair DNA synthesis. Topoisomerase inhibitors (etoposide, topotecan, and irinotecan) disrupt DNA unwinding and repair, leading to replication stress and apoptosis. Microtubule-targeting chemotherapy acts on the mitotic spindle, preventing proper chromosome segregation and inducing mitotic arrest. Taxanes (paclitaxel and docetaxel) stabilize microtubules, whereas vinca alkaloids (vinorelbine) inhibit microtubule polymerization, both ultimately blocking cell division. (EGFR TKIs selectively inhibit the intracellular kinase domain of the epidermal growth factor receptor, blocking downstream signaling pathways that promote tumor cell proliferation and survival in EGFR-mutant lung cancers. ALK/ROS1/NTRK TKIs target constitutively active fusion proteins generated by chromosomal rearrangements; these small-molecule inhibitors block the kinase activity of the aberrant fusion proteins, thereby suppressing oncogenic signaling without affecting protein expression. MET and RET TKIs target aberrantly activated receptor tyrosine kinases located at the cell membrane; capmatinib and tepotinib inhibit dysregulated MET signaling, while selpercatinib and pralsetinib selectively inhibit RET-driven oncogenic pathways, preventing downstream signal transduction that promotes tumor growth and survival. KRAS G12C inhibitors directly target the mutant KRAS protein in the cytoplasm; sotorasib and adagrasib covalently bind to the KRAS G12C mutant, locking it in an inactive state and thereby suppressing downstream proliferative signaling. PD-1/PD-L1 inhibitors are immune checkpoint inhibitors that restore antitumor immune responses by blocking the interaction between PD-1 on T cells and PD-L1 on tumor cells, releasing immune inhibition and promoting T-cell-mediated tumor cell killing. Other targeted therapies comprise agents with distinct or multimodal mechanisms of action, including antibody–drug conjugates such as fam-trastuzumab deruxtecan-nxki, which deliver cytotoxic payloads directly to tumor cells, bispecific antibodies such as tarlatamab that redirect immune cells toward cancer cells, and lurbinectedin, a DNA-binding transcriptional inhibitor that suppresses oncogenic transcription and modulates the tumor microenvironment. Abbreviations: EGFR, epidermal growth factor receptor; ALK, anaplastic lymphoma kinase; ROS1, ROS proto-oncogene 1 receptor tyrosine kinase; NTRK, neurotrophic tyrosine receptor kinase; MET, mesenchymal–epithelial transition factor; RET, rearranged during transfection; KRAS, Kirsten rat sarcoma viral oncogene homolog; PD-1, programmed cell death protein 1; PD-L1, programmed death-ligand 1; TKI, tyrosine kinase inhibitor. Created in BioRender. Silva, P. (2026). https://BioRender.com/wf8y907 (accessed on 22 January 2026).

The therapeutic landscape of lung cancer has evolved substantially over recent years, shaped by major advances in molecular biology, immuno-oncology, and precision medicine. The optimal treatment approach is primarily determined by the histological subtype and by the disease stage, as these factors critically influence both prognosis and therapeutic decision-making. NSCLC and SCLC differ markedly in their biological behavior, molecular drivers, and response to systemic therapy, necessitating distinct and increasingly personalized management strategies [22] (Figure 1).

In NSCLC, patients with early-stage disease (stages I and II) are typically managed with surgical resection, which may involve segmentectomy, lobectomy, or pneumonectomy, depending on tumor size and location. Minimally invasive surgical techniques are preferred whenever feasible, followed by adjuvant therapy to reduce recurrence risk and improve disease-free and overall survival. The benefit of adjuvant therapy is particularly evident in tumors harboring actionable genetic alterations such as EGFR or ALK mutations [23,24]. For locally advanced or metastatic disease (stages III and IV), systemic therapy remains the cornerstone of treatment. Platinum-based doublet chemotherapy, typically cisplatin or carboplatin combined with paclitaxel, docetaxel, gemcitabine, vinorelbine, or pemetrexed, continues to play an important role but is increasingly being supplemented or replaced by more precise targeted and immune-based approaches [23]. The introduction of targeted therapies has revolutionized the treatment of advanced NSCLC. Tyrosine kinase inhibitors specific for driver mutations including EGFR, ALK, ROS Proto-Oncogene 1, Receptor Tyrosine Kinase (ROS1), B-Raf Proto-Oncogene, Serine/Threonine Kinase (BRAF), REarranged during Transfection (RET), Kirsten Rat Sarcoma Viral Oncogene Homolog (KRAS), Human Epidermal Growth Factor Receptor 2 (HER2), Neurotrophic Tyrosine Receptor Kinase (NTRK) and MET Proto-Oncogene, Receptor Tyrosine Kinase (MET), have demonstrated superior efficacy and tolerability compared with conventional chemotherapy, representing a paradigm shift toward molecularly stratified treatment [25]. Among these, third-generation EGFR inhibitors such as osimertinib are preferred due to their enhanced central nervous system penetration and activity against resistance mutations like *T790M*, leading to improved outcomes in patients with central nervous system (CNS) metastases [24]. Immunotherapy has become an integral component in the management of advanced NSCLC lacking actionable driver mutations. Immune checkpoint inhibitors (ICIs) targeting Programmed Cell Death Protein 1 (PD-1), Programmed Death-Ligand 1 (PD-L1), or Cytotoxic T-Lymphocyte-Associated Protein 4 (CTLA-4), such as pembrolizumab, nivolumab, and atezolizumab, are approved for first-line use, either as monotherapy in high PD-L1–expressing tumors or in combination with chemotherapy. These regimens have demonstrated significant improvements in PFS and OS across multiple clinical trials [26,27]. Furthermore, emerging therapeutic modalities, including bispecific antibodies, antibody–drug conjugates, and cellular therapies, are being actively explored to overcome resistance mechanisms and further enhance clinical outcomes [27].

In SCLC, therapeutic strategies are guided by disease extent, traditionally categorized as limited-stage (LD-SCLC) or extensive-stage (ES-SCLC). Patients with LD-SCLC benefit from multimodal therapy integrating systemic chemotherapy with thoracic radiotherapy. The standard first-line regimen for LD-SCLC consists of concurrent chemoradiation using cisplatin and etoposide, followed by prophylactic cranial irradiation in patients who achieve complete or partial response, aiming to prevent brain metastases [28]. Surgical resection may be considered in highly selected T1–T2, N0 patients, according to TNM staging system (tumor-node-metastasis), typically followed by adjuvant therapy. For ES-SCLC, systemic therapy remains the principal approach. The standard first-line regimen, etoposide plus a platinum agent, using either cisplatin or carboplatin, is often combined with a PD-L1 inhibitor to enhance immune-mediated tumor control [29]. Despite initial chemosensitivity, most patients experience disease relapse within months, and long-term survival remains rare. Therapeutic options for relapsed or refractory SCLC are limited, and prognosis is generally poor, with median survival rarely exceeding eight months after recurrence [30]. Novel agents are emerging for relapsed or refractory SCLC, including tarlatamab, a half-life-extended bispecific T-cell engager targeting CD3 on T cells and DLL3 on tumor cells. By activating cytotoxic T cells and inducing tumor cell lysis, tarlatamab significantly improved OS (13.6 vs. 8.3 months) and PFS compared with chemotherapy (topotecan, lurbinectedin, or amrubicin). Patients also experienced better symptom control, with reductions in dyspnea and cough [31]. The main approved drugs for NSCLC and SCLC treatment are described in Table 1.

Despite these therapeutic advances, gains in long-term survival for patients with advanced lung cancer remain modest. Continued progress in molecular subtyping, biomarker-guided therapy, and innovative immunotherapeutic strategies is expected to refine patient selection and improve outcomes. Ongoing clinical trials and translational research continue to expand the therapeutic options, moving toward a more precise and effective management of both NSCLC and SCLC.

## 3. The Mitotic Machinery: Biological Functions in Normal Mitosis and Its Role in Lung Cancer

Lung cancer is characterized by profound genomic instability, deregulated cell-cycle control, and aberrant mitotic signaling, all of which collectively contribute to tumor initiation, progression, and therapeutic resistance. Substantial evidence indicates that lung tumors frequently exploit mitotic kinases, kinesins, and checkpoint regulators to sustain uncontrolled proliferation and to tolerate chromosomal instability, an otherwise lethal condition in normal cells. Understanding how the mitotic machinery becomes rewired in lung cancer is therefore essential for identifying vulnerabilities that can be exploited therapeutically. This section outlines the fundamental mechanisms governing mitosis, details the key regulators that preserve chromosomal fidelity, and subsequently discusses how their dysregulation contributes to lung tumor biology and creates actionable dependencies for targeted intervention.

### 3.1. The Mitotic Process: Mechanisms and Key Regulators

The eukaryotic cell cycle is a precisely orchestrated sequence of events that guarantees accurate DNA replication and equal segregation of genetic material into two daughter cells. This fidelity depends on the coordinated activation of several kinases and motor proteins that drive the structural and biochemical transformations from interphase to mitosis (Table 2). While cyclin-dependent kinases (CDKs) govern cell cycle progression through oscillatory activation of cyclins, mitotic fidelity relies heavily on a specialized network of kinases and motor proteins, including AURKA, AURKB, PLK1, MPS1, Eg5, and CENP-E, that dynamically regulate centrosome maturation, spindle assembly, kinetochore–microtubule interactions, and the spindle assembly checkpoint (SAC) [65,66]. The cell cycle can be divided into two major phases: interphase and mitosis. During interphase (comprising Gap 1 (G1), synthesis (S), and Gap 2 (G2) phases), the cell grows, duplicates its DNA, and prepares the machinery required for mitosis. Mitosis proceeds through five morphologically distinct sub-stages, prophase, prometaphase, metaphase, anaphase, and telophase, followed by cytokinesis, culminating in the physical division of the cell into two genetically identical daughters [67].

As cells enter prophase, the activation of the Cyclin B/CDK1 complex triggers chromatin condensation and centrosome separation, marking the commitment to mitosis [67,68]. At this stage, Aurora A kinase localizes to centrosomes, where it phosphorylates substrates critical for centrosome maturation and microtubule nucleation, such as γ-tubulin, transforming acidic coiled-coil containing protein 3 (TACC3), and targeting Protein for Xklp2 (TPX2). These phosphorylation events facilitate the recruitment of pericentriolar material and the organization of robust microtubule asters [69]. Aurora A also phosphorylates Eg5, thereby activating its microtubule-sliding activity and promoting the separation of duplicated centrosomes. Eg5 generates outward forces between antiparallel microtubules, counterbalancing inward forces from dynein and kinesin-14 motors, a process essential for establishing bipolar spindle geometry [70,71]. Failure of Aurora A or Eg5 function results in monopolar spindle formation and mitotic arrest, underscoring their interdependence in early mitotic spindle assembly [70].

Following nuclear envelope breakdown in prometaphase, microtubules emanating from spindle poles dynamically probe the cytoplasm to capture kinetochores assembled on centromeres. The fidelity of these kinetochore–microtubule attachments is ensured by the combined action of PLK1, AURKB, and the SAC kinase MPS1 [72]. PLK1 is recruited to kinetochores through its polo-box domain, and phosphorylates a range of kinetochore components, including, budding uninhibited by benzimidazole-related 1 (BubR1), and kinetochore-null 1 (Knl1) and MPS1, contributing to SAC maintenance and proper kinetochore function [65]. While PLK1 supports the stabilization of nascent kinetochore–microtubule interactions, the actual conversion from lateral to end-on attachments are driven by the coordinated action of CENP-E, the nuclear division cycle 80 (Ndc80) complex, and the spindle and kinetochore associated (Ska) complex, under the regulatory control of AURKB. These activities ensure that microtubules properly capture and align chromosomes along the spindle equator [73,74,75]

Meanwhile, AURKB, the catalytic core of the chromosomal passenger complex (CPC), localizes to the inner centromere and exerts spatially restricted phosphorylation of outer-kinetochore substrates such as Hec1/Ndc80. This phosphorylation destabilizes erroneous attachments that lack tension, thereby promoting error correction. As sister kinetochores achieve proper bi-orientation and tension is generated across the centromere, the distance from AURB increases, leading to dephosphorylation of its substrates and stabilization of correct attachments. This tension-dependent gradient of AURB activity constitutes the molecular basis for error sensing during metaphase alignment [76].

The SAC operates as a surveillance mechanism that ensures faithful chromosome segregation by delaying anaphase onset until all chromosomes achieve proper kinetochore–microtubule attachment. The process initiates at unattached kinetochores with the recruitment and activation of the serine/threonine kinase MPS1, which phosphorylates Knl1 and other scaffold proteins to recruit the checkpoint mediators budding uninhibited by benzimidazole 1 (Bub1), BubR1, mitotic arrest-deficient protein 1 (Mad1), and Mad2 [77]. Among its substrates, Bub1 plays a pivotal role, and in budding yeast, phosphorylation of Bub1 at threonine 455 by MPS1 has been shown to promote Bub1-Mad1 complex formation, a critical step in SAC activation [78].

Subsequent assembly of the mitotic checkpoint complex (MCC), composed of Mad2, BubR1, budding uninhibited by benzimidazoles 3 (Bub30), and the cell division cycle 20 (Cdc20), sequesters Cdc20 and inhibits the E3 ubiquitin ligase activity of the Anaphase-Promoting Complex/Cyclosome (APC/C). This inhibition stabilizes securin and prevents premature activation of separase, thereby maintaining metaphase arrest and safeguarding against precocious sister chromatid separation [79].

MPS1 kinase localization to kinetochores occurs through its interaction with the Ndc80 complex, and its activity is highest on kinetochores that have not yet established proper end-on attachments. MPS1 additionally regulates the recruitment of CENP-E and the Ska complex, both essential for stabilizing mature kinetochore–microtubule connections [80]. As chromosomes achieve bi-orientation and proper tension is established, AURKB activity diminishes due to spatial separation from its kinetochore substrates, while protein phosphatase 2A (PP2A)-B56 phosphatase activity promotes MPS1 dissociation, collectively driving checkpoint silencing and APC/C activation [81]. Upon APC/C-(Cdc20) activation, securin is ubiquitinated and degraded, releasing its partner separase. The concurrent decline in cyclin B-dependent CDK1 activity results in separase dephosphorylation and activation. Active separase then cleaves cohesin complexes holding sister chromatids together, marking the irreversible transition into anaphase and ensuring accurate chromosomal segregation [79,82]. Concurrently, chromosome congression at the metaphase plate is facilitated by CENP-E, that generates directional movement of laterally attached chromosomes toward the equator. Acting at kinetochores, CENP-E stabilizes end-on microtubule attachments and converts their dynamic instability into persistent directional motion, ensuring that all chromosomes achieve proper alignment before anaphase onset. As cells progress from metaphase to anaphase, CENP-E interacts with Protein Regulator of Cytokinesis 1 (PRC1) to orchestrate a critical molecular switch from lateral association to end-on capture of microtubules. This interaction promotes the remodeling of spindle microtubules into the central spindle architecture, thereby contributing to the organization and stabilization of the central spindle during anaphase initiation [83].

In addition, AURKB and PLK1 transiently remain active at the spindle midzone to coordinate chromatid segregation and initiate cytokinesis [84]. During telophase, AURKB relocalizes from centromeres to the central spindle and the midbody, where it phosphorylates substrates involved in Rat Sarcoma Viral Oncogene Homolog (Ras) homolog family member A (RhoA) activation and contractile ring assembly, thus completing cell division [85,86].

**Table 2:** Mitotic agents and biological functions.

Protein	Protein Type	Main Localization	Main Biological Functions	References
PLK1	Serine/threonine kinase (Polo-like kinase family)	Centrosomes, spindle poles, kinetochores, midbody	Coordinates G2/M transition by promoting centrosome maturation, spindle assembly, bipolar spindle formation, kinetochore assembly, and chromosome segregation. Supports proper cytokinesis and contributes to DNA-damage response through G_2_/M checkpoint control and repair pathway activation.	[87,88,89,90,91]
AURKA	Serine/threonine kinase	Centrosomes and spindle microtubules	Regulates centrosome maturation and separation, drives spindle assembly, stabilizes microtubules, and controls timely mitotic entry. Ensures formation of a bipolar spindle and accurate chromosome alignment.	[92,93,94,95]
AURKB	Serine/threonine kinase; CPC component	Centromeres, spindle midzone, midbody	Monitors and corrects kinetochore–microtubule interactions, maintains the spindle assembly checkpoint, and ensures accurate chromosome alignment. Regulates chromosomal condensation through histone phosphorylation and coordinates cytokinesis as a core component of the CPC. Contributes to the maintenance and resolution of sister chromatid cohesion.	[13,94,96,97]
MPS1	Dual-specificity kinase	Centrosomes and kinetochores	Regulates centrosome duplication, ensures accurate chromosome segregation, monitors SAC by recruiting checkpoint components to unattached kinetochores, promotes formation of the MCC, delays anaphase until proper chromosome alignment, and participates in spindle pole assembly and cytokinesis.	[98,99,100,101]
CENP-E	Kinesin-like motor protein	Kinetochores	Ensures proper chromosome congression, stabilizes kinetochore–microtubule attachments, regulates the spindle assembly checkpoint by activating BubR1 and silencing the mitotic checkpoint upon proper attachment, and promotes accurate chromosome alignment and segregation during mitosis.	[15,16,102,103,104,105,106]
Eg5	Kinesin-5 motor protein	Spindle microtubules and spindle poles	Drives centrosome and spindle pole separation, facilitates bipolar spindle assembly, supports chromosome alignment and segregation, regulates spindle dynamics, and promotes mitotic progression.	[107,108,109]

AURKA: Aurora kinase A; AURKB: Aurora kinase B; CENP-E: centromere-associated protein E; CPC: chromosomal passenger complex; Eg5: kinesin family member 11 (KIF11); MCC: mitotic checkpoint complex; MPS1: monopolar spindle 1 kinase; PLK1: polo-like kinase 1.

### 3.2. Mitotic Machinery in Lung Cancer

Dysregulation of the mitotic machinery is a hallmark cancer, including lung cancer, contributing to tumor progression, cellular transformation, and malignant phenotypes. In this section, we focus on the individual roles of key mitotic regulators, including PLK1, AURKA, AURKB, MPS1, CENP-E, and Eg5, highlighting how their overactivity or aberrant expression drives oncogenic processes. Each subsection will examine the specific contributions of these proteins to tumor proliferation, metabolic reprogramming, metastasis, and modulation of the tumor microenvironment, providing a mechanistic understanding of their role in lung cancer pathogenesis

#### 3.2.1. Role of PLK1 in Lung Cancer

PLK1 is one of the most extensively investigated mitotic regulators in lung cancer and is frequently overexpressed in NSCLC. Elevated PLK1 expression is consistently associated with advanced clinical stage, a higher proliferative index, and reduced OS [87,110,111,112,113]. Although PLK1 is fundamentally required for mitotic progression, accumulating evidence demonstrates that its overexpression profoundly contributes to the malignant phenotype of lung cancer cells through oncogenic signaling, metabolic reprogramming, promotion of metastasis, and modulation of the tumor microenvironment [114,115,116].

A major functional consequence of PLK1 overactivity is its capacity to promote tumor progression and metastatic potential. Constitutively active PLK1 enhances transforming growth factor-β (TGF-β)-dependent invasiveness by increasing the phosphorylation of Smad2 and upregulating key TGF-β–responsive genes, including Snail Family Transcriptional Repressor 1 (SNAI1), Snail Family Transcriptional Repressor 2 (SNAI2), Zinc Finger E-Box Binding Homeobox 1 (ZEB1), Cadherin 2 (CDH2), Interleukin 11 (IL11), and Tumor Necrosis Factor Alpha-Induced Protein 6 (TNFAIP6). These transcriptional changes support epithelial–mesenchymal transition (EMT), extracellular matrix remodeling, and invasive behavior in NSCLC cells. Among these targets, TNFAIP6 becomes strongly induced by PLK1-driven TGF-β activation and plays a critical role in metastasis [114]. PLK1 also affects cytoskeletal dynamics and immune escape. It phosphorylates vimentin on residues S339, T327, and S83, enhancing metastatic tumorigenesis and promoting PD-L1 expression. Phosphorylated vimentin interacts with activated Smad2/3 and facilitates their nuclear translocation. Once in the nucleus, Smad2/3 bind to the PD-L1 promoter and stimulate its transcription, enabling tumor cells to evade cytotoxic T-cell-mediated killing [115].

Additional evidence indicates that PLK1 overexpression contributes to tumor progression by modulating Phosphoinositide 3-Kinase (PI3K) signaling. PLK1 inactivates the tumor suppressor PTEN through direct phosphorylation, leading to PI3K pathway activation, increased aerobic glycolysis, and enhanced tumorigenicity [117]. PTEN, in turn, regulates PLK1 through modulation of its dephosphorylation, and PTEN activity suppresses lung cancer proliferation by dampening PLK1 signaling and promoting autophagy [110].

A metabolic dimension of PLK1 function has also emerged. PLK1 phosphorylates the pyruvate dehydrogenase E1 subunit alpha 1 (PDHA1) at Thr57, destabilizing the protein and disrupting pyruvate dehydrogenase complex integrity. This results in reduced oxidative phosphorylation, mitochondrial dysfunction, and activation of mitophagy. The ensuing mitochondrial turnover establishes a positive feedback loop that amplifies mitochondrial impairment and facilitates cancer cell proliferation [118]. Phosphorylation of PDHA1-T57 shifts cellular metabolism from oxidative phosphorylation to glycolysis, forcing cells to rely on alternative pathways, such as the aspartate-malate shuttle, to supply intermediates for the tricarboxylic acid cycle. This metabolic reprogramming supports sustained energy production and biomass accumulation, promoting lung cancer cell proliferation and survival [111].

PLK1 also promotes metastasis, immune modulation, and tumor progression through the phosphorylation of the transcription factor forkhead box M1 (FoxM1) at Ser25. Phosphorylated FoxM1 translocates to the nucleus and activates transcriptional programs that induce cytokines and chemokines via Activator Protein 1 (AP-1), nuclear factor kappa-light-chain-enhancer of activated B cells (NF-κB), and signal transducer and activator of transcription 1 (STAT1) signaling. These mediators recruit monocytes and polarize them into M2d tumor-associated macrophages, which in turn stimulate angiogenesis and metastasis through TGF-β1 and vascular endothelial growth factor A (VEGFA) secretion. Interleukin 6 (IL-6) produced under PLK1-FoxM1 signaling further reinforces this immunosuppressive microenvironment. PLK1-dependent FoxM1 activity also enhances PD-L1 expression in metastatic lesions, contributing to immune checkpoint evasion. The interferon-induced gene IFITM1 is strongly upregulated in this context and amplifies FoxM1-driven transcriptional programs associated with invasion and macrophage polarization [113].

PLK1 additionally shapes the tumor microenvironment through broader immunosuppressive effects. Tumors with high PLK1 expression exhibit diminished infiltration of cytotoxic lymphocytes and secrete elevated levels of CXCL2, which promotes M2 macrophage polarization and compromises antigen processing and presentation. High PLK1 levels correlate with reduced MHC-II expression and poorer clinical outcomes, supporting the role of PLK1 as a key modulator of the lung tumor microenvironment [119,120].

Together, these findings show that PLK1 overactivity promotes cell proliferation, suppresses apoptosis, induces chromosomal instability, and enhances metastatic and immunosuppressive programs that collectively sustain lung cancer progression [121,122]. Pharmacological inhibition of PLK1 reduces tumor cell viability and increases sensitivity to chemotherapeutic and targeted agents, further establishing PLK1 as a critical oncogenic driver and therapeutic target in lung cancer.

#### 3.2.2. Role of AURKA in Lung Cancer

In NSCLC, AURKA is frequently overexpressed, and its high expression correlates with advanced clinical stage, higher tumor grade, and reduced OS [12,92,123,124,125,126]. Although AURKA is indispensable for proper mitotic progression, accumulating evidence demonstrates that its overexpression profoundly contributes to lung cancer malignancy by promoting genomic instability, suppressing tumor-suppressive signaling, engaging multiple oncogenic pathways, and enhancing proliferation, invasion, and metastatic potential.

A major functional consequence of AURKA overactivity is its capacity to disrupt mitotic fidelity. AURKA overexpression accelerates centrosome maturation and separation, leading to centrosome amplification, aberrant spindle assembly, and the generation of aneuploid progeny [127]. This persistent chromosomal instability supports malignant transformation and provides a substrate for the accumulation of additional oncogenic alterations. Beyond these mitotic defects, AURKA directly interferes with apoptotic control. By phosphorylating TP53 at Ser215 and Ser315, AURKA impairs p53′s transcriptional and pro-apoptotic activity, thereby facilitating the survival of genomically unstable cells [123].

AURKA overexpression also engages a broad network of oncogenic signaling pathways that reinforce proliferation, motility, and tumorigenicity. AURKA regulates numerous substrates and effectors, including Protein Phosphatase 1 (PP1), PLK1, Targeting Protein for Xklp2 (TPX2), Large Tumor Suppressor Kinase 2 (LATS2), p53/p73, p27, Breast Cancer Type 1/2 Susceptibility Protein (BRCA), Ras, and components of the PI3K/Protein Kinase B (Akt)/Mechanistic Target of Rapamycin (mTOR), Mitogen-Activated Protein Kinase Kinase (MEK)/Extracellular Signal-Regulated Kinase (ERK), NF-κB, Hippo, Wnt/β-catenin, and p38 mitogen-activated protein kinase (MAPK) pathways, integrating these signals to promote tumor growth and survival [93,128]. One illustrative mechanism involves the stabilization of YAP, achieved through AURKA-mediated inhibition of autophagy, which enhances transcriptional programs associated with stem-like behavior and invasiveness [129].

Metabolic deregulation further reinforces the oncogenic role of AURKA in lung cancer. In NSCLC, AURKA phosphorylates the tumor suppressor Liver Kinase B1 (LKB1), impairing its ability to activate AMPK and thereby disrupting cellular energy homeostasis. Loss of AMPK signaling removes a key metabolic checkpoint, enabling uncontrolled proliferation and promoting increased migration and invasion [124].

Additional oncogenic circuits contribute to AURKA-driven tumorigenesis. The transcription factor ISL1, aberrantly upregulated in multiple cancers, promotes tumor formation through PI3K/Akt activation and exerts its oncogenic effects in an AURKA-dependent manner [130]. Moreover, AURKA phosphorylates Potassium Channel Tetramerization Domain Containing 12 (KCTD12), a novel protein involved in cell cycle regulation, initiating a positive feedback loop that enhances CDK1 and AURKA activity to facilitate G2/M transition, thereby stimulating cell cycle progression and tumorigenicity [131]. AURKA also cooperates with oncogenic KRAS, functioning as a key downstream effector required to sustain KRAS-driven malignant transformation. KRAS-mutant lung cancer cells rely on AURKA activity to maintain proliferation and tumorigenicity, indicating that AURKA is essential for the oncogenic program of KRAS-mutant NSCLC [132].

Collectively, these findings establish AURKA as a multifaceted oncogenic driver in NSCLC, promoting proliferation, genomic instability, metastatic competence, and microenvironmental adaptation. Its critical role in these processes underscores its relevance as a therapeutic target in lung cancer.

#### 3.2.3. Role of AURKB in Lung Cancer

In lung cancer, AURKB is frequently overexpressed, and increased AURKB levels or elevated histone H3 phosphorylation (pH3), a direct readout of AURKB activity, correlate with aggressive clinicopathological features and poor patient outcomes [10,13,133,134]. Overexpression is observed in both NSCLC and SCLC cell lines highlighting a consistent association between AURKB deregulation and lung tumorigenesis.

AURKB overactivity plays a central role in mitotic dysregulation and oncogenic progression. Excessive AURKB signaling promotes chromosomal mis-segregation, and polyploidy, generating daughter cells with abnormal DNA content. Depending on the status of key tumor suppressors such as TP53 and Retinoblastoma 1 (RB1), these mitotic abnormalities may culminate in senescence, mitotic catastrophe, or the survival of aneuploid progeny capable of driving malignant progression [133,135]. AURKB inhibition reduces pH3, induces G1/S arrest, and leads to polyploidy; cells with functional DNA-damage responses undergo senescence, whereas those without these safeguards progress to cell death [133]. In NSCLC models, increased pH3/AURKB signaling is a recurrent feature of cells that acquire tolerance to targeted therapies, particularly EGFR tyrosine kinase inhibitors [135]. Clinically, pH3 levels rise after progression on EGFR TKIs, and high baseline pH3 correlates with shorter survival [97,133].

AURKB contributes to transcriptional programs relevant to lung cancer progression, including the oncogenic activation of Cell Division Cycle Associated 8 (CDCA8), which supports tumor growth [136]. Across NSCLC cohorts, AURKB overexpression represents the predominant alteration and shows association with higher pathological stage and poor prognosis [10,13]. Hyperactivation of AURKB is also associated with vascular invasion, poor differentiation, larger tumor size, lymph node metastasis, and aneuploidy [137]. Additional evidence suggests a role for AURKB in the development and progression of lymph node metastasis in NSCLC [96].

AURKB also enhances lung cancer cell migration and invasion, with higher mRNA levels observed in tumor tissues compared with normal lung and a correlation with advanced lung adenocarcinoma (LUAD) stage. Its expression is associated with lower overall and progression-free survival, modulates immune cell infiltration, including Th2, natural killer (NK) CD56, and myeloid-derived suppressor cells, and negatively correlates with fibroblasts and endothelial cells, collectively contributing to tumor progression and malignancy [17]. AURKB overexpression further perturbs cell proliferation and the p53 pathway, regulating chromosome alignment and SAC function, and promoting aneuploidy in lung cancer cells [133].

AURKB overexpression drives mitotic dysregulation and aggressive phenotypes, but its effects depend on TP53/RB1 status, limiting the predictability of therapeutic targeting. Also, its upregulation after EGFR-TKI resistance suggests a role in adaptive tumor response.

#### 3.2.4. Role of MPS1 in Lung Cancer

MPS1 is consistently upregulated in lung cancer, with significantly higher mRNA and protein levels observed in both adenocarcinoma and squamous cell carcinoma. In LUAD, elevated MPS1 expression correlates with shorter OS [138,139].

Functionally, MPS1 overexpression promotes tumor growth, metastatic potential, and phenotypes that support lung cancer progression. One key mechanism involves upregulation of neurotensin (NTS), a neuropeptide frequently overexpressed in aggressive epithelial tumors. MPS1-driven enhancement of NTS expression increases cyclin A and CDK2 levels, thereby accelerating DNA synthesis and supporting cancer cell proliferation [139]. NTS is known to stimulate proliferation through PKC-dependent transactivation of EGFR and downstream RAF–MEK–ERK signaling, which further underscores the relevance of this axis in MPS1-mediated tumor promotion [99].

MPS1 also contributes to metastatic progression by inducing EMT. This effect is mediated by increased expression of DPYSL3, a cytosolic protein implicated in cell motility and cytoskeletal remodeling. MPS1-dependent DPYSL3 upregulation activates Snail-associated EMT programs, increasing migration and invasive behavior in vitro and in vivo; clinically, elevated DPYSL3 parallels MPS1 overexpression and correlates with poor outcome in lung cancer patients [99,139].

Upstream regulation of MPS1 stability further contributes to its oncogenic activity. MPS1 has been identified as a substrate of the X-linked deubiquitinase USP9X, which stabilizes the kinase by removing K48-linked ubiquitin chains. In NSCLC samples, USP9X and MPS1 are both upregulated and positively correlated, and loss of either protein reduces proliferation, migration, and tumorigenesis in experimental models [138]. These findings position the USP9X–MPS1 axis as an additional mechanism reinforcing mitotic checkpoint deregulation.

At a broader pathway level, bioinformatic analyses place MPS1 within a coordinated network of cell-cycle regulators, including BUBR1, BUB1, CDC45, CDC6, CHEK1, CCNB1, and CCNB2, that are co-upregulated in NSCLC. These genes collectively promote SAC control, DNA replication initiation, damage surveillance, and G2/M progression, indicating that MPS1 participates in a larger proliferative program characteristic of lung tumor cells. MPS1 expression is also enriched in T cells, NK cells, monocytes, macrophages, and dendritic cells, suggesting potential roles in shaping the immune landscape of NSCLC [101].

Together, the recurrent overexpression of MPS1, its association with advanced clinicopathologic features, and its involvement in proliferative, metastatic, and immunomodulatory processes underscore its importance in lung cancer biology and support its potential as a prognostic biomarker and therapeutic target.

#### 3.2.5. Role of CENP-E in Lung Cancer

CENP-E expression is markedly increased in lung adenocarcinoma and squamous cell carcinoma, and its increased expression is associated with poor prognosis in NSCLC, where higher CENP-E levels correlate with reduced OS [15,16,104,140]. This unfavorable outcome is more pronounced in lung adenocarcinoma, in which CENP-E expression shows a stronger association with disease progression [15,16]. Despite these findings, only a limited number of studies have investigated the contribution of CENP-E to lung cancer biology.

CENP-E participates in proliferative signaling relevant to tumor progression. Its expression shows a strong positive correlation with FoxM1, a transcription factor that regulates genes essential for G2/M transition and mitotic progression. FoxM1 directly binds to CENP-E and enhances its transcription, contributing to lung cancer cell proliferation. Higher FoxM1 levels are observed in tumor tissues and are associated with poorer outcomes, supporting the importance of the FoxM1–CENP-E axis in NSCLC biology [106].

Additional regulatory mechanisms further reinforce the relevance of CENP-E in lung cancer. XAB2, a multifunctional protein involved in RNA processing and genome maintenance, acts upstream of CENP-E since its depletion leads to reduced CENP-E mRNA and protein levels. Loss of either XAB2 or CENPE produces similar mitotic defects, consistent with the role of XAB2 in regulating mitotic progression through CENP-E. Genetic variants in XAB2 have also been linked to altered NSCLC risk, indicating that perturbations in this pathway may influence tumor development [105,141].

Altered CENP-E expression has context-dependent effects on tumorigenesis. High CENP-E expression can disrupt normal chromosome segregation and lead to aneuploidy, which may contribute to tumor initiation and progression [15]. Interestingly, experimental evidence also suggests that severe reduction in CENP-E can inhibit tumorigenesis in the presence of additional genetic damage, indicating that the consequences of altered CENP-E expression are context-dependent [103]. These findings highlight a nuanced role for CENP-E in NSCLC, where both overexpression and depletion can differentially impact tumor development.

Together, these findings indicate that CENP-E participates in cell cycle regulation, genomic instability, and proliferative signaling in lung cancer, although research on its direct involvement remains limited.

#### 3.2.6. Role of Eg5 in Lung Cancer

Eg5 dysregulation can strongly impact proliferative capacity in cancer. This kinesin is overexpressed in lung adenocarcinoma and squamous cell carcinoma, and its upregulation has been linked to increased tumor aggressiveness [108,109,142,143,144,145].

Elevated Eg5 expression is associated with advanced tumor stage, higher pathological grade, lymph node metastasis, and significantly reduced overall and progression-free survival in NSCLC patients [108,144,145]. In LUAD specifically, high Eg5 levels consistently correlate with poor prognosis and shortened patient survival [108,144]. These findings support the role of Eg5 as an unfavorable prognostic marker in lung cancer.

Functionally, Eg5 contributes to tumor progression by promoting cell proliferation, migration, and invasion. Its inhibition impairs these processes and induces cell cycle arrest and apoptosis in LUAD models, reinforcing its importance for malignant cell survival [108,109]. In addition to its mitotic functions, Eg5 has been implicated in regulating cell migration and angiogenic processes in lung cancer [142].

Eg5 overexpression also appears to influence the tumor microenvironment. High transcript levels are associated with altered immune cell infiltration, particularly involving resting NK cells, memory CD4^+^ T cells, regulatory T cells, and monocytes, all of which show significant correlations with patient survival in LUAD [108]. These observations suggest that Eg5 may participate in shaping the tumor immune landscape, extending its role beyond cell-intrinsic mitotic regulation.

Eg5 is consistently overexpressed in NSCLC, suggesting potential roles in cellular migration and tumor–immune interactions, although its precise mechanisms in lung cancer development remain poorly understood. Its persistent overexpression underscores its potential as a prognostic marker and therapeutic target.

## 4. Clinical Failures of Mitotic Inhibitors as Monotherapy in Lung Cancer

**Figure 2:**
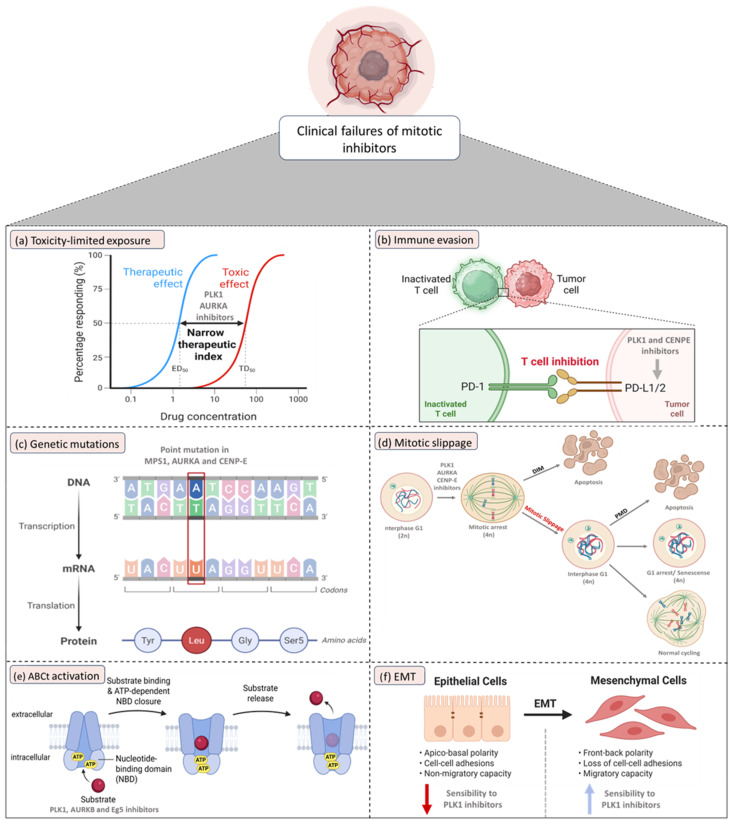
Mechanisms contributing to the clinical failure of mitotic inhibitors in lung cancer. Mitotic inhibitors targeting PLK1, AURKA, AURKB, MPS1, CENP-E, and Eg5 showed strong antitumor activity in preclinical lung cancer models but failed to achieve durable clinical responses when used as monotherapy in NSCLC and SCLC. The figure illustrates key mechanisms that undermine therapeutic efficacy, including limited drug exposure due to toxicity (**a**), immune evasion mediated by PD-1/PD-L1 signaling and its modulation by mitotic inhibition (**b**), acquisition of genetic mutations that impair inhibitor binding while preserving kinase or motor function (**c**), mitotic slippage leading to polyploidy, genomic instability, and survival of resistant cell populations (**d**), activation of ABC transporters that reduce intracellular drug accumulation and attenuate mitotic arrest (**e**), and EMT-associated phenotypic changes influencing cellular sensitivity to PLK1 inhibitors (**f**). Abbreviations: ABCt: ATP-binding cassette (ABC) transporters; DIM: Dead in mitosis; EMT: Epithelial to mesenchymal transition; PMD: Post-mitotic dead. ABCt: ATP-binding cassette transporters; DIM: Dead in mitosis; EMT: Epithelial to mesenchymal transition; PMD: Post-mitotic dead. Created in BioRender. Silva, P. (2026) https://BioRender.com/i3xu1b6 (accessed on 22 January 2026).

The targeted inhibition of key mitotic regulators, including PLK1, AURKA, AURKB, MPS1 and the kinesins CENP-E and Eg5, emerged as an attractive therapeutic approach for lung cancer due to their essential roles in mitotic progression and frequent overexpression in both NSCLC and SCLC, as well as the fact that, when inhibited, they produce catastrophic mitotic failure in cell lines and xenograft models. Nevertheless, multiple clinical trials testing these inhibitors as single agents in lung cancer malignancies showed only modest activity, transient responses, or no durable benefit (Table 3) [146,147,148,149,150,151,152]. The possible reasons for these failures are multifactorial and not yet fully defined (Figure 2).

A primary clinical limitation is the narrow therapeutic window of mitotic inhibitors. Inhibitors, mainly of PLK1 and AURKA, frequently induced dose-limiting hematologic toxicities such as neutropenia and thrombocytopenia, forcing intermittent dosing schedules that prevented sustained target inhibition required for mitotic catastrophe [147,148]. These pharmacological constraints severely reduce the therapeutic index and preclude achieving the high, continuous drug concentrations that are necessary to reproduce the mitotic arrest and apoptosis observed in preclinical models.

Following prolonged mitotic arrest, a subset of tumor cells can escape apoptosis by prematurely degrading cyclin B1, exiting mitosis without proper cytokinesis and becoming polyploid, a process known as mitotic slippage. These polyploid cells may subsequently follow three fates: (i) undergo apoptosis; (ii) enter a prolonged interphase arrest and senescense; or (iii) re-enter the cell cycle, leading to increased genomic instability and acquisition of drug resistance, ultimately contributing to tumor repopulation [153,154]. In lung cancer models, mitotic slippage has been documented following inhibition of PLK1 [155], AURKA [156] and CENP-E [20], where cells display transient mitotic arrest followed by aberrant cell cycle re-entry and polyploidization. These surviving cell populations frequently acquire enhanced proliferative or invasive potential, consistent with a more aggressive phenotype [157]. Therefore, despite the strong preclinical rationale, monotherapy with mitotic inhibitors in lung cancer frequently results in cytostatic rather than cytotoxic effects, highlighting the need for rational combination strategies to prevent adaptive resistance. Mechanistically, mitotic slippage has been extensively studied using the “competing networks-threshold model”. According to this framework, if cyclin B1 degradation occurs before the apoptotic signaling threshold is reached, the cell undergoes mitotic slippage. Conversely, if apoptotic signals are activated more rapidly, the cell initiates programmed cell death [158]. Tumor cells frequently exploit apoptosis evasion as a survival mechanism, highlighting the therapeutic potential of accelerating apoptotic signaling to prevent slippage and enhance mitotic cell death [159]. Furthermore, the unexplored synergy between mitotic inhibition and apoptosis induction in lung cancer represents a promising strategy to circumvent the limited efficacy of mitotic inhibitors as monotherapy. Combinatorial approaches targeting both apoptosis resistance and mitotic progression may provide a more comprehensive and effective strategy for eliminating lung cancer cells. Additionally, inhibition of DNA damage repair pathways after mitotic slippage can potentiate the effects of DNA-damaging agents, further enhancing therapeutic efficacy [160].

Acquired resistance, driven by both genetic and non-genetic mechanisms, further limits the efficacy of mitotic inhibitors as monotherapy. At the genetic level, structural and biochemical studies have demonstrated that point mutations within the catalytic domain of MPS1 alter the conformation of the ATP-binding pocket and disrupt inhibitor docking, thereby preventing drug binding without compromising enzymatic function. Consequently, MPS1 retains its kinase activity and sustains mitotic signaling despite pharmacologic blockade, ultimately conferring strong resistance to therapy [161,162,163]. Similarly, single-point mutations within the CENP-E motor domain impede the binding of GSK923295, an otherwise potent CENP-E inhibitor, thereby conferring marked drug resistance [164]. In addition, single-nucleotide polymorphisms (SNPs) at codon 57 of the AURKA gene (alleles II or IV) have been associated with reduced sensitivity to alisertib, an AURKA inhibitor, and with significantly shorter PFS compared with patients carrying the VV allele in both SCLC and NSCLC cohorts [165].

In parallel, non-genetic resistance mechanisms frequently involve the overexpression of ATP-binding cassette (ABC) transporters, which actively export a broad range of xenobiotics and therapeutic agents from tumor cells using ATP hydrolysis. The most prominent ABC transporters implicated in multidrug resistance include ABCB1 (P-glycoprotein/MDR1), ABCG2 (breast cancer resistance protein/BCRP), and ABCC1 (MRP1). While these transporters perform essential physiological functions, including the transport of ions, hormones and lipids, they also mediate the efflux of anticancer drugs, reducing intracellular drug accumulation and diminishing therapeutic efficacy [166]. In the clinical setting, ABCB1 and ABCG2 have been shown to interfere with the activity of numerous targeted therapies, including topotecan, gefitinib, and imatinib [167]. Volasertib, an ATP-competitive PLK1 inhibitor, acts as a substrate for ABCB1, competitively inhibiting its function and stimulating basal ATPase activity in a concentration-dependent manner [120]. Notably, volasertib has also been shown to partially reduce ABCB1 and ABCG2 expression, which may allow residual drug efflux and contribute to the limited efficacy observed in monotherapy. This partial suppression may limit intracellular drug accumulation, attenuating the cytotoxic efficacy of PLK1 inhibitors. Conversely, combined inhibition of PLK1 and EGFR more effectively downregulates ABC transporter expression, enhancing intracellular drug retention and overcoming resistance in NSCLC models [168]. BI 2536, another ATP-competitive PLK1 inhibitor, directly interacts with ABCB1 and ABCG2, functioning as a transported substrate. Overexpression of either transporter allows drug efflux, reducing intracellular BI 2536, impairing G2/M arrest, and limiting cytotoxic efficacy [169,170]. Similarly, barasertib, a selective AURKB inhibitor, has been identified as a substrate for both ABCB1 and ABCG2, exhibiting higher affinity for ABCG2 and lower affinity for ABCB1, while showing minimal interaction with ABCC2, also favoring pharmacologic resistance [171]. Additionally, ispinesib, an Eg5 inhibitor, was also newly identified as a ABCB1 substrate [172].

EMT is a dynamic and reversible biological process in which epithelial cells progressively lose cell–cell adhesion and apical–basal polarity while acquiring mesenchymal traits, including increased motility and invasive potential [173]. Although the association between EMT and drug resistance has been extensively reported, the specific mechanisms linking these processes remain incompletely understood and, in some instances, contradictory. In lung cancer, EMT has been shown to shift tumors from an immunologically “hot” to “cold” state, thereby increasing resistance to immunotherapy, whereas EMT reversal can restore sensitivity to several anticancer agents. In addition, therapeutic resistance is often associated with hybrid epithelial–mesenchymal phenotypes [174]. Notably, however, the relationship between EMT status and response to mitotic inhibitors appears distinct: NSCLC cells with a mesenchymal morphology are more sensitive to PLK1 inhibition than epithelial-like cells, with EMT induction leading to enhanced volasertib-induced apoptosis and overall sensitivity to treatment. Considering that only ~20% of NSCLC tumors display mesenchymal characteristics, the remaining ~80% retain an epithelial phenotype and may therefore be intrinsically less responsive to PLK1 inhibition, an observation that may help explain the limited efficacy of these agents in unselected NSCLC populations [175].

Moreover, immune escape is emerging as a possible contributor to drug resistance. Binding of PD-L1 to PD-1 suppresses T-cell activation, proliferation, and survival, thereby facilitating tumor immune evasion [176]. In A549 cells, elevated PD-L1 expression increases the proportion of regulatory T cells (Tregs), reinforcing an immunosuppressive microenvironment. Interestingly, CENP-E inhibition with the selective inhibitor GSK923295 further enhances PD-L1 expression in A549 cells, a response that may amplify immune suppression and ultimately support resistance to mitotic inhibition as monotherapy [16]. This upregulation of PD-L1 expression was also observed following PLK1 inhibition with volasertib, mediated by activation of the MAPK pathway [177].

**Table 3:** Clinical trials targeting PLK1, AURKA, AURKB, MPS1, CENPE and Eg5 as monotherapy for lung cancer treatment.

Drug/Regimen	Study Design/ Population	Efficacy Outcomes	Safety Profile	Main Findings	Reference
PLK1 inhibitor volasertib/300 mg i.v. (day 1) every 21 days	Phase II clinical trial/37 patients with advanced or metastatic NSCLC	3 patients showed PR. 7 patients showed SD. No CR were observed. The median PFS was 1.4 months.	Grade 3/4 AEs were observed in 22.2% of the patients.	Disappointing antitumor activity with no CR. Further clinical development as a single agent was discontinued.	[178]
PLK1 inhibitor BI 2536/200 mg i.v. (day 1) or 50–60 mg (days 1–3) every 21 days	Phase II clinical trial/95 patients with relapsed stage IIIB/IV NSCLC	4 patients showed PR. The median PFS was 8.3 weeks. OS was 28.7 weeks	Grade 3/4 AEs were observed in 54.7% of the patients. 2 treatment-related deaths.	Modest efficacy with manageable toxicity. Limited clinical benefit as monotherapy.	[146]
PLK1 inhibitor BI 2536/200 mg i.v. (day 1) every 21 days	Phase II clinical trial/23 patients with sensitive-relapsed SCLC	No OR was observed. All patients showed PD. The median PFS was 1.4 months.	Grade 3/4 AEs were observed in 52.2% of the patients. 5 treatment-related deaths	No efficacy was observed. The study terminated early due to lack of response.	[147]
PLK1 inhibitor Onvansertib/15 mg/m^2^ orally (days 1–14 every 21 days)	Phase II clinical trial/relapsed SCLC	-	-	Recruiting.	NCT05450965
AURKA inhibitor TAS-119/200 mg BID (4 days on/3 days off, 3 of 4 weeks)	Phase I clinical trial; 10 patients with SCLC in the expansion cohort	No CR or PR were observed. 5 patients showed SD.	The most common AEs were fatigue (35%), diarrhea (45%), and ocular symptoms (35%)	Disappointing antitumor activity with no objective responses. Despite manageable toxicity, TAS-119 showed limited clinical efficacy as monotherapy, leading to early study discontinuation.	[149]
AURKA inhibitor alisertib/50 mg orally twice daily (days 1–7, every 21 days)	Phase I/II clinical trial/48 patients with SCLC	10 patients showed PR and 16 showed SD. The median PFS was 2.1 months.	Grade 3/4 AEs were observed in 72% of the patients. 13 possible treatment-related deaths	Modest single-agent activity. Highest efficacy was observed in SCLC. No independent response confirmation. Limited by hematologic toxicity.	[148]
	Phase I/II clinical trial/*23* patients with NSCLC	1 patient showed PR and 17 showed SD. The median PFS was 3.1 months.	Grade 3/4 AEs was observed in 69% of the patients. 3 possible treatment-related deaths.		
AURKA inhibitor alisertib/50 or 60 mg orally twice daily (days 1–7, every 21 days)	Phase II clinical trial/Patients with extensive-stage SCLC	-	-	Recruiting.	NCT06095505
AURKB inhibitor AZD2811/200 mg i.v. once daily (days 1 and 4, every 28 days)	Phase I clinical trial/Relapsed SCLC	-	-	Terminated due to early detection of the purpose of the study. No published results.	NCT03366675
AURKB inhibitor chiauranib/50 mg orally once daily (every 21 days)	Phase III clinical trial/Progressed or Relapsed SCLC	-	-	Completed with no published results.	NCT04830813
AURKB inhibitor chiauranib/35–65 mg orally once daily (every 28 days)	Phase I/II clinical trial/Advanced solid malignant tumors and relapsed/refractory SCLC	-	-	Recruiting.	NCT05271292
AURKB inhibitor AZD 2811/200 mg i.v. (days 1 and 4 every 28 days)	Phase II clinical trial/Relapsed/refractory SCLC	-	-	Terminated due to early detection of the purpose of the study. No published results.	NCT03366675
AURKB inhibitor AZD 2811 in nanoparticles/200 mg i.v. (days 1 and 4 every 28 days)	Phase II clinical trial/15 patients with refractory SCLC	5 patients showed SD. No CR or PR were observed. The median PFS was 1.6 months.	The most common grade 3/4 AEs were neutropenia (60%) and neutropenic fever (40%).	Limited antitumor activity with no CR or PR; only a minority of patients achieved SD and PFS remained short. High rates of grade 3/4 hematologic toxicity further restricted clinical benefit.	[179]
MPS1 inhibitor S81694/4–135 mg/m^2^ i.v. (days 1, 8 and 15, every 28 days)	Phase I clinical trial/Advanced solid malignant tumors (including 5 patients with LC)	No OR was observed in patients with LC.	Grade 3/4 AEs were observed in 28.9% of the patients.	Limited single-agent efficacy. Treatment discontinuation mainly due to disease progression. Development shifted toward combination regimens.	[151]
CENP-E inhibitor GSK923295/10–250 mg/m^2^ i.v. once weekly every 28 days)	Phase I clinical trial/Advanced solid malignant tumors (including 6 patients with LC)	No OR was observed in patients with LC.	Any grade AEs was observed in 72% of the patients.	Limited single-agent efficacy. Lack of expected on-target toxicity. Suboptimal drug exposure. Further studies warranted only in optimized or combination settings.	[150]
Eg5 inhibitor LY2523355/2–5 mg/m^2^/day i.v. (days 1–3 every 21 days)	Phase I clinical trial/Advanced solid malignant tumors (including 4 patients with LC)	No OR was observed in patients with LC.	Grade 3/4 AEs were observed in 92% of the patients.	No clinical efficacy observed. High incidence of severe neutropenia. Limited value as single agent.	[152]
Eg5 inhibitor LY2523355/5 or 6 mg/m^2^ i.v. (days 1–3 every 21 days)	Phase II clinical trial/Solid tumors (including 29 patients with NSCLC)	29 patients showed SD. No CR or PR were observed in patients with LC. The median PFS was 1.3 months.	Serious AEs were observed in 37.8% of all patients.	No clinical efficacy observed. Short PFS indicating minimal therapeutic benefit.	NCT01059643
Eg5 inhibitor LY2523355/5–8 mg/m^2^ i.v. (days 1, 5 and 9 every 21 days)	Phase II clinical trial/64 patients with extensive-stage SCLC	1 patient showed OR. The median PFS was 5.7 months.	Serious AEs were observed in 37.5% of all patients.	Minimal clinical activity observed, with only one OR. The modest PFS indicated limited therapeutic benefit.	NCT01025284
Eg5 inhibitor 4SC-205/orally	Phase I clinical trial/Lymphomas and advanced solid tumors (including patients with NSCLC)	-	-	Completed with no published results.	NCT01065025
Eg5 inhibitor Ispinesib	Phase II clinical trial/Patients with advanced or metastatic NSCLC	-	-	Completed with no published results.	NCT00085813

AEs: adverse effects; AURKA: Aurora kinase A; AURKB: Aurora kinase B; CENP-E: Centromere-associated protein E; CR: complete response; i.v.: intravenous; LC: lung cancer; MPS1: Monopolar spindle 1; NSCLC: non-small cell lung cancer; OR: overall response; PFS: progression-free survival; PLK1: polo kinase 1; PR: partial response; SCLC: small cell lung cancer; SD: stable disease.

Given these mechanistic and clinical observations, it is now evident that monotherapy targeting mitotic kinases is insufficient for durable tumor control in lung cancer. The consistent failure of PLK1, AURKA, AURKB, MPS1, CENP-E, and Eg5 inhibitors as monotherapy in lung cancer clinical trials stems from mechanistically interconnected factors: (1) limited drug exposure due to hematologic toxicity, (2) mitotic slippage, (3) acquisition of genetic mutations, (4) activation of efflux pumps, (5) EMT-driven phenotypic resistance, and (6) immune escape. These failures have redirected current efforts toward combination regimens and biomarker-driven patient selection, which together may finally unlock the therapeutic potential of mitotic checkpoint inhibition in lung cancer.

## 5. Combination Therapies Targeting the Mitotic Machinery in Lung Cancer

**Figure 3:**
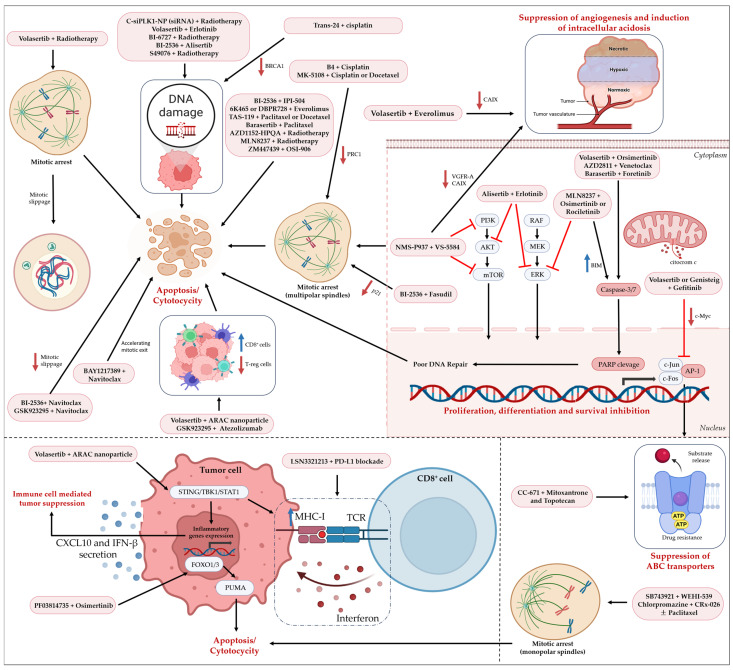
Combinatorial therapeutic strategies targeting mitotic kinases (PLK1, AURKA, AURKB, MPS1) and kinesins (CENP-E, Eg5) in lung cancer. Co-inhibition of these targets with chemotherapy, radiotherapy, tyrosine kinase inhibitors, BCL-2 family inhibitors, MEK and PI3K pathway inhibitors, immunotherapy, and tumor microenvironment modulators promotes antitumor efficacy through multiple mechanisms, including spindle and chromosome segregation disruption, DNA damage amplification, PARP-mediated repair impairment, caspase-dependent apoptosis, modulation of PI3K/AKT/mTOR and RAF/MEK/ERK signaling, AP-1 transcriptional regulation, suppression of ABC transporters, and activation of STING/TBK1/STAT1-driven inflammatory and interferon responses with enhanced MHC-I-mediated CD8^+^ T-cell recognition. These effects collectively lead to mitotic destabilization, cytotoxic cell death, reduced proliferation, altered metabolic stress, and potentiated immune-mediated tumor suppression. Abbreviations: ABC: ATP-binding cassette transporters; AKT: protein kinase B; AP-1: activator protein 1; BIM: BCL-2–interacting mediator of cell death; BRCA1: breast cancer gene 1; CAIX: carbonic anhydrase IX; c-FOS: FBJ murine osteosarcoma viral oncogene homolog; c-JUN: Jun proto-oncogene, AP-1 transcription factor subunit; C-MYC: MYC proto-oncogene, bHLH transcription factor; CXCL1: C-X-C motif chemokine ligand 1; ERK: extracellular signal-regulated kinase; FOXO1/3: forkhead box O1/O3; IFN-β: interferon beta; MEK: MAPK/ERK kinase; MHC-I: major histocompatibility complex class I; mTOR: mechanistic target of rapamycin; p21: cyclin-dependent kinase inhibitor 1A; PARP: poly(ADP-ribose) polymerase; PD-L1: programmed death-ligand 1; PI3K: phosphoinositide 3-kinase; PRC1: protein regulator of cytokinesis 1; PUMA: p53 upregulated modulator of apoptosis; RAF: rapidly accelerated fibrosarcoma kinase; STAT1: signal transducer and activator of transcription 1; STING: stimulator of interferon genes; TBK1: TANK-binding kinase 1; TCR: T-cell receptor; VEGFR-A: vascular endothelial growth factor receptor A. Created in BioRender. Silva, P. (2026) https://BioRender.com/ghm4y8w (accessed on 22 January 2026).

To overcome clinical limitations of monotherapies, combinatorial strategies targeting the mitotic machinery alongside other oncogenic pathways or therapeutic modalities have emerged as a promising approach. By simultaneously exploiting vulnerabilities in DNA damage response, cell cycle regulation, microtubule dynamics, and immune evasion, such combinations aim to enhance therapeutic efficacy, prevent resistance, and achieve more durable responses. Preclinical studies and early-phase clinical trials indicate that pairing mitotic inhibitors with standard chemotherapies, targeted therapies, or immunotherapies can potentiate tumor cell killing and mitigate adaptive resistance mechanisms (Figure 3).

In the following sections, we will provide a detailed overview of combinatorial strategies involving key mitotic regulators in lung cancer. Specifically, we will examine therapeutic combinations centered on PLK1, AURKA, AURKB, MPS1, CENP-E, and Eg5, highlighting mechanistic rationales, preclinical and clinical evidence, and translational challenges.

### 5.1. Therapeutic Combinations Involving PLK1 Inhibition in Lung Cancer

To enhance the clinical efficacy of PLK1 inhibition, multiple studies have investigated combinatorial strategies with chemotherapy, radiotherapy, targeted agents, and apoptosis modulators. These approaches aim to potentiate antitumor effects, overcome adaptive resistance, and minimize toxicity through synergistic mechanisms (Table 4).

A promising yet scarcely explored strategy involves combining PLK1 inhibition with conventional chemotherapy. In cisplatin-resistant A549/DDP cells, the small-molecule PLK1 inhibitor B4, restored cisplatin sensitivity by disrupting the PLK1/PRC1 signaling axis. In vivo the combination markedly inhibited tumor growth in both resistant (A549/DDP) and parental A549 models, achieving tumor growth inhibition rates of 74% and 53.6%, respectively. Mechanistically, the synergistic effect was linked to downregulation of PLK1 and PRC1 expression, reduced cell proliferation, and suppressed tumor growth, all without detectable systemic toxicity [116].

PLK1 inhibition has also been shown to sensitize NSCLC cells to radiotherapy. In A549 and LEPTα-2 cells, pretreatment with the selective PLK1 inhibitor BI-6727 significantly reduced clonogenic survival after irradiation comparing with radiation alone, while normal lung fibroblasts (MRC-5) remained unaffected. Mechanistically, BI-6727 treatment impaired the repair of radiation-induced DNA double-strand breaks, as evidenced by the persistent presence of γH2AX foci 24 h post-irradiation. Furthermore, the combination also markedly increased the frequency of mitotic catastrophe [180]. Similarly, volasertib, another selective PLK1 inhibitor, enhanced radiosensitivity predominantly in p53 wild-type NSCLC cells, inducing sustained G2/M arrest and cellular senescence rather than apoptosis. In contrast, p53-deficient cells showed relative resistance, emphasizing the importance of p53 status in modulating the cellular response to combined PLK1 inhibition and radiotherapy [90].

Beyond cytotoxic and radiation-based regimens, PLK1 inhibition has been explored in combination with targeted therapies, yielding promising preclinical outcomes. One such approach involves the concomitant inhibition of PLK1 and the molecular chaperone HSP90, a protein essential for the stabilization and maturation of multiple oncogenic client proteins, including kinases involved in cell cycle regulation and survival [181,182]. In H292 NSCLC cells, treatment with the PLK1 inhibitor BI-2536 markedly enhanced the cytotoxicity of the HSP90 inhibitor IPI-504, nearly doubling apoptotic cell death compared with IPI-504 monotherapy [183]. Ras homolog (Rho)/Rho-associated protein kinase (ROCK) signaling, is a classical pathway involved in regulating cytoskeletal dynamics, cell adhesion, motility, and proliferation. Aberrant activation of the Rho/ROCK pathway has been implicated in tumor progression, metastasis, and therapy resistance across several cancers, including NSCLC [184]. In KRAS-mutant lung cancer models, the combination of PLK1 inhibition (BI-2536) with ROCK inhibition (fasudil) significantly reduced cell viability, induced G2/M arrest, and triggered apoptosis more effectively than either agent alone, while sparing normal lung cells. These effects were associated with p21^WAF1/CIP1^ upregulation and nuclear localization, independent of p53 function. In vivo, the combination led to strong tumor regression and prolonged survival in KRAS-driven lung cancer mouse models, as well as marked tumor growth inhibition in patient-derived xenografts and in orthotopic A549 lung cancer models, confirming its therapeutic potential [185].

An emerging and mechanistically rational strategy involves the simultaneous inhibition of PLK1 and AURKA, two pivotal mitotic kinases that act sequentially to ensure proper spindle assembly and chromosome segregation. In SCLC, inhibition of PLK1 with BI-2536 alone induced BRCA1 and RAD51 accumulation, enhancing DNA repair and resistance. Co-treatment with alisertib suppressed this response, impairing homologous recombination, promoting G2/M arrest, and inducing mitotic death. The MYC/MYCN–RAD51 axis was identified as a key determinant of sensitivity, with MYC/MYCN-high SCLC cells showing a 4–9-fold reduction in BI-2536 IC_50_ values under dual blockade. Mechanistically, the combination triggered proteasome-mediated degradation of BRCA1 and RAD51, γH2AX accumulation, and chromatin fragmentation, culminating in mitotic catastrophe. In vivo, BI-2536 plus alisertib achieved durable tumor regression, reduced proliferation (Ki67), and increased cleaved caspase-3 and γH2AX, prolonging survival. These findings highlight synergistic DNA damage and mitotic lethality as the basis for this combination’s efficacy in MYC/MYCN-driven SCLC [186].

Further evidence indicates that PLK1 inhibition can potentiate the efficacy of EGFR-targeted therapies in NSCLC. In EGFR-mutant NSCLC cells, treatment with the PLK1 inhibitor volasertib in combination with the EGFR-TKI osimertinib significantly reduced cell viability compared with either drug alone, inducing caspase-3/7 activation, PARP cleavage, and increased Annexin V positivity. Mechanistically, PLK1 inhibition promoted EGFR degradation and enhanced apoptotic signaling, amplifying the cytotoxic effects of EGFR blockade [88]. Similarly, in erlotinib-resistant PC9 clones harboring the T790M mutation, treatment with the PLK1 inhibitor volasertib and the EGFR-TKI erlotinib synergistically decreased cell viability and induced apoptosis, whereas single agents had minimal effects. The combination increased the sub-G0 fraction and polyploidy, consistent with cell cycle disruption and apoptotic induction. Mechanistically, the co-inhibition enhanced DNA damage and the phosphorylation of γ-H2AX, p-ATR, and p-CHK1, indicated DNA stress. In vivo, this regimen significantly suppressed tumor growth in PC9-ER9 xenografts [187]. In paclitaxel-resistant NSCLC models (NCI-H460TXR and A549TXR), combining the EGFR inhibitor, gefitinib, with the PLK1 inhibitors, volasertib or genistein, produced strong synergistic cytotoxicity and reversed chemoresistance through downregulation of ABC transporters (ABCB1, ABCC9, ABCG2), mediated by suppression of the PLK1/c-MYC and EGFR/AP-1 signaling pathways. Specifically, PLK1 inhibitors decreased p-T210-PLK1 and c-Myc levels, reducing ABCB1 expression, whereas gefitinib inhibited AP-1 (c-Fos/c-Jun) activity, leading to downregulation of ABCC9 and ABCG2 [168].

Another promising approach has focused on combining PLK1 inhibition with apoptosis modulators to overcome mitotic slippage, a major limitation of antimitotic therapy. Treatment of NSCLC cells with BI-2536 alone induced mitotic arrest; however, a significant fraction of cells underwent slippage and survived. Strikingly, co-treatment with the BCL-2/BCL-xL inhibitor Navitoclax effectively reduced slippage, redirecting cell fate toward accelerated mitotic cell death. This effect was further validated in 3D spheroid models, which more accurately recapitulate in vivo tumor responses. Notably, in spheroids, the BI2536/Navitoclax combination achieved potent antitumor activity at lower doses of each drug, thereby suggesting an improved therapeutic index [155].

PLK1 inhibition has also been explored in combination with metabolic and growth-regulatory pathways. In NSCLC, dual inhibition of PLK1 and mTORC1, a key regulator of cell growth, metabolism, and survival [188], has shown strong synergistic antitumor activity. PLK1 was found to be upregulated in tumors resistant to mTORC1 inhibition, suggesting its role in adaptive resistance. Combined treatment with the mTORC1 inhibitor everolimus (RAD001) and the PLK1 inhibitor volasertib produced synergistic cytotoxicity in A549 cells and patient-derived xenograft (PDX) ex vivo cells. Also, in PDX models, this therapeutic strategy induced marked tumor regression exceeding that of monotherapies. Mechanistically, the synergy involved decreased tumor vascularization, increased HIF-1 expression, intracellular acidification, and reduced Carbonic Anhydrase 9 (CAIX), disrupting tumor pH regulation under hypoxic stress [189]. Similarly, co-inhibition of PLK1 (NMS-P937) and PI3K/mTOR signaling (VS-5584) produced synergistic antitumor effects in A549 cells, characterized by enhanced apoptosis, cell cycle arrest, and elevated reactive oxygen species (ROS) levels. In vivo, this combination significantly inhibited tumor growth without major toxicity, supporting the tolerability and therapeutic promise of this strategy. Dual inhibition was also associated with a reduction in VEGF-A, indicating impaired angiogenesis, and a decrease in CAIX, reflecting compromised pH regulation, which enhances intracellular acidosis and may contribute to tumor cell death. However, an increase in HIF-1α and GLUT1 was also observed, suggesting intensified HIF-1–driven glycolysis, a metabolic shift commonly linked to increased chemoresistance [190].

Complementary approaches have explored nanoparticle-based delivery systems (N-BDS) for PLK1-targeted therapies in lung cancer. These nanostructures offer several advantages, including targeted delivery to tumor cells, improved bioavailability, and reduced systemic toxicity [191]. For instance, a cetuximab-conjugated nanoparticle delivering PLK1 siRNA (C-siPLK1-NP) demonstrated strong synergy with radiotherapy in NSCLC models. In vitro, co-treatment in A549 and H460 cells increased γH2AX foci formation and apoptosis, indicating enhanced DNA damage. In vivo, C-siPLK1-NP combined with radiotherapy produced significant tumor regression in A549 xenografts and prolonged survival in orthotopic lung cancer models, outperforming either treatment alone. Beyond NSCLC, its potential applicability was also observed in other EGFR+ malignancies such as colorectal and breast cancers, underscoring its translational promise [192]. Another study demonstrated that PLK1 inhibition combined with PD-L1 blockade, delivered via polymer-modified mesoporous silica nanoparticles, enhanced anti-tumor efficacy. In vitro, this N-BDS reduced viability of human (A549, H460) and murine (LLC-JSP) lung cancer cells. In vivo, treatment significantly reduced tumor growth and prolonged survival compared to either monotherapy or unconjugated therapy, while allowing a reduction in effective doses by at least five-fold and exhibiting a favorable safety profile. These findings highlight the potential of nanoparticle-based co-delivery systems to enhance both therapeutic efficacy and immune modulation in lung cancer [177].

Clinical translation of PLK1 inhibition in lung cancer remains at an early stage, with limited but informative data. In a phase I open-label trial, the combination of BI-2536 with standard-dose pemetrexed was evaluated in patients with relapsed or metastatic NSCLC. Dose-limiting toxicities included grade 3 rash/pruritus and grade 4 neutropenia, establishing a maximum tolerated dose of 300 mg. Despite these toxicities, preliminary efficacy was observed, with two patients achieving partial responses and 54% maintaining stable disease after two cycles. Pharmacokinetic analyses indicated no significant drug–drug interaction, supporting the feasibility of the combination [193]. However, in a subsequent randomized phase II study in patients with advanced NSCLC progressing after platinum-based chemotherapy, volasertib combined with pemetrexed failed to improve PFS (3.3 months versus 5.3 months for pemetrexed alone), despite a higher objective response rate (21.3% versus 10.6%), reflecting limited clinical benefit and underscoring the complexity of translating preclinical synergy into durable clinical outcomes [178].

Collectively, PLK1 inhibition can synergize with chemotherapy, radiotherapy, targeted therapies, and apoptosis modulators, consistently improving preclinical efficacy across diverse NSCLC models. Despite encouraging preclinical results, clinical translation remains challenging, emphasizing the need for biomarker-guided patient selection, optimization of dosing schedules, and rational combinatorial strategies to fully exploit the therapeutic potential of PLK1 inhibition in NSCLC and SCLC.

**Table 4:** Preclinical Combination Strategies Targeting PLK1 in Lung Cancer Models.

Combination Strategy	Assay Type	Cancer Model	Mechanistic Insight/ Proposed Synergy	Main Reported Outcome	Reference
PLK1 inhibitor (B4) + cisplatin	In vitro and in vivo	NSCLC cell lines (A549/DDP, A549) and xenografts model	B4 restores cisplatin sensitivity via PLK1/PRC1 axis downregulation, leading to mitotic arrest and mitotic catastrophe. The combination exhibits synergistic cytotoxicity.	Reduced cell viability and proliferation in vitro. Inhibited tumor growth in vivo.	[116]
PLK1 inhibitor (BI-6727) + radiotherapy	In vitro	NSCLC cell NSCLC cells (A549, LEPTα-2); normal fibroblasts (MRC-5)	Impairs repair of radiation-induced DNA double-strand breaks; increases mitotic catastrophe	Reduced clonogenic survival specifically in tumor cells.	[180]
PLK1 inhibitor (volasertib 7) + radiotherapy	In vitro	NSCLC cells (p53 WT (A549, A549-NTC) and p53 knockdown/mutant (A549-920, NCI-H1975))	Induces G2/M arrest and cellular senescence.	Enhanced radiosensitivity in p53 WT (radiosensitization depends on p53 status).	[90]
PLK1 inhibitor (BI-2536) + HSP90 inhibitor (IPI-504)	In vitro	NSCLC cell line (H292)	-	PLK1 inhibition enhances HSP90-targeted apoptosis.	[183]
PLK1 inhibitor (BI-2536) + ROCK inhibitor (fasudil)	In vitro and in vivo	KRAS-mutant NSCLC cells; PDX; orthotopic models	Induces G2/M arrest and apoptosis. Upregulates p21. Exhibits tumor-specific synergy.	Reduced viability in vitro. Exhibited strong tumor regression and prolonged survival in vivo.	[185]
PLK1 inhibitor (BI-2536) + AURKA inhibitor (alisertib)	In vitro and in vivo	SCLC cell lines (NCI-H526, NCI-H82, NCI-H446, SHP77, and DMS273) and xenograft models	Dual inhibition impaired homologous recombination by reducing BRCA1 and RAD51 accumulation, promoting γH2AX foci formation, chromatin fragmentation, and mitotic catastrophe. Mechanistically governed by the MYC/MYCN–RAD51 axis.	Exhibited synergistic induction of DNA damage, apoptosis, and mitotic stress in vitro. Produced durable tumor regression and prolonged survival in vivo.	[186]
PLK1 inhibitor (volasertib) + EGFR inhibitor (orsimertinib)	In vitro	EGFR-mutant NSCLC (PC9 cells)	PLK1 inhibition promotes EGFR degradation enhancing pro-apoptotic activity of osimertinib (increases caspase-3/7 activation and PARP cleavage)	Exhibited stronger apoptotic response.	[88]
PLK1 inhibitor (volasertib) + EGFR inhibitor (erlotinib)	In vitro and in vivo	Erlotinib-resistant PC9-ER9 cells; xenograft model	Enhances DNA damage (γH2AX, p-ATR, p-CHK1), induces apoptosis, polyploidy, and causes DNA stress	Exhibited synergistic reduction in cell viability in vitro and reduced tumor growth in vivo.	[187]
PLK1 inhibitor (volasertib or genisteig) + EGFR inhibitor (gefitinib)	In vitro	Paclitaxel-resistant NSCLC (NCI-H460TXR, A549TXR)	Suppresses PLK1/c-Myc and EGFR/AP-1 pathways; downregulates ABC transporters	Exhibited synergistic cytotoxicity activity.	[168]
PLK1 inhibitor (BI-2536) + BH3 mimetic (navitoclax)	In vitro	NSCLC cells (A549)	Reduces mitotic slippage; promotes mitotic cell death by apoptosis.	Exhibited potent antitumor activity at lower doses. Induced minimal cytotoxic effects in non-tumor cells.	[155]
PLK1 inhibitor (volasertib) + mTORC1 inhibitor (everolimus)	In vitro and in vivo	NSCLC cells (A549) and PDX models.	Reduces vascularization, increases HIF-1α, induces intracellular acidification and downregulates CAIX	Exhibited synergic antitumor activity in vitro. Reduced tumor regression in vivo.	[189]
PLK1 inhibitor (NMS-P937) + dual PI3K/mTOR inhibitor (VS-5584)	In vitro and in vivo	NSCLC cells (A549) and xenograft models	Disrupts PI3K/mTOR–mitotic crosstalk. Induces cell cycle arrest, ROS accumulation, and apoptosis. Decreased VEGFA and CAIX with increased HIF1-α and GLUT1.	Exhibited synergistic antitumor activity in vitro and significant tumor growth inhibition in vivo without notable toxicity.	[190]
C-siPLK1-NP (siRNA) + radiotherapy	In vitro and in vivo	NSCLC cells (A549, H460); xenografts.	Enhances DNA damage (γH2AX).	Exhibited synergistic cytotoxicity activity in vitro and significant tumor regression and extended survival in vivo.	[192]
PLK1 inhibitor (volasertib) + PD-L1 antibody (ARAC nanoparticle)	In vitro and in vivo	NSCLC cells (A549, H460), murine cells (LLC-JSP); orthotopic and metastatic lung cancer models.	Co-delivery enhances anti-tumor immune response while inhibiting PLK1; combination reduces tumor growth and increases CD8^+^/Treg ratio.	Reduced cell viability in vitro. Decreased tumor growth and prolonged survival in vivo. Achieved the same or greater therapeutic effect at a 5-fold lower dose compared to unconjugated therapy. Exhibited favorable safety profile.	[177]
PLK1 inhibitor (BI 2536) + pemetrexed	Phase I clinical trial	Relapsed/metastatic NSCLC patients	-	Of 39 patients analyzed, 2 showed PR and 21 showed SD. No CR was observed. The most common grade 3/4 AEs was neutropenia (24%) and febrile neutropenia (12%).	[193]
PLK1 inhibitor (volasertib) + pemetrexed	Phase II clinical trial	Advanced NSCLC patients	-	Of 47 patients analyzed, 10 showed PR and 21 showed SD. No CR was observed. The most common grade 3/4 AEs was neutropenia (10.9%) and fatigue (8.7%).	[178]

AP-1: activator protein 1; AURKA: Aurora kinase A; CAIX: carbonic anhydrase IX; CR: complete response; EGFR: epidermal growth factor receptor; GLUT1: glucose transporter 1; HIF1-α: hypoxia-inducible factor 1 alpha; HSP90: heat shock protein 90; mTOR: mechanistic target of rapamycin; NSCLC: non-small cell lung cancer; PARP: poly (ADP-ribose) polymerase; PI3K: phosphoinositide 3-kinase; p-ATR: phosphorylated ataxia telangiectasia and Rad3-related protein; p-CHK1: phosphorylated checkpoint kinase 1; PLK1: polo-like kinase 1; PR: partial response; PRC1: protein regulator of cytokinesis 1; ROS: reactive oxygen species; SD: stable disease; VEGFA: vascular endothelial growth factor A; γH2AX: phosphorylated histone H2AX.

### 5.2. AURKA Inhibition-Based Combination Therapies in Lung Cancer

Recent efforts have focused on evaluating AURKA inhibitors in combination with chemotherapies, radiotherapy, targeted therapies, and immunotherapies to improve efficacy, overcome resistance, and expand therapeutic options in both NSCLC and SCLC cell lines (Table 5).

One of the earliest preclinical studies tested the selective AURKA inhibitor MK-5108 in a panel of NSCLC cell lines, including H358, H1355, H460, A427, H1666, H1975, A549, Calu-1, HCC827, H1650 and H727. MK-5108 induced G2/M arrest, polyploidy, and apoptosis, while suppressing expression of AURKA, TACC3 (an AURKA substrates that regulates spindle poles microtubule stabilization), and PLK1. Notably, combinations of MK-5108 with cisplatin or docetaxel produced synergistic effects, with docetaxel being superior. Sequential administration also revealed that docetaxel treatment followed by MK-5108 was more effective than the reverse, although concurrent exposure yielded the greatest inhibition, underscoring the importance of treatment scheduling [156]. Consistent with these findings, TAS-119, another selective AURKA inhibitor, enhanced the anticancer activity of paclitaxel and docetaxel across several cancer models, including SHP-77, A549, A427, NCI-H460, and paclitaxel-resistant (A549.T12) lung cancer cell lines. Notably, TAS-119 displayed tumor-selective activity, as it did not enhance paclitaxel antitumor activity in normal lung fibroblast cell lines WI-38 and MRC5. TAS-119 in combination with paclitaxel or docetaxel also reduced tumor volume and was well tolerated in in vivo models [194]. Similarly, in a Phase I clinical trial study involving patients with advanced solid tumors, the combination of alisertib, with nab-paclitaxel demonstrated durable responses in selected patient subsets, including those with SCLC [195]. Parallel findings of a Phase II study evaluated the combination of alisertib with paclitaxel in relapsed or refractory SCLC. Although the regimen showed efficacy signals, particularly in patients with c-Myc expression and mutations in cell-cycle regulators (CDK6, RBL1, RBL2, RB1), the clinical benefit was offset by increased toxicity. Compared with paclitaxel alone, the combination was associated with a higher incidence of grade ≥ 3 treatment-related adverse events (67% vs. 25%), including a high rate of severe neutropenia (38%) and four treatment-related deaths due to infectious complications. Despite these limitations, patients with biomarker-defined subgroups derived significant improvements in PFS and OS, suggesting that the therapeutic potential of alisertib may rely on careful patient selection and prospective validation of predictive biomarkers [196].

AURKA inhibition has also been explored in combination with radiotherapy. In vitro studies demonstrated that treatment with the AURKA inhibitor MLN8237, together with ionizing radiation, markedly increased caspase-3 cleavage in NSCLC cell lines, besides to inhibit tumor cells proliferation. Consistently, in vivo experiments confirmed that MLN8237 (30 mg/kg for 30 days) enhanced the efficacy of fractionated radiotherapy (2 Gy daily for 5 consecutive days), resulting in increased caspase 3 cleaved, significant delay in tumor growth and, increased apoptotic activity [197]. These findings suggest that AURKA blockade can potentiate the cytotoxic effects of radiation, providing a rationale for its use as a radiosensitizer in NSCLC.

Beyond radiotherapy, dual inhibition strategies targeting AURKA and oncogenic drivers have shown promise. EGFR is a receptor tyrosine kinase frequently overexpressed or mutated in NSCLC, activating downstream signaling cascades such as the RAS/RAF/MEK/ERK and PI3K/Akt pathways, which promote proliferation and survival [198]. In KRAS-mutant NSCLC, alisertib combined with erlotinib (EGFR inhibitor) significantly reduced cell viability, clonogenic survival, and xenograft growth compared to single agents. Mechanistically, this synergy was associated with suppression of EGFR downstream signaling, including inhibition of ERK activation in A549 cells, as well as marked reduction in Akt activity in A549 but not in non-tumor H358 cells. These molecular changes coincided with accumulation of aneuploid cells and increased apoptosis. In vivo, tumor growth was effectively inhibited without notable toxicity [199]. Similarly, combining MLN8237 with third-generation EGFR-TKIs, such as osimertinib or rociletinib, enhanced tumor regression, suppressed proliferation, and induced apoptosis in EGFR-mutant NSCLC cell line and PDX models. Mechanistically, AURKA inhibition disrupted TPX2-mediated activation, enhanced BIM and cleaved PARP, suppressed ERK and NF-κB signaling, and restored apoptotic machinery, effectively sensitizing resistant cells to EGFR inhibition. In vivo, the combination decreased proliferating cells and increased cleaved caspase-3 levels. Importantly, this effect was observed in models with elevated TPX2/AURKA signaling, including residual disease and acquired resistant tumors, without apparent toxicity [200].

The integration of AURKA inhibition with immunotherapy represents a particularly promising avenue. Preclinical studies using the highly specific AURKA inhibitor LSN3321213 in immunocompetent SCLC mouse models showed that combining LSN3321213 with PD-L1 blockade significantly enhanced tumor regression, survival, and induced durable anti-tumor immunity. Mechanistically, AURKA inhibition enriched tumor cells in M phase, enhanced interferon signaling, and increased MHC-I-mediated antigen presentation, effectively “priming” tumors for more robust immune responses upon PD-L1 checkpoint blockade. Tumors from mice treated with LSN3321213 + PD-L1 showed the highest enrichment of CD4^+^ memory T cells, CD8^+^ effector T cells, CD8^+^ central memory T cells, CD8^+^ effector memory T cells, and interferon-primed CD8^+^ T cells compared to other treatment arms, with no significant changes in naive T cells. This profile is consistent with a productive anti-tumor T-cell response [201]. In SCLC, where MYC family gene alterations are frequent, preclinical data indicate strong vulnerability to AURKA inhibitors. MYC genes exert pleiotropic effects on cell-intrinsic pathways and the tumor microenvironment, regulating growth, survival, and differentiation, and influencing immune surveillance to promote oncogenic progression [202]. Case-based evidence demonstrates that AURKA inhibition can induce prolonged responses in tumors harboring MYC alterations, and sequential treatment with the PD-1 inhibitor, nivolumab, may also be effective, suggesting a potential link between MYC-driven tumor biology and sensitivity to both mitotic disruption and immune checkpoint blockade [203].

Similarly, next-generation AURKA inhibitors such as 6K465 and its prodrug DBPR728 have shown potent MYC destabilization and strong antitumor activity in MYC-driven SCLC models. In vitro, combination treatment of 6K465 with the mTOR inhibitor everolimus produced synergistic growth inhibition in NCI-H69 and NCI-H446 cells. In vivo, oral administration of DBPR728 with everolimus markedly reduced tumor volume and delayed growth of PI3K-mutant SCLC xenografts, demonstrating durable regression and a robust synergistic effect. These findings highlight the therapeutic potential of combining AURKA and mTOR inhibition in biomarker-defined subsets of SCLC [204].

Several AURKA inhibitors in combination therapeutic regimens have entered clinical testing, although tolerability has been a limiting factor. The combination of alisertib and irinotecan, a topoisomerase-I inhibitor, was evaluated in patients with advanced lung cancer and other solid tumors. In this study, one SCLC patient achieved a partial response lasting nine cycles, and two others had stable disease, whereas the remaining evaluable patients progressed. The regimen caused significant hematologic and gastrointestinal toxicities, including grade 4 neutropenia and diarrhea, which limited dose escalation. Overall, the combination showed modest efficacy at tolerable doses, with the maximum tolerated dose (MTD) below the pharmacodynamically active range of alisertib, suggesting that alternative dosing or supportive strategies are needed to optimize anti-tumor activity [205].

Other early-phase clinical trials are also investigating AURKA inhibitors in combination with targeted therapies for NSCLC. Alisertib or LY3295668 combined with osimertinib is being tested in EGFR-mutant NSCLC, with some trials actively recruiting (NCT04085315) or ongoing (NCT05017025), while others were completed or terminated without published results (NCT04479306, NCT01471964). Alisertib has also been combined with the KRAS G12C inhibitor sotorasib in KRAS-mutant NSCLC, though it was terminated due to lack of response and availability of newer therapies (NCT05374538). These trials highlight the clinical interest in targeting AURKA to overcome resistance in molecularly defined NSCLC.

Collectively, AURKA inhibition demonstrates robust anti-tumor activity in lung cancer, particularly in combination with chemotherapies, EGFR inhibitors, radiotherapy, or immune checkpoint blockade. Preclinical studies show induction of mitotic arrest, apoptosis, and genomic instability, with combination strategies enhancing efficacy through synergistic cytotoxicity, radiosensitization, or immune modulation. Mechanistic and biomarker analyses highlight the relevance of MYC amplification, TPX2/AURKA signaling, KRAS mutations, and treatment scheduling. Early clinical studies report modest efficacy with dose-limiting toxicities, emphasizing the need for optimized dosing and biomarker-guided patient selection.

**Table 5:** Preclinical Combination Strategies Targeting AURKA in Lung Cancer Models.

Combination Strategy	Assay Type	Cancer Model	Mechanistic Insight/ Proposed Synergy	Main Reported Outcome	Reference
MK-5108 (AURKA inhibitor) + cisplatin or docetaxel	In vitro	NSCLC cell lines (H460 and Calu-1).	MK-5108 induces G2/M arrest, polyploidy, and apoptosis. Combination with chemotherapy enhances 82uu	Synergistic reduction in cell viability. Docetaxel combination was more effective than cisplatin. Efficacy depends on treatment schedule.	[156]
AURKA inhibitor (TAS-119) + paclitaxel or docetaxel	In vitro and in vivo	NSCLC cell lines (A549, A427, NCI-H460), SCLC cell line (SHP-77), paclitaxel-resistant line (A549.T12), and NCI-H460 xenografts model.	TAS-119 selectively inhibits AURKA and potentiates taxane-induced cytotoxicity	Enhanced anticancer activity in both sensitive and resistant cell lines. In vivo, it was well tolerated and decreased tumor volume. Induced minimal cytotoxic effects in non-malignant cells.	[194]
AURKA inhibitor (alisertib) + nab-paclitaxel	Phase I clinical trial	Advanced solid tumor patients.	-	Of 5 patients analyzed, 1 showed PR and 2 showed SD. The most common grade 3/4 AEs were neutropenia (67.7%) and leukopenia (61.3%).	[195]
AURKA inhibitor (alisertib) + paclitaxel	Phase II clinical trial	Relapsed or refractory SCLC patients.	Combination with paclitaxel enhances cytotoxicity, especially in c-Myc–expressing or cell-cycle altered tumors.	Of 89 patients analyzed, 1 showed CR, 19 showed PR, and 49 showed SD. The most common grade 3/4 AEs were neutropenia (38%) and diarrhea (15%).	[196]
AURKA inhibitor (MLN8237) + radiotherapy	In vitro and in vivo	NSCLC cell lines (H460, HCC2429) and H460 xenograft model.	Combination inhibits caspase-3 activation and enhances apoptosis.	Exhibited significant tumor growth delay and increased apoptotic activity in vivo, demonstrating radiosensitization.	[197]
AURKA inhibitor (Alisertib) + EGFR inhibitor (erlotinib)	In vitro and in vivo	KRAS-mutant NSCLC cell lines (A549, H358), and A549 and H358 xenografts models.	Combination suppresses EGFR downstream signaling, reduces ERK and Akt activity, induces aneuploidy and apoptosis.	Reduced cell viability and clonogenic survival in vitro. Potentiated tumor growth suppression in vivo. Combination was well tolerated.	[199]
AURKA inhibitor (MLN8237) + EGFR inhibitor (osimertinib or rociletinib)	In vitro and in vivo	EGFR-mutant NSCLC cell lines (H1975, PC9-RR, PC9-OR) and patient-derived xenografts	Combination suppresses EGFR downstream signaling, disrupts TPX2-mediated AURKA activation, increases increased BIM and cleaved PARP, suppresses ERK and NF-κB activity, restores apoptotic machinery, and increases cleaved caspase-3 levels	Reduced cell viability and clonogenic survival in vitro. Potentiated tumor regression, suppressed proliferation, and induced apoptosis in vivo. Combination was well tolerated.	[200]
AURKA inhibitor (LSN3321213) + PD-L1 blockade	In vivo	Immunocompetent SCLC mouse models.	AURKA inhibition enriches tumor cells in M phase, enhances interferon signaling, and increases MHC-I-mediated antigen presentation to prime immune response.	Enhanced tumor regression, prolonged survival, increased infiltration of memory and effector T cells, and durable antitumor immunity.	[201]
AURKA inhibitor (MLN8237) + PD-1 blockade (nivolumab)	Case-based clinical evidence	SCLC patients.	-	AURKA inhibition induced prolonged responses; sequential treatment with nivolumab showed additional therapeutic benefit.	[203]
AURKA inhibitor (6K465 or DBPR728) + mTOR inhibition (everolimus)	In vitro and in vivo	SCLC cell lines (NCI-H69, NCI-H446) and PI3K-mutant SCLC xenograft models.	Disrupts proliferative signaling, leading to synergistic cytotoxicity	Exhibited synergistic suppression of cell growth in vitro and marked tumor volume reduction and delayed progression in vivo, indicating durable antitumor efficacy.	[204]
AURKA inhibitor (Alisertib) + mTOR inhibitor (irinotecan)	Phase I clinical trial	Advanced lung cancer and other solid tumors.	-	Of 17 patients analyzed, 1 showed PR and 2 showed SD. The most common grade 3/4 AEs was neutropenia (24%) and diarrhea (24%).	[205]
AURKA inhibitor (Alisertib) + EGFR inhibitor (Osimertinib)	Phase I clinical trial	EGFR-mutated stage IV NSCLC patients	-	Currently recruiting.	NCT04085315
AURKA inhibitor (LY3295668) + EGFR inhibitor (Osimertinib)	Phase I/II clinical trial	Advanced EGFR-mutant NSCLC patients	-	Active, not recruiting.	NCT05017025
AURKA inhibitor (Alisertib) + EGFR inhibitor (Osimertinib)	Phase I clinical trial	EGFR-mutated stage IIIB or IV NSCLC patients	-	Completed with no published results.	NCT04479306
AURKA inhibitor (Alisertib) + EGFR inhibitor (Erlotinib)	Phase I/II clinical trial	Recurrent or Metastatic NSCLC patients	-	Terminated with no published results.	NCT01471964
AURKA inhibitor (VIC-1911) + KRAS^G12C^ inhibitor (Sotorasib)	Phase I clinical trial	Advanced or metastatic KRAS G12C-mutated NSCLC	-	Terminated by sponsor decision, after new KRAS^G12C^ therapies became available. No patient showed CR, PR or SD.	NCT05374538

AURKA: Aurora kinase A; CR: complete response; EGFR: epidermal growth factor receptor; ERK: extracellular signal-regulated kinase; MHC-I: major histocompatibility complex class I; NF-κB: nuclear factor kappa-light-chain-enhancer of activated B cells; NSCLC: non-small cell lung cancer; PR: partial response; SD: stable disease; SCLC: small cell lung cancer; TPX2: targeting protein for Xklp2.

### 5.3. AURKB Inhibition-Based Combination Therapies in Lung Cancer

To enhance the efficacy of Aurora B inhibition, multiple studies have explored combination strategies with chemotherapy, targeted therapies, radiotherapy, and multi-kinase inhibitors. These approaches aim to potentiate antitumor activity, overcome drug resistance, and exploit tumor-specific vulnerabilities through mechanistic synergy (Table 6).

One strategy explored in NSCLC is the combination of AURKB inhibitors with taxanes. Nevertheless, in contrast to AURKA inhibition, which typically enhances taxane sensitivity, AURKB blockade elicited an antagonistic effect. Treatment with the selective AURKB inhibitor barasertib diminished paclitaxel efficacy in a dose-dependent manner. AURKB inhibition was confirmed by the marked reduction in histone H3 serine 10 (H3S10) phosphorylation, a direct AURKB substrate, while AURKB mRNA levels remained unaffected. Increasing concentrations of barasertib consistently induced resistance to paclitaxel across multiple NSCLC cell lines, underscoring Aurora B activity as a pivotal determinant of taxane responsiveness [13].

AURKB inhibition has also been evaluated in combination with targeted therapies to overcome resistance mechanisms in EGFR-mutant NSCLC. In vitro, AURKB inhibition (PF03814735) synergized with the EGFR-tyrosine kinase inhibitor (Osimertinib), by stabilizing the proapoptotic protein BIM through reduced Ser87 phosphorylation and inducing PUMA via FOXO1/3, enhancing apoptosis. Osimertinib-resistant cells generated through EMT, a well-known mechanism of drug resistance in EGFR-mutant NSCLC [206], exhibited hypersensitivity to AURKB inhibition, triggering mitotic catastrophe and cell death. In vivo, combined treatment with osimertinib and PF03814735 effectively suppressed tumor growth and limited regrowth in both parental and osimertinib-resistant xenografts, as well as in PDX models, demonstrating the potential of this combination to overcome multiple forms of drug resistance [207]. Insulin-like growth factor 1 (IGF1) is a key mitogenic and anti-apoptotic signaling molecule that exerts its effects primarily through binding to the IGF1 receptor (IGF1R), activating downstream pathways such as PI3K/Akt, JAK/STAT and MAPK/ERK. In the context of NSCLC, IGF1 signaling has been implicated in promoting tumor cell proliferation, survival, and resistance to targeted therapies [208,209]. Accordingly, dual targeting of IGF1R and AURKB has been explored to exploit mechanistic synergy. In A549 cells, combined inhibition with OSI-906, an IGF1R inhibitor, and the AURKB inhibitor ZM447439 led to a pronounced reduction in cell viability. While 3 µM OSI-906 alone exerted minimal cytotoxicity, its combination with 0.6 or 1 µM ZM447439 significantly decreased viability, with combination index (CI) values of 0.32 and 0.35, respectively, confirming strong synergism [210].

AURKB inhibition has also been investigated as a radiosensitization strategy in NSCLC models. The selective AURKB inhibitor AZD1152-HPQA displayed a cell line-dependent effect when combined with ionizing radiation. In clonogenic survival assays, AZD1152-HPQA significantly enhanced radiosensitivity in H460 cells, while showing minimal effect in A549, H520, and H661 cells. Mechanistically, this radiosensitization was not associated with increased apoptosis but rather with suppression of tumor cell repopulation during fractionated irradiation schedules. AZD1152-HPQA treatment reduced the TCD_50_ (radiation dose required to control 50% of plaque monolayers) by up to 75% in H460 cells, primarily through inhibition of proliferative recovery between irradiation cycles. Furthermore, AURKB inhibition induced polyploidy and loss of clonogenic potential, indicating that AZD1152-HPQA enhances the efficacy of radiotherapy mainly by limiting repopulation and promoting mitotic failure rather than directly augmenting radiation-induced apoptosis [211]. Complementarily, the multi-kinase inhibitor S49076, which targets MET, AXL, and FGFR1-3, was also shown to inhibit AURKB in NSCLC cells (H441, A549, and H460). These receptor tyrosine kinases are frequently dysregulated in lung cancer and contribute to tumor cell proliferation, survival, and therapeutic resistance [212,213,214]. In vitro, the combination of S49076 with irradiation produced additive effects on clonogenic survival in H441 and A549 cells, while slightly increasing radiosensitivity in H460 cells, independent of MET dependency. Co-treatment did not alter MET expression or phosphorylation after irradiation but markedly reduced histone H3 phosphorylation, confirming AURKB inhibition. In vivo, oral administration of S49076 twice daily, combined with fractionated thoracic irradiation, significantly delayed tumor growth in orthotopic H460-luc and A549-luc models. The combination treatment further suppressed tumor progression and prolonged survival [214].

Complementing AURKB inhibition strategies, the multi-kinase inhibitor foretinib, which partially targets AURKB along with MEK1/2 and FER signaling, has been shown to synergize with the selective AURKB inhibitor barasertib in MYC-amplified SCLC cell lines. While foretinib alone moderately inhibits AURKB and induces apoptosis in sensitive NSCLC cells, its combination with barasertib significantly enhances cell death, and increases PARP1 and caspase-3 cleavage. This synergistic effect is specific to MYC-amplified cells and is not observed in non-MYC-amplified models, highlighting the potential of combining polypharmacology-based compounds with selective Aurora B inhibition to maximize therapeutic efficacy in lung cancer [215]. Recent CRISPR/Cas9 screens revealed a synthetic lethal interaction between AURKB and Haspin (GSG2) in cancer cells, showing that dual inhibition is more cytotoxic than either agent alone [216]. Haspin is a serine/threonine protein kinase that phosphorylates histone H3 at threonine 3, a key step for proper chromosome alignment during mitosis [216]. In NSCLC models, the Haspin inhibitor CHR-6494 combined with the pan-Aurora kinase inhibitor VX-680, or with barasertib, significantly reduced cell viability compared with single-agent treatments. Notably, KRAS-mutant cell lines (A549, H358, H460) were more sensitive to these combinations than non-KRAS-mutant lines (H1299, H1975), consistent with AURKB acting downstream of KRAS signaling. These findings support combined inhibition of AURKB and Haspin as a promising strategy for KRAS-driven NSCLC [217].

Similarly, in SCLC, the combination of AZD2811 with the selective BCL2 inhibitor venetoclax was evaluated to overcome intrinsic resistance. Venetoclax markedly sensitized BCL2-overexpressing SCLC cells to AZD2811, restoring apoptosis and enhancing DNA damage, whereas cells with low BCL2 expression were minimally affected. In vivo, AZD2811NP combined with venetoclax improved tumor growth inhibition in BCL2-high xenograft and PDX models compared with either agent alone, with sustained responses observed even after treatment cessation, indicating that co-targeting AURKB and BCL2 can enhance antitumor efficacy in select SCLC contexts [218].

In addition to preclinical advances, early clinical attempts to combine AURKB inhibition with immunotherapy have been conducted in SCLC. Two phase II trials evaluated AZD2811 plus the PD-L1 inhibitor durvalumab. The study in relapsed SCLC (NCT04525391) was terminated early due to serious unexpected adverse events reported in other trials using AZD2811, preventing efficacy assessment. A second trial in extensive-stage SCLC (NCT04745689) remains active but not recruiting, with no efficacy results reported to date. These efforts illustrate the translational interest in AURKB–PD-L1 co-targeting, while also emphasizing safety challenges that may limit clinical progression.

AURKB inhibition shows clear mechanistic potential across lung cancer models, particularly when combined with chemotherapy, targeted therapies, or radiotherapy. Preclinical data indicate that such combinations can overcome specific resistance mechanisms, such as EMT-mediated EGFR-TKI resistance or KRAS-driven tumor growth, and enhance apoptosis in select contexts, including MYC-amplified cells. However, responses are highly context-dependent, varying with genetic background, cell type, and treatment modality. While these findings support the rationale for combination strategies, they also highlight that efficacy is not uniform and that some cell lines or models exhibit minimal benefit. This underscores the need for further systematic evaluation to identify predictive biomarkers, optimize drug pairing, and define which patient populations are most likely to benefit from AURKB-based combination therapies.

**Table 6:** Preclinical Combination Strategies Targeting AURKB in Lung Cancer Models.

Combination Strategy	Assay Type	Cancer Model	Mechanistic Insight/ Proposed Synergy	Main Reported Outcome	Reference
AURKB inhibitor (barasertib) + paclitaxel	In vitro	NSCLC (A549, SK-MES1, SKLU1, LUDLU1, CRL5807, CRL5802, CORL23, CALU6, CALU3)	Aurora B inhibition decreased H3S10 phosphorylation and reduced paclitaxel-sensitivity of NSCLC.	Barasertib reduced paclitaxel efficacy in a dose-dependent manner; AURKB activity identified as a determinant of taxane response.	[13]
AURKB inhibitor (PF03814735) + EGFR-TKI (osimertinib)	In vitro and in vivo	EGFR-mutant NSCLC, osimertinib-resistant models (H1975R and ECLC26R)	AURKB inhibition stabilized BIM (decreases Ser87 phosphorylation) and induces PUMA via FOXO1/3, enhancing apoptosis; EMT-mediated resistance increases AURKB dependency.	Combination enhanced apoptosis, triggered mitotic catastrophe in EMT-resistant cells, and suppressed tumor growth in vivo.	[207]
AURKB inhibitor (ZM447439) + IGF1R inhibitor (OSI-906)	In vitro	A549	Dual inhibition disrupted IGF1R-AURKB signaling convergence, leading to mitotic errors and apoptosis.	Strong synergy with marked reduction in cell viability (CI = 0.32–0.35); OSI-906 potentiated ZM447439 cytotoxicity.	[210]
AURKB inhibitor (AZD1152-HPQA) + radiotherapy	In vitro and in vivo	H460, A549, H520, H661 (NSCLC)	Inhibition of AURKB suppressed repopulation after irradiation by inducing polyploidy and loss of clonogenic potential.	Enhanced radiation efficacy.	[211]
Multi-kinase inhibitor S49076 (targets MET, AXL, FGFR1–3, and AURKB) + radiotherapy	In vitro and in vivo	NSCLC cell lines (H441, A549, H460) and orthotopic xenografts (H460-luc, A549-luc)	S49076 inhibits AURKB and reduces histone H3 phosphorylation; combination with ionizing radiation enhances DNA damage response and limits tumor repopulation independent of MET dependency.	Additive effects on clonogenic survival in vitro; enhanced tumor growth delay and prolonged survival in vivo.	[214]
AURKB inhibitor (barasertib) + multi-kinase inhibitor (foretinib)	In vitro	MYC-amplified SCLC cell lines (16HV, 86M1 and H524)	Foretinib partially inhibited AURKB and MEK/FER signaling; co-treatment amplified apoptosis via PARP1 and caspase-3 activation.	Strong synergistic apoptosis induction restricted to MYC-amplified cells; no synergy in non-MYC models.	[215]
Haspin inhibitor (CHR-6494) + Pan-aurora inhibitor (VX-680) or AUKB inhibitor (barasertib)	In vitro	NSCLC cell lines (A549, H358, H460, H1299, H1975)	-	KRAS-mutant lines showed higher sensitivity, confirming enhanced cytotoxicity via Aurora B–Haspin co-inhibition.	[217]
AURKB inhibitor (AZD2811) + BCL2 inhibitor (venetoclax)	In vitro and in vivo	SCLC cell lines overexpressing BCL2 (H1048, H69, SC101, SC96, LC-F-22, SC61) and BCL2-low lines (H446Vec, H1876Vec); xenograft/PDX models)	Venetoclax markedly sensitized BCL2-overexpressing cells to AZD2811 by restoring apoptosis, increasing caspase 3/7 activity, PARP cleavage, and DNA damage; minimal effect on BCL2-low cells.	Enhanced tumor growth inhibition in BCL2-high models compared with single agents; sustained responses observed after treatment cessation.	[218]
AURKB inhibitor (AZD2811) + PD-L1 inhibitor (durvalumab)	Phase II clinical trial	Relapsed SCLC	-	Terminated due to serious unexpected adverse effects reported in other clinical trials using the same drug.	NCT04525391
AURKB inhibitor (AZD2811) + PD-L1 inhibitor (durvalumab)	Phase II clinical trial	Extensive SCLC	-	Active, not recruiting.	NCT04745689

AURKB: Aurora kinase B; BCL2: B-cell lymphoma 2; BIM: BCL2-like 11; CI: combination index; EGFR: epidermal growth factor receptor; EMT: epithelial–mesenchymal transition; FER: feline sarcoma-related protein; FOXO1/3: forkhead box O1/3; H3S10: histone H3 serine 10; IGF1: insulin-like growth factor 1; MEK: mitogen-activated protein kinase kinase; NSCLC: non-small cell lung cancer; PARP1: poly (ADP-ribose) polymerase 1; PD-L1: programmed death-ligand 1; PUMA: p53 upregulated modulator of apoptosis; SCLC: small cell lung cancer.

### 5.4. MPS1 (TTK) Inhibition-Based Combination Therapies in Lung Cancer

Targeting mitotic kinases such as MPS1 has been explored as a strategy to enhance therapeutic efficacy and overcome resistance mechanisms in lung cancer. Given the limited benefit of MPS1 inhibition as monotherapy, recent studies have focused on combination approaches to potentiate cytotoxicity and improve selectivity (Table 7).

One promising approach involves combining the selective MPS1 inhibitor BAY1217389 with the BH3-mimetic navitoclax to promote apoptotic priming. In NSCLC models, BAY1217389 alone exhibited limited efficacy, as cells were able to survive after premature mitotic exit. The addition of navitoclax, which inhibits anti-apoptotic BCL-2 family proteins, abrogated this survival by promoting apoptosis shortly after mitotic exit. Mechanistically, BAY1217389 shortened mitotic duration and accelerated mitotic exit, consistent with a mitotic driver. Co-treatment with navitoclax did not alter mitotic timing but substantially increased post-mitotic cell death, indicating enhanced apoptotic sensitivity. Importantly, sub-IC_50_ concentrations of both agents induced robust post-mitotic apoptosis, elevated caspase-9 activation, and markedly reduced cell survival, while exhibiting lower cytotoxicity toward non-tumoral epithelial cells [20].

ABC transporters, such as ABCG2, have been extensively studied for their strong association with multidrug resistance in the cancer context, including lung cancer [219]. Another study investigated CC-671, a dual MPS1/CDC like kinase 2 (CLK2) inhibitor, and its ability to overcome multidrug resistance mediated by ABCG2 overexpression in NSCLC. Mechanistically, CC-671 increased the intracellular accumulation of ABCG2 substrate drugs, mitoxantrone and topotecan, by inhibiting ABCG2 efflux activity, thereby resensitizing resistant lung cancer cells (NCI-H460/MX20 and A549/MX10) to cytotoxic agents. Importantly, CC-671 did not affect ABCG2 expression or localization, and computational docking suggested a direct interaction with the transporter’s drug-binding site. These results support the potential use of CC-671 in combination with standard chemotherapeutics to reverse ABCG2-mediated resistance in NSCLC [167].

Loss of STING signaling is a hallmark of KRAS/LKB1 (KL)-mutant NSCLC cells, where its epigenetic silencing impairs cytotoxic T-cell infiltration and contributes to resistance to immune checkpoint blockade [220]. A recent study has shown that MPS1 inhibitors, including CFI-402257, BAY1217389, and CC-671, can restore tumor immunogenicity in KRAS/LKB1-mutant NSCLC models, but only after Stimulator of Interferon Genes (STING) reactivation. In STING^Low^ or STING^Absent^ KL cells, pretreatment with DNMT inhibitors (decitabine, DAC) and/or the EZH2 inhibitor GSK126 re-established STING expression, thereby enabling MPS1 inhibition to induce CXCL10 and IFN-β secretion, TBK1/STAT1 activation, and MHC class I upregulation. This sequential epigenetic priming followed by MPS1 inhibition enhanced CD8^+^ T-cell and NK cell infiltration, increased granzyme B production, and converted immune “cold” tumors into “hot” phenotypes. In murine KL models, DAC priming combined with pulse MPS1 inhibition reactivated STING, promoted CXCL10 expression, and redistributed CD8^+^ T cells within the tumor microenvironment, resulting in significant tumor suppression in a STING- and CD8^+^ T cell-dependent manner. These findings highlight that epigenetic reprogramming is essential for MPS1 inhibitors to overcome immunotherapy resistance and re-engage antitumor immunity in STING-silenced KL NSCLC [221].

In a phase I study, the MPS1 inhibitor BAY1217389 was combined with weekly paclitaxel in patients with advanced solid tumors, including NSCLC. The combination established a tolerable dose with myelosuppression as the dose-limiting toxicity, confirmed target engagement, and showed preliminary antitumor activity, with objective responses observed in 31.6% of evaluable patients [222]. Another MPS1 inhibitor, BOS172722, is being evaluated with paclitaxel in a phase I trial for advanced non-hematologic malignancies, with no published results to date (NCT03328494).

**Table 7:** Preclinical Combination Strategies Targeting MPS1 in Lung Cancer Models.

Combination Strategy	Assay Type	Cancer Model	Mechanistic Insight/ Proposed Synergy	Main Reported Outcome	Reference
MPS1 inhibitor (BAY1217389) + BH3-mimetic (navitoclax)	In vitro	A549 cell line and HPAEpiC non-tumor cells	BAY1217389, acting as a mitotic driver, accelerating mitotic exit; navitoclax enhances caspase-9 activation and promotes post-mitotic death	Increased post-mitotic apoptosis and reduced clonogenic survival. Induced minimal cytotoxic effects in non-malignant cells	[20]
Dual MPS1/CLK2 inhibitor (CC-671) + chemotherapeutic agents (mitoxantrone, topotecan)	In vitro	NSCLC resistant cell lines (NCI-H460/MX20, A549/MX10)	CC-671 inhibited ABCG2 efflux activity without affecting expression or localization, increasing intracellular accumulation of substrate drugs and reversing multidrug resistance	Restored sensitivity of ABCG2-overexpressing NSCLC cells to cytotoxic agents	[167]
MPS1 inhibitors (CFI-402257, BAY1217389, CC-671) + epigenetic inhibitors (DNMT inhibitor decitabine ± EZH2 inhibitor GSK126)	In vitro and in vivo	KRAS/LKB1-mutant NSCLC	Epigenetic priming reactivated STING signaling, enabling MPS1 inhibition to induce CXCL10 and IFN-β secretion, TBK1/STAT1 activation, and MHC-I upregulation, enhancing CD8^+^ and NK cell infiltration	Restored tumor immunogenicity and suppressed tumor growth in a CD8^+^ T cell- and STING-dependent manner	[221]
MPS1 inhibitor (BAY1217389) + paclitaxel	Phase I clinical trial	Advanced solid tumors/including NSCLC	Paclitaxel enhances cytotoxicity of MPS1 inhibition; target engagement confirmed	Established tolerable dose; myelosuppression as dose-limiting toxicity; 31.6% objective responses in evaluable patients	[222]
MPS1 inhibitor (BOS172722) + paclitaxel	Phase I clinical trial	Advanced non-hematologic malignancies	-	Complete with no published results	NCT03328494

ABCG2: ATP-binding cassette sub-family G member 2; CLK2: CDC-like kinase 2; IFN-β: interferon beta; KRAS: Kirsten rat sarcoma viral oncogene homolog; LKB1: liver kinase B1; MHC-I: major histocompatibility complex class I; MPS1: monopolar spindle 1 kinase; NK: natural killer; NSCLC: non-small cell lung cancer; STAT1: signal transducer and activator of transcription 1; TBK1: TANK-binding kinase 1.

Collectively, these findings emphasize that MPS1 inhibition alone is insufficient as a monotherapy but can serve as a powerful sensitizer in rationally designed combinations. Pairing MPS1 inhibitors with apoptosis inducers, efflux pump reversal agents, or epigenetic modulators not only enhances direct cytotoxicity but also reprograms tumor–immune interactions. This positions MPS1 inhibition as a versatile therapeutic backbone to improve outcomes in lung cancer, particularly in genetically or immunologically refractory contexts.

### 5.5. CENP-E Inhibition-Based Combination Therapies in Lung Cancer

Targeting CENP-E, a kinesin motor protein essential for chromosome alignment and segregation, has emerged as a promising therapeutic strategy in lung cancer. Nevertheless, CENP-E inhibition as a single agent often results in limited efficacy due, mainly, to mitotic slippage and post-mitotic survival. Consequently, preclinical studies have investigated rational combination strategies to amplify the cytotoxic consequences of CENP-E inhibition by disabling compensatory survival mechanisms, priming mitochondria for apoptosis during mitotic arrest, or counteracting immune evasion pathways (Table 8).

The combination of the CENP-E inhibitor, GSK923295, with the BH3-mimetic, navitoclax, demonstrated strong synergistic cytotoxicity in NSCLC models. While GSK923295 alone induced mitotic arrest, as shown by increased cyclin B1 levels, a substantial fraction of cells underwent mitotic slippage. The addition of navitoclax shifted this fate toward apoptosis during mitosis, accelerating cell death and inducing caspase-9 activation, ultimately resulting in a marked reduction of clonogenic survival at sub-IC_50_ concentrations. Importantly, this effect was selective for cancer cells, sparing non-tumor HPAEpiC cells. In 3D spheroid models, GSK923295 plus navitoclax further enhanced apoptotic cell death and disrupted spheroid architecture compared with single agents [20]. Immune evasion has also been identified as a potential resistance mechanism following CENP-E inhibition. Treatment with the CENP-E inhibitor GSK923295 could upregulate PD-L1 expression in A549 cells, contributing to an immunosuppressive microenvironment. However, co-treatment with the anti-PD-L1 antibody atezolizumab enhanced the antitumor response, resulting in a greater reduction in colony formation compared with either agent alone. In vivo, CENP-E knockdown combined with atezolizumab further suppressed tumor growth and significantly prolonged survival in Lewis lung carcinoma–bearing mice. Mechanistically, the combination promoted infiltration of effector CD8^+^ T cells and diminished tumor-infiltrating Treg populations, thereby reinforcing immune-mediated tumor control [16].

Overall, preclinical findings indicate that integrating CENP-E inhibitors with BH3-mimetics or immune checkpoint inhibitors can markedly enhance antitumor efficacy in NSCLC models. These combinations collectively increase mitotic arrest, trigger apoptotic signaling, and reinforce antitumor immune responses, thereby overcoming resistance mechanisms associated with mitotic escape and immune suppression.

**Table 8:** Preclinical Combination Strategies Targeting CENP-E in Lung Cancer Models.

Combination Strategy	Assay Type	Cancer Model	Mechanistic Insight/ Proposed Synergy	Main Reported Outcome	Reference
CENP-E inhibitor (GSK923295) + BH3-mimetic (navitoclax)	In vitro	A549 cell line and HPAEpiC non-tumor cells	GSK923295 induces mitotic arrest and increases cyclin B1; navitoclax accelerates apoptosis during mitosis, prevents mitotic slippage, and enhances caspase-9-mediated activation of the intrinsic apoptotic pathway.	Reduced clonogenic survival, increased mitotic cell death and induced apoptosis. Exhibited lower cytotoxicity in non-tumorigenic cells.	[20]
CENP-E inhibitor (GSK923295) + anti–PD-L1 antibody (atezolizumab)	In vitro and in vivo	A549 cell line and murine xenograft models	GSK923295 induced PD-L1 expression, contributing to an immunosuppressive phenotype; PD-L1 blockade enhanced CD8^+^ T-cell infiltration and reduced Treg populations.	Reduced colony formation in vitro, suppressed tumor growth, and improved survival in vivo.	[16]

CENP-E: centromere-associated protein E; PD-L1: programmed death-ligand 1.

### 5.6. Eg5 Inhibition-Based Combination Therapies in Lung Cancer

Targeting Eg5 has emerged as a promising strategy in lung cancer. However, as with other mitotic kinesin inhibitors, monotherapy often exhibits limited efficacy due to cell cycle arrest without sufficient apoptosis, allowing for post-mitotic survival. Preclinical studies have therefore explored combination strategies to potentiate the cytotoxic effects of Eg5 inhibition, either by sensitizing cells to apoptosis, enhancing DNA damage responses, or overcoming chemoresistance mechanisms (Table 9) [223,224,225,226].

Several studies have demonstrated that combining Eg5 inhibitors with BH3-mimetics can significantly enhance apoptosis in lung cancer cells. Sequential or simultaneous treatment of SCLC cells with the Eg5 inhibitor SB743921 and the selective BCL-xL inhibitor WEHI-539 resulted in markedly increased apoptosis and reduced cell viability compared with either agent alone [224]. Similarly, in EGFR-independent LUAD sublines such as HCC827 GR2 and H1975 WR7, dual inhibition of Eg5 and BCL-xL induced extensive apoptosis, whereas Eg5 silencing alone primarily caused G2/M arrest without significant cell death. This strategy also proved effective in KRAS-mutant LUAD cells, highlighting its potential across genetically diverse lung cancer models [223].

Beyond apoptotic priming, Eg5 inhibition can enhance chemosensitivity in resistant lung cancer cells. In cisplatin-resistant LUAD cell lines, single-agent treatment with either cisplatin or a Eg5 inhibitor (trans-24) produced limited cytotoxic effects, whereas their combination markedly suppressed cell growth and clonogenic potential. Mechanistically, Eg5 inhibition overcomes chemoresistance by downregulating BRCA1 and cyclin B1, proteins that normally support DNA repair and mitotic progression, suggesting that Eg5 may intersect with DNA damage response pathways to modulate cisplatin sensitivity [225].

Additional preclinical work has identified synergistic combinations with multitarget antimitotic agents. CRx-026, a combination of the Eg5 inhibitor chlorpromazine and pentamidine, disrupts mitosis at multiple stages: chlorpromazine blocks Eg5, causing monopolar spindles, mitotic arrest and mitotic cell death, while pentamidine delays anaphase progression, leading to defective chromosome segregation, DNA bridges, and activation of DNA damage responses. CRx-026 also synergizes with microtubule-binding agents such as paclitaxel. In A549 cells, a fixed 1:2 chlorpromazine/pentamidine ratio combined with paclitaxel significantly inhibited proliferation in vitro, and in xenograft models, the combination reduced tumor growth more effectively than either agent alone [226]. A Phase 1 clinical trial evaluated the combination of the Eg5 inhibitor ARRY-520 with granulocyte-colony stimulating factor (G-CSF) in patients with advanced solid tumors (NCT00462358). However, no results from this study have been published to date.

**Table 9:** Preclinical Combination Strategies Targeting Eg5 in Lung Cancer Models.

Combination Strategy	Assay Type	Cancer Model	Mechanistic Insight/ Proposed Synergy	Main Reported Outcome	Reference
Eg5 inhibitor (SB743921) + BCL-xL inhibitor (WEHI-539)	In vitro	SCLC cell lines (Lu-135 and H69)	BH3-mimetic-mediated apoptotic priming enhances cell death following mitotic arrest	Markedly increased apoptotic activity and reduced cell viability compared with single-agent therapy	[224]
Eg5 inhibitor (SB743921) + BCL-xL inhibitor (WEHI-539)	In vitro	EGFR-independent (HCC827 GR2, H1975 and WR7) and KRAS-mutant (H441) LUAD cell lines	Eg5 inhibition alone induces G2/M arrest without significative cell death, but dual inhibition triggers mitochondrial apoptosis	Induced extensive apoptosis, surpassing the cytostatic effect of Eg5 inhibition alone	[223]
Eg5 inhibitor (trans-24) + cisplatin	In vitro	Cisplatin-resistant LUAD cell lines (A549-DDP and H1299-DDP)	Eg5 inhibition downregulates BRCA1 and cyclin B1, impairing DNA repair and enhancing DNA damage sensitivity	Enhanced cisplatin sensitivity and markedly reduced clonogenic survival, indicating reversal of chemoresistance	[225]
Eg5 inhibitor (Chlorpromazine) + Pentamidine (CRx-026) ± paclitaxel	In vitro and In vivo	A549 cells and murine xenograft models	Chlorpromazine blocks Eg5, causing monopolar spindle formation, mitotic arrest, and cell death; pentamidine interferes with anaphase progression, leading to chromosome mis-segregation events and activation of DNA damage responses. Enhanced the cytotoxic effect of paclitaxel	Strongly inhibited cell proliferation in vitro and reduced tumor growth more effectively than individual treatments in vivo	[226]
Eg5 inhibitor (ARRY-520) + G-CSF (Filgrastim)	Phase 1 clinical trial	Advanced solid tumors	-	Complete with no published results	NCT00462358

EGFR: epidermal growth factor receptor; G-CSF: granulocyte-colony stimulating factor; LUAD: lung adenocarcinoma; SCLC: small cell lung cancer.

Overall, preclinical evidence indicates that Eg5 inhibitors, when used in combination with BH3-mimetics, DNA-damaging agents, or other mitotic inhibitors, substantially enhance anticancer efficacy in lung cancer models. These strategies not only increase apoptotic cell death but also overcome resistance mechanisms associated with mitotic arrest and chemoresistance.

## 6. Conclusions and Future Perspectives

Targeting the mitotic machinery represents a compelling therapeutic strategy in lung cancer, given the central role of mitotic kinases and motor proteins in ensuring faithful chromosome segregation and cell division. While monotherapies with inhibitors of PLK1, AURKA, AURKB, MPS1, CENP-E, or Eg5 have shown potent antitumor activity in preclinical models, clinical translation has been limited by toxicity, narrow therapeutic windows, and modest efficacy as single agents. These limitations underscore the need for combinatorial approaches that exploit tumor vulnerabilities while mitigating resistance mechanisms.

Combination therapies that integrate mitotic inhibitors with standard chemotherapeutics, targeted therapies, or immunotherapies have demonstrated enhanced efficacy in preclinical studies. By simultaneously disrupting mitotic progression and complementary oncogenic or survival pathways, these strategies can potentiate tumor cell death, overcome adaptive resistance, and potentially extend clinical benefit. Moreover, the use of FDA-approved agents in these combinations can accelerate clinical translation and reduce development costs, facilitating more rapid evaluation in patients.

Despite these promising avenues, several challenges remain. First, the intrinsic redundancy and compensatory mechanisms within mitotic and cell cycle pathways may limit the efficacy of targeted combinations. Second, the potential for additive or synergistic toxicity poses a significant hurdle in clinical translation, necessitating careful dose optimization and scheduling. Third, heterogeneity within tumors may result in variable responses to mitotic inhibition, highlighting the importance of integrating single-cell and functional genomic analyses into trial design.

Future research should prioritize the following: (i) the rational design of combination regimens informed by mechanistic insights into mitotic dependencies; (ii) robust preclinical models that recapitulate tumor heterogeneity and therapeutic resistance; and (iii) clinical trials employing adaptive designs to optimize dosing, sequence, and combinatorial partners. Advances in high-throughput genomics, single-cell profiling, and CRISPR-based functional screens will continue to facilitate the identification of novel vulnerabilities and synergistic combinations, paving the way for more personalized and durable therapeutic strategies targeting the mitotic machinery in lung cancer.

In conclusion, the strategic combination of mitotic inhibitors with complementary therapies represents a promising avenue in oncology. By leveraging insights from precision medicine, including biomarker-guided patient selection, molecular profiling, and functional assays, therapeutic regimens can be tailored to exploit tumor-specific vulnerabilities. It is important to recognize that mitotic proteins are essential for normal cell division, and therefore, targeting these pathways can affect both cancer and normal proliferating cells [227], presenting biosafety considerations. However, this challenge is not unique to mitotic inhibitors; many clinically successful chemotherapeutics, such as Paclitaxel, act by disrupting microtubule dynamics and similarly affect both malignant and normal cells [228,229]. Such lack of specificity can be partially overcome through innovative delivery strategies, including nanotechnology-based systems, which allow preferential accumulation in cancer cells, increasing the therapeutic window while minimizing systemic toxicity [230]. With careful mechanistic understanding, real-time monitoring of response, and optimization of dosing and scheduling, this approach has the potential to improve outcomes for patients with lung cancer and other malignancies characterized by mitotic dysregulation, while minimizing toxicity and maximizing clinical benefit.

## Figures and Tables

**Table 1:** Currently approved drugs for lung cancer treatment.

Drug	Drug Class/Target	Indication	Standard Dosage	Most Common Adverse Effects	References
Cisplatin	DNA-directed alkylating agent	Advanced lung cancer	120 mg/m^2^ i.v. every 21 days	Renal toxicity, thrombocytopenia, nausea and vomiting	[32]
Carboplatin	DNA-directed alkylating agent	SCLC	300–400 mg/m^2^ i.v. monthly	Nausea or vomiting (48%), leukopenia (39%), thrombocytopenia (18%) and anemia (18%)	[33]
Paclitaxel	Taxane chemotherapy	Advanced NSCLC	200 mg/m^2^ i.v. every 21 days	Alopecia (68.0%), nausea (56.0%), diarrhea (36.0%) and Hematologic AEs	[34]
Docetaxel	Taxane chemotherapy	Advanced or metastatic NSCLC	75 mg/m^2^ i.v. every 21 days	Grade 3/4 AEs (~67%), neutropenia (67.3%), fever (61.8%), asthenia (54.5%)	[35]
Pemetrexed	Antimetabolite chemotherapy	Advanced or metastatic nonsquamous NSCLC	500 mg/m^2^ i.v. every 21 days	Leukopenia (9.6%), neutropenia (9.6%), anemia (6.0%)	[36]
Gemcitabine	Antimetabolite chemotherapy	Extensive SCLC	1000–1250 mg/m^2^ i.v. once a week for 21 days followed by one week rest period.	Grade 3/4 neutropenia (18.0%), nausea (72.4%), vomiting (34.5%) and anorexia (27.6%)	[37]
Vinorelbine	Vinca alkaloid	Advanced NSCLC	30 mg/m^2^ i.v. weekly	Neutropenia (89.0%), anemia (84.0%), anorexia (49.0%), weight loss (49.0%)	[38]
Etoposide	Topoisomerase II inhibitor	Advanced SCLC	125–140 mg/m^2^ i.v. (days 1, 3, and 5) every 4–5 weeks	Mild nausea and vomiting (30.8%), alopecia (30.8%), and leukopenia (30.8%)	[39]
Osimertinib	EGFR TKI	NSCLC EGFR-mutant	80 mg orally daily	Grade 3/4 AEs (30.5%), rash or acne (78.0%), diarrhea (57.0%) dry skin (36.0%), paronychia (33.0%)	[24]
Lurbinectedin	DNA-directed alkylating agent/transcription inhibitor	Metastatic SCLC (relapsed after platinum-based therapy)	3.2 mg/m^2^ i.v. infusion once every 21 days	Anemia (95.2%), creatinine increase (83.0%), leucopenia (79.0%)	[40]
Brigatinib	ALK TKI	Advanced ALK-positive NSCLC	180 mg once daily (after a 7-day lead-in at 90 mg/day)	Grade 3/4 AEs (78.0%), diarrhea (58.0%), increased blood creatine phosphokinase (50.0%), cough (36.0%)	[41]
Topotecan	Topoisomerase I inhibitor	RR (relapsed/refractory) SCLC	1.5 mg/m^2^ i.v. (days 1–5) every 21 days	Grade 3/4 AEs (86.4%), anemia (65.9%), neutropenia (51.1%), asthenia (28.4%)	[42]
Irinotecan	Topoisomerase I inhibitor	RR (relapsed/refractory) SCLC	350 mg/m^2^ i.v. (day 1) every 21 days	Grade 3/4 AEs (69.5%), diarrhea (62.0%), nausea (47.1%), vomiting (30.5%)	[42]
Gefitinib	EGFR TKI	NSCLC EGFR-mutant	250 mg orally daily	Grade 3/4 AEs (35.8%), skin and subcutaneous tissues disorders (60.0%), ALT increase (55.8%), AST increase (54.0%)	[43]
Aumolertinib	EGFR TKI	NSCLC EGFR-mutant	110 mg orally daily	Grade 3/4 AEs (36.4%), infections (50.0%), GI disorders (48.6%), AST increase (29.9%), ALT increase (29.4%)	[43,44]
Erlotinib	EGFR TKI	NSCLC EGFR-mutant (exon 19 microdeletions or exon 21 L858R point mutation)	150 mg orally daily	Rash (58.1%), diarrhea (27.4%), dry skin (17.7%)	[45]
Mobocertinib	EGFR TKI	Advanced or metastatic NSCLC with EGFR exon 20 insertion mutations	160 mg orally daily	Grade 3/4 AEs (66.0%), diarrhea (91%), rash (45%), paronychia (38%)	[46]
Alectinib	ALK TKI	NSCLC ALK-positive	600 mg orally twice daily	Grade 3/4 AEs (26%), constipation (35%), nasopharyngitis (20%), dysgeusia (18%)	[47]
Crizotinib	ALK and ROS1 TKI	NSCLC ALK or ROS1-positive	250 mg orally twice daily	Grade 3/4 AEs (52%), nausea (74%), diarrhea (73%), vomiting (58%)	[47]
Ceritinib	ALK TKI	ALK-positive NSCLC post-crizotinib resistance	450 mg orally daily	Grade 3/4 AEs (86%), hypercholesterolemia (54.5%), diarrhea (47.7%), nausea (45.5)	[48]
Lorlatinib	ALK TKI	Advanced ALK-positive NSCLC	100 mg orally daily	Grade 3/4 AEs (86%), hypercholesterolemia (72%), hypertriglyceridemia (66%)	[49]
Entrectinib	ROS1 TKI	NSCLC ROS1 fusion-positive	600 mg orally daily	Grade 3/4 AEs (42.9%), dysgeusia (40.6%), dizziness (37.0%), constipation (31.7%)	[50]
Repotrectinib	ROS1 TKI	NSCLC ROS1 fusion-positive	160 mg twice daily (after a 14-day lead-in at 160 mg orally daily)	Grade 3/4 AEs (29.0%) dizziness (60.0%), dysgeusia (54.0%), paresthesia (35.0%)	[51]
Cemiplimab	PD-L1 inhibitor	Advanced NSCLC	350 mg i.v. every 21 days	Grade 3/4 AEs (47.0%), anemia (20.1%), decreased appetite (14.9%), fatigue (12.9%)	[52]
Fam-trastuzumab deruxtecan-nxki	Antibody–drug conjugate comprising a humanized anti-HER2 IgG1 monoclonal antibody (MAAL-9001) covalently linked to a topoisomerase I inhibitor (MAAA-118d, DXd) via a cleavable linker.	HER2-mutated NSCLC	5.4 mg/kg i.v. every 21 days	Grade 3/4 AEs (1.0%), nausea, decreased white blood cell count, decreased hemoglobin (≥20%)	[53]
Pralsetinib	RET fusion inhibitor	RET fusion-positive NSCLC	400 mg once daily	Grade 3/4 AEs (41.7%), leukopenia (61.1%), constipation (61.1%), decreased red blood cells (44.4%)	[54]
Selpercatinib	RET fusion inhibitor	RET-fusion—positive NSCLC	160 mg orally twice daily	Grade 3/4 AEs (70.3%), AST increase (61%), ALT increase (60%), hypertension (48%)	[55]
Adagrasib	RET fusion inhibitor	RET-fusion—positive NSCLC	400 mg orally daily	Grade 3/4 AEs (54.4%), neutropenia (46.0%), AST increase (41.0%), anemia (38.0%)	[56]
Tepotinib	MET TKI	MET exon14 skipping NSCLC	500 mg orally daily	Grade 3/4 AEs (61.3%), peripheral edema (62.3%), blood creatinine increase (38.7%), diarrhea (32.1%).	[57]
Capmatinib	MET TKI	MET exon14 skipping NSCLC	400 mg orally twice daily	Grade 3/4 AEs (~ 40%), lower extremity edema (65.0%), fatigue (35.0%), amylase, cratinine and lipase increase (20%)	[58]
Sotorasib	KRAS G12C inhibitor	KRAS G12C-mutated advanced NSCLC.	960 mg orally daily	Grade 3/4 AEs (61.5%), diarrhea (39.4%), nausea (23.1%)	[59]
Pembrolizumab	PD-1 inhibitor	Advanced or metastatic NSCLC	200 mg i.v. every 3 weeks	Grade 3/4 AEs (24.4%), hypertension (10,6%), hypothyroidism (9.6%)	[60]
Nivolumab	PD-1 inhibitor	NSCLC and SCLC	240 mg/kg i.v. every 2 weeks (for NSCLC patients) or 3 mg/kg i.v. every 2 weeks (for SCLC patients)	For NSCL patients: fatigue (30.8%), creatinine increase (26.2%), anemia (26.2%) For SCLC patients: Grade 3/4 AEs (12.9%), fatigue (12.2%), pruritus (9.5%), arthralgia and infusion-related reaction (6.1%)	[61,62]
Atezolizumab	PD-L1 inhibitor	Advanced or metastatic NSCLC	1200.0 mg i.v. every 3 weeks	Grade 3/4 AEs (31.5%); systemic infusion-related reactions (3.2%)	[63]
Durvalumab	PD-L1 inhibitor	NSCLC	10 mg/kg i.v. every 2 weeks	Grade 3/4 AEs (32%), fatigue (37.0%), nausea (24.0%), anorexia (19.0%)	[64]
Tarlatamab	Bispecific T-cell engager (DLL3)	Relapsed/refractory SCLC	1 mg orally (day 1) + 10 mg (days 8 and 15) + 10 mg every 2 weeks thereafter in 28-day cycles	Grade 3/4 AEs (54%), cytokine release syndrome (56%), decreased appetite (35%), anemia (31%)	[31]

AEs: adverse effects; ALK: anaplastic lymphoma kinase; ALT: alanine aminotransferase; AST: aspartate aminotransferase; DLL3: delta-like ligand 3; GI: gastrointestinal; MET: mesenchymal–epithelial transition factor; NSCLC: non-small cell lung cancer; ROS1: ROS1 proto-oncogene receptor tyrosine kinase; SCLC: small cell lung cancer; TKI: tyrosine kinase inhibitor.

## Data Availability

No new data were created or analyzed in this study.

## Abbreviations

The following abbreviations are used in this manuscript:

ABC	ATP-binding cassette
AE	Adverse event
ALK	Anaplastic lymphoma kinase
AMPK	AMP-activated protein kinase
AP-1	Activator protein 1
APC/C	Anaphase-promoting complex/cyclosome
AURKA	Aurora kinase A
AURKB	Aurora kinase B
BCRP	Breast cancer resistance protein
BRAF	B-Raf proto-oncogene, serine/threonine kinase
BRCA	Breast cancer susceptibility protein
Cdc20	Cell division cycle 20
CDK	Cyclin-dependent kinase
CENP-E	Centromere-associated protein E
CNS	Central nervous system
CPC	Chromosomal passenger complex
CR	Complete response
CTLA-4	Cytotoxic T-lymphocyte-associated protein 4
DIM	Dead in mitosis
DLL3	Delta-like ligand 3
DNA	Deoxyribonucleic acid
EGFR	Epidermal growth factor receptor
EMT	Epithelial–mesenchymal transition
ES-SCLC	Extensive-stage small cell lung cancer
FoxM1	Forkhead box M1
GI	Gastrointestinal
HER2	Human epidermal growth factor receptor 2
ICI	Immune checkpoint inhibitor
IL	Interleukin
KRAS	Kirsten rat sarcoma viral oncogene homolog
KSP	Kinesin spindle protein
LD-SCLC	Limited-stage small cell lung cancer
LUAD	Lung adenocarcinoma
Mad	Mitotic arrest-deficient
MAPK	Mitogen-activated protein kinase
MCC	Mitotic checkpoint complex
MDR1	Multidrug resistance protein 1
MET	Mesenchymal–epithelial transition factor
MHC	Major histocompatibility complex
MPS1	Monopolar spindle 1 kinase
mTOR	Mechanistic target of rapamycin
NK	Natural killer
NSCLC	Non-small cell lung cancer
NTRK	Neurotrophic tyrosine receptor kinase
OS	Overall survival
PD	Progressive disease
PD-1	Programmed cell death protein 1
PD-L1	Programmed death-ligand 1
PFS	Progression-free survival
PI3K	Phosphoinositide 3-kinase
PLK1	Polo-like kinase 1
PMD	Post-mitotic death
PR	Partial response
PTEN	Phosphatase and tensin homolog
RB1	Retinoblastoma protein
RET	Rearranged during transfection
ROS1	ROS proto-oncogene 1 receptor tyrosine kinase
SAC	Spindle assembly checkpoint
SCLC	Small cell lung cancer
SD	Stable disease
SNAI	Snail family transcriptional repressor
STAT	Signal transducer and activator of transcription
TGF-β	Transforming growth factor beta
TKI	Tyrosine kinase inhibitor
TP53	Tumor protein p53
VEGF	Vascular endothelial growth factor

## References

[1] Bray F., Laversanne M., Sung H., Ferlay J., Siegel R.L., Soerjomataram I., Jemal A. (2024). Global cancer statistics 2022: GLOBOCAN estimates of incidence and mortality worldwide for 36 cancers in 185 countries. CA A Cancer J. Clin..

[2] Li C., Lei S., Ding L., Xu Y., Wu X., Wang H., Zhang Z., Gao T., Zhang Y., Li L. (2023). Global burden and trends of lung cancer incidence and mortality. Chin. Med. J..

[3] Kenaan N., Hanna G., Sardini M., Iyoun M.O., Layka K., Hannouneh Z.A., Alshehabi Z. (2024). Advances in early detection of non-small cell lung cancer: A comprehensive review. Cancer Med..

[4] Garg P., Singhal S., Kulkarni P., Horne D., Malhotra J., Salgia R., Singhal S.S. (2024). Advances in Non-Small Cell Lung Cancer: Current Insights and Future Directions. J. Clin. Med..

[5] Tang F.H., Wong H.Y.T., Tsang P.S.W., Yau M., Tam S.Y., Law L., Yau K., Wong J., Farah F.H.M., Wong J. (2025). Recent advancements in lung cancer research: A narrative review. Transl. Lung Cancer Res..

[6] Zhai X., Zhang Z., Chen Y., Wu Y., Zhen C., Liu Y., Lin Y., Chen C. (2025). Current and future therapies for small cell lung carcinoma. J. Hematol. Oncol..

[7] Falchero L., Meyer N., Molinier O., Al Freijat F., Pegliasco H., Lecuyer E., Stoven L., Belmont L., Loutski S., Maincent C. (2024). Real-life nationwide characteristics and outcomes of small cell lung cancer over the last 20 years: Impact of immunotherapy on overall survival in a real-life setting. Eur. J. Cancer.

[8] Nie Y., Schalper K.A., Chiang A. (2024). Mechanisms of immunotherapy resistance in small cell lung cancer. Cancer Drug Resist..

[9] Lim Z.F., Ma P.C. (2019). Emerging insights of tumor heterogeneity and drug resistance mechanisms in lung cancer targeted therapy. J. Hematol. Oncol..

[10] Smith S.L., Bowers N.L., Betticher D.C., Gautschi O., Ratschiller D., Hoban P.R., Booton R., Santibáñez-Koref M.F., Heighway J. (2005). Overexpression of aurora B kinase (AURKB) in primary non-small cell lung carcinoma is frequent, generally driven from one allele, and correlates with the level of genetic instability. Br. J. Cancer.

[11] Li H., Wang H., Sun Z., Guo Q., Shi H., Jia Y. (2017). The clinical and prognostic value of polo-like kinase 1 in lung squamous cell carcinoma patients: Immunohistochemical analysis. Biosci. Rep..

[12] Schneider M.A., Christopoulos P., Muley T., Warth A., Klingmueller U., Thomas M., Herth F.J., Dienemann H., Mueller N.S., Theis F. (2017). AURKA, DLGAP5, TPX2, KIF11 and CKAP5: Five specific mitosis-associated genes correlate with poor prognosis for non-small cell lung cancer patients. Int. J. Oncol..

[13] Al-Khafaji A.S., Davies M.P., Risk J.M., Marcus M.W., Koffa M., Gosney J.R., Shaw R.J., Field J.K., Liloglou T. (2017). Aurora B expression modulates paclitaxel response in non-small cell lung cancer. Br. J. Cancer.

[14] Zhu X., Li S., Ye L. (2026). The expression of tyrosine kinase and threonine promotes the progression of lung adenocarcinoma and is linked to a negative prognosis. Medicine.

[15] Hao X., Qu T. (2019). Expression of CENPE and its Prognostic Role in Non-small Cell Lung Cancer. Open Med..

[16] Liang J., Tian C., Liu L., Zeng X., Zhang Y. (2024). Targeting CENP-E augments immunotherapy in non-small cell lung cancer via stabilizing PD-L1. Int. Immunopharmacol..

[17] Wang Y., Liu J., Xu J., Ji Z. (2025). The expression and prognosis for Aurora kinases in human non-small cell lung cancer. Discov. Oncol..

[18] You L., Xu Y., Fu Y., Li J. (2026). PLK1 overexpression as a dual-role biomarker and therapeutic vulnerability in pulmonary adenocarcinoma. PeerJ.

[19] Al-Khafaji A.S.K., Marcus M.W., Davies M.P.A., Risk J.M., Shaw R.J., Field J.K., Liloglou T. (2017). AURKA mRNA expression is an independent predictor of poor prognosis in patients with non-small cell lung cancer. Oncol. Lett..

[20] Pinto B., Silva J.P.N., Silva P.M.A., Barbosa D.J., Sarmento B., Tavares J.C., Bousbaa H. (2023). Maximizing Anticancer Response with MPS1 and CENPE Inhibition Alongside Apoptosis Induction. Pharmaceutics.

[21] Novais P., Silva P.M.A., Amorim I., Bousbaa H. (2021). Second-Generation Antimitotics in Cancer Clinical Trials. Pharmaceutics.

[22] Khan S., Upadhyay S., Kauser S., Hasan G.M., Lu W., Waters M., Hassan M.I., Sohal S.S. (2025). Redefining the Diagnostic and Therapeutic Landscape of Non-Small Cell Lung Cancer in the Era of Precision Medicine. J. Clin. Med..

[23] Smolarz B., Łukasiewicz H., Samulak D., Piekarska E., Kołaciński R., Romanowicz H. (2025). Lung Cancer-Epidemiology, Pathogenesis, Treatment and Molecular Aspect (Review of Literature). Int. J. Mol. Sci..

[24] Tsuboi M., Weder W., Escriu C., Blakely C., He J., Dacic S., Yatabe Y., Zeng L., Walding A., Chaft J.E. (2021). Neoadjuvant osimertinib with/without chemotherapy versus chemotherapy alone for EGFR-mutated resectable non-small-cell lung cancer: NeoADAURA. Future Oncol..

[25] Bouchard N., Daaboul N. (2025). Lung Cancer: Targeted Therapy in 2025. Curr. Oncol..

[26] Hui Z., Zhang J., Ren Y., Li X., Yan C., Yu W., Wang T., Xiao S., Chen Y., Zhang R. (2022). Single-cell profiling of immune cells after neoadjuvant pembrolizumab and chemotherapy in IIIA non-small cell lung cancer (NSCLC). Cell Death Dis..

[27] Cui X., Liu S., Song H., Xu J., Sun Y. (2025). Single-cell and spatial transcriptomic analyses revealing tumor microenvironment remodeling after neoadjuvant chemoimmunotherapy in non-small cell lung cancer. Mol. Cancer.

[28] Blackhall F., Girard N., Livartowski A., McDonald L., Roset M., Lara N., Juarez García A. (2023). Treatment patterns and outcomes among patients with small-cell lung cancer (SCLC) in Europe: A retrospective cohort study. BMJ Open.

[29] Zhang T., Tao L., Chen Y., Zhang S., Liu Y., Li Y., Wang R. (2024). Evaluation of Efficacy and Safety in First-Line Treatment Methods for Extensive-Stage Small Cell Lung Cancer: A Comprehensive Comparative Study of Chemotherapy, Targeted Therapy Combined With Chemotherapy, and Immunotherapy Combined With Chemotherapy. Clin. Respir. J..

[30] Hummel H.D., Ahn M.J., Blackhall F., Reck M., Akamatsu H., Ramalingam S.S., Borghaei H., Johnson M., Dirnberger F., Cocks K. (2025). Patient-Reported Outcomes for Patients with Previously Treated Small Cell Lung Cancer Receiving Tarlatamab: Results from the DeLLphi-301 Phase 2 Trial. Adv. Ther..

[31] Mountzios G., Sun L., Cho B.C., Demirci U., Baka S., Gümüş M., Lugini A., Zhu B., Yu Y., Korantzis I. (2025). Tarlatamab in Small-Cell Lung Cancer after Platinum-Based Chemotherapy. N. Engl. J. Med..

[32] De Jager R., Longeval E., Klastersky J. (1980). High-dose cisplatin with fluid and mannitol-induced diuresis in advanced lung cancer: A phase II clinical trial of the EORTC Lung Cancer Working Party (Belgium). Cancer Treat. Rep..

[33] Smith I.E., Harland S.J., Robinson B.A., Evans B.D., Goodhart L.C., Calvert A.H., Yarnold J., Glees J.P., Baker J., Ford H.T. (1985). Carboplatin: A very active new cisplatin analog in the treatment of small cell lung cancer. Cancer Treat. Rep..

[34] Murphy W.K., Fossella F.V., Winn R.J., Shin D.M., Hynes H.E., Gross H.M., Davilla E., Leimert J., Dhingra H., Raber M.N. (1993). Phase II study of taxol in patients with untreated advanced non-small-cell lung cancer. J. Natl. Cancer Inst..

[35] Shepherd F.A., Dancey J., Ramlau R., Mattson K., Gralla R., O’Rourke M., Levitan N., Gressot L., Vincent M., Burkes R. (2000). Prospective randomized trial of docetaxel versus best supportive care in patients with non-small-cell lung cancer previously treated with platinum-based chemotherapy. J. Clin. Oncol. Off. J. Am. Soc. Clin. Oncol..

[36] Dittrich C., Papai-Szekely Z., Vinolas N., Sederholm C., Hartmann J.T., Behringer D., Kazeem G., Desaiah D., Leschinger M.I., von Pawel J. (2014). A randomised phase II study of pemetrexed versus pemetrexed+erlotinib as second-line treatment for locally advanced or metastatic non-squamous non-small cell lung cancer. Eur. J. Cancer.

[37] Cormier Y., Eisenhauer E., Muldal A., Gregg R., Ayoub J., Goss G., Stewart D., Tarasoff P., Wong D. (1994). Gemcitabine is an active new agent in previously untreated extensive small cell lung cancer (SCLC): A study of the National Cancer Institute of Canada Clinical Trials Group. Ann. Oncol..

[38] Jassem J., Ramlau R., Karnicka-Młodkowska H., Krawczyk K., Krzakowski M., Zatloukal P., Lemarié E., Hartmann W., Novakova L., O’Brien M. (2001). A multicenter randomized phase II study of oral vs. intravenous vinorelbine in advanced non-small-cell lung cancer patients. Ann. Oncol. Off. J. Eur. Soc. Med. Oncol..

[39] Eagan R.T., Carr D.T., Frytak S., Rubin J., Lee R.E. (1976). VP-16-213 versus polychemotherapy in patients with advanced small cell lung cancer. Cancer Treat. Rep..

[40] Trigo J., Subbiah V., Besse B., Moreno V., López R., Sala M.A., Peters S., Ponce S., Fernández C., Alfaro V. (2020). Lurbinectedin as second-line treatment for patients with small-cell lung cancer: A single-arm, open-label, phase 2 basket trial. Lancet Oncol..

[41] Camidge D.R., Kim H.R., Ahn M.J., Yang J.C.H., Han J.Y., Hochmair M.J., Lee K.H., Delmonte A., Garcia Campelo M.R., Kim D.W. (2021). Brigatinib Versus Crizotinib in ALK Inhibitor-Naive Advanced ALK-Positive NSCLC: Final Results of Phase 3 ALTA-1L Trial. J. Thorac. Oncol. Off. Publ. Int. Assoc. Study Lung Cancer.

[42] Edelman M.J., Dvorkin M., Laktionov K., Navarro A., Juan-Vidal O., Kozlov V., Golden G., Jordan O., Deng C.Q., Bentsion D. (2022). Randomized phase 3 study of the anti-disialoganglioside antibody dinutuximab and irinotecan vs. irinotecan or topotecan for second-line treatment of small cell lung cancer. Lung Cancer.

[43] Lu S., Dong X., Jian H., Chen J., Chen G., Sun Y., Ji Y., Wang Z., Shi J., Lu J. (2022). AENEAS: A Randomized Phase III Trial of Aumolertinib Versus Gefitinib as First-Line Therapy for Locally Advanced or MetastaticNon-Small-Cell Lung Cancer With EGFR Exon 19 Deletion or L858R Mutations. J. Clin. Oncol. Off. J. Am. Soc. Clin. Oncol..

[44] Shirley M., Keam S.J. (2022). Aumolertinib: A Review in Non-Small Cell Lung Cancer. Drugs.

[45] Markóczy Z., Sárosi V., Kudaba I., Gálffy G., Turay Ü.Y., Demirkazik A., Purkalne G., Somfay A., Pápai-Székely Z., Rásó E. (2018). Erlotinib as single agent first line treatment in locally advanced or metastatic activating EGFR mutation-positive lung adenocarcinoma (CEETAC): An open-label, non-randomized, multicenter, phase IV clinical trial. BMC Cancer.

[46] Gupta N., Largajolli A., Witjes H., Diderichsen P.M., Zhang S., Hanley M.J., Lin J., Mehta M. (2022). Mobocertinib Dose Rationale in Patients with Metastatic NSCLC with EGFR Exon 20 Insertions: Exposure-Response Analyses of a Pivotal Phase I/II Study. Clin. Pharmacol. Ther..

[47] Hida T., Nokihara H., Kondo M., Kim Y.H., Azuma K., Seto T., Takiguchi Y., Nishio M., Yoshioka H., Imamura F. (2017). Alectinib versus crizotinib in patients with *ALK*-positive non-small-cell lung cancer (J-ALEX): An open-label, randomised phase 3 trial. Lancet.

[48] Cho B.C., Kim D.-W., Bearz A., Laurie S.A., McKeage M., Borra G., Park K., Kim S.-W., Ghosn M., Ardizzoni A. (2017). ASCEND-8: A Randomized Phase 1 Study of Ceritinib, 450 mg or 600 mg, Taken with a Low-Fat Meal versus 750 mg in Fasted State in Patients with Anaplastic Lymphoma Kinase (*ALK*)-Rearranged Metastatic Non–Small Cell Lung Cancer (NSCLC). J. Thorac. Oncol..

[49] Solomon B.J., Liu G., Felip E., Mok T.S.K., Soo R.A., Mazieres J., Shaw A.T., de Marinis F., Goto Y., Wu Y.L. (2024). Lorlatinib Versus Crizotinib in Patients With Advanced ALK-Positive Non-Small Cell Lung Cancer: 5-Year Outcomes From the Phase III CROWN Study. J. Clin. Oncol. Off. J. Am. Soc. Clin. Oncol..

[50] Drilon A., Chiu C.-H., Fan Y., Cho B.C., Lu S., Ahn M.-J., Krebs M.G., Liu S.V., John T., Otterson G.A. (2022). Long-Term Efficacy and Safety of Entrectinib in ROS1 Fusion–Positive NSCLC. JTO Clin. Res. Rep..

[51] Drilon A., Camidge D.R., Lin J.J., Kim S.W., Solomon B.J., Dziadziuszko R., Besse B., Goto K., de Langen A.J., Wolf J. (2024). Repotrectinib in ROS1 Fusion-Positive Non-Small-Cell Lung Cancer. N. Engl. J. Med..

[52] Kilickap S., Özgüroğlu M., Sezer A., Gümüş M., Bondarenko I., Gogishvili M., Turk H.M., Cicin I., Bentsion D., Gladkov O. (2025). Cemiplimab monotherapy as first-line treatment of patients with brain metastases from advanced non-small cell lung cancer with programmed cell death-ligand 1 ≥ 50. Cancer.

[53] Mehta G.U., Vellanki P.J., Ren Y., Amatya A.K., Mishra-Kalyani P.S., Pan L., Zirkelbach J.F., Pan Y., Liu J., Aungst S.L. (2024). FDA approval summary: Fam-trastuzumab deruxtecan-nxki for unresectable or metastatic non-small cell lung cancer with activating HER2 mutations. Oncologist.

[54] Liao D., Long M., Zhang J., Wei X., Li F., Yan T., Yang D. (2024). Efficacy and safety of pralsetinib in patients with RET fusion positive non–small cell lung cancer: An observational real world study. Lung Cancer.

[55] Zhou C., Solomon B., Loong H.H., Park K., Pérol M., Arriola E., Novello S., Han B., Zhou J., Ardizzoni A. (2023). First-Line Selpercatinib or Chemotherapy and Pembrolizumab in *RET* Fusion–Positive NSCLC. N. Engl. J. Med..

[56] Griesinger F., Curigliano G., Thomas M., Subbiah V., Baik C.S., Tan D.S.W., Lee D.H., Misch D., Garralda E., Kim D.W. (2022). Safety and efficacy of pralsetinib in *RET* fusion–positive non-small-cell lung cancer including as first-line therapy: Update from the ARROW trial. Ann. Oncol..

[57] Kato T., Yang J.C., Ahn M.J., Sakai H., Morise M., Chen Y.M., Han J.Y., Yang J.J., Zhao J., Hsia T.C. (2024). Efficacy and safety of tepotinib in Asian patients with advanced NSCLC with MET exon 14 skipping enrolled in VISION. Br. J. Cancer.

[58] Dagogo-Jack I., Moonsamy P., Gainor J.F., Lennerz J.K., Piotrowska Z., Lin J.J., Lennes I.T., Sequist L.V., Shaw A.T., Goodwin K. (2021). A Phase 2 Study of Capmatinib in Patients With MET-Altered Lung Cancer Previously Treated With a MET Inhibitor. J. Thorac. Oncol. Off. Publ. Int. Assoc. Study Lung Cancer.

[59] Hochmair M.J., Vermaelen K., Mountzios G., Carcereny E., Dooms C., Lee S.-H., Morocz E., Kato T., Ciuleanu T.-E., Dy G.K. (2024). Sotorasib (960 mg or 240 mg) once daily in patients with previously treated *KRAS* G12C-mutated advanced NSCLC. Eur. J. Cancer.

[60] Yang J.C.-H., Han B., De La Mora Jiménez E., Lee J.-S., Koralewski P., Karadurmus N., Sugawara S., Livi L., Basappa N.S., Quantin X. (2024). Pembrolizumab With or Without Lenvatinib for First-Line Metastatic NSCLC With Programmed Cell Death-Ligand 1 Tumor Proportion Score of at least 1% (LEAP-007): A Randomized, Double-Blind, Phase 3 Trial. J. Thorac. Oncol..

[61] Taniguchi Y., Shimokawa T., Takiguchi Y., Misumi T., Nakamura Y., Kawashima Y., Furuya N., Shiraishi Y., Harada T., Tanaka H. (2022). A Randomized Comparison of Nivolumab versus Nivolumab + Docetaxel for Previously Treated Advanced or Recurrent ICI-Naïve Non-Small Cell Lung Cancer: TORG1630. Clin. Cancer Res. Off. J. Am. Assoc. Cancer Res..

[62] Ready N.E., Ott P.A., Hellmann M.D., Zugazagoitia J., Hann C.L., de Braud F., Antonia S.J., Ascierto P.A., Moreno V., Atmaca A. (2020). Nivolumab Monotherapy and Nivolumab Plus Ipilimumab in Recurrent Small Cell Lung Cancer: Results from the CheckMate 032 Randomized Cohort. J. Thorac. Oncol..

[63] Burotto M., Zvirbule Z., Alvarez R., Chewaskulyong B., Herraez-Baranda L.A., Shearer-Kang E., Liu X., Tosti N., Williams P., Castro Sanchez A.Y. (2024). Brief Report: Updated Data From IMscin001 Part 2, a Randomized Phase III Study of Subcutaneous Versus Intravenous Atezolizumab in Patients With Locally Advanced or Metastatic NSCLC. J. Thorac. Oncol..

[64] Borghaei H., Redman M.W., Kelly K., Waqar S.N., Robert F., Kiefer G.J., Stella P.J., Minichiello K., Gandara D.R., Herbst R.S. (2021). SWOG S1400A (NCT02154490): A Phase II Study of Durvalumab for Patients With Previously Treated Stage IV or Recurrent Squamous Cell Lung Cancer (Lung-MAP Sub-study). Clin. Lung Cancer.

[65] Ikeda M., Tanaka K. (2017). Plk1 bound to Bub1 contributes to spindle assembly checkpoint activity during mitosis. Sci. Rep..

[66] Jirawatnotai S., Dalton S., Wattanapanitch M. (2020). Role of cyclins and cyclin-dependent kinases in pluripotent stem cells and their potential as a therapeutic target. Semin. Cell Dev. Biol..

[67] Pellarin I., Dall’Acqua A., Favero A., Segatto I., Rossi V., Crestan N., Karimbayli J., Belletti B., Baldassarre G. (2025). Cyclin-dependent protein kinases and cell cycle regulation in biology and disease. Signal Transduct. Target. Ther..

[68] Gobran M., Politi A.Z., Welp L., Jakobi J., Urlaub H., Lenart P. (2025). PLK1 inhibition delays mitotic entry revealing changes to the phosphoproteome of mammalian cells early in division. EMBO J..

[69] Garrido G., Vernos I. (2016). Non-centrosomal TPX2-Dependent Regulation of the Aurora A Kinase: Functional Implications for Healthy and Pathological Cell Division. Front. Oncol..

[70] She Z.Y., Zhong N., Wei Y.L. (2022). Kinesin-5 Eg5 mediates centrosome separation to control spindle assembly in spermatocytes. Chromosoma.

[71] van Heesbeen R., Raaijmakers J.A., Tanenbaum M.E., Halim V.A., Lelieveld D., Lieftink C., Heck A.J.R., Egan D.A., Medema R.H. (2017). Aurora A, MCAK, and Kif18b promote Eg5-independent spindle formation. Chromosoma.

[72] Lee H.S., Min S., Jung Y.E., Chae S., Heo J., Lee J.H., Kim T., Kang H.C., Nakanish M., Cha S.S. (2021). Spatiotemporal coordination of the RSF1-PLK1-Aurora B cascade establishes mitotic signaling platforms. Nat. Commun..

[73] Shrestha R.L., Conti D., Tamura N., Braun D., Ramalingam R.A., Cieslinski K., Ries J., Draviam V.M. (2017). Aurora-B kinase pathway controls the lateral to end-on conversion of kinetochore-microtubule attachments in human cells. Nat. Commun..

[74] Shrestha R.L., Draviam V.M. (2013). Lateral to end-on conversion of chromosome-microtubule attachment requires kinesins CENP-E and MCAK. Curr. Biol. CB.

[75] Zhang G., Kelstrup C.D., Hu X.W., Kaas Hansen M.J., Singleton M.R., Olsen J.V., Nilsson J. (2012). The Ndc80 internal loop is required for recruitment of the Ska complex to establish end-on microtubule attachment to kinetochores. J. Cell Sci..

[76] DeLuca K.F., Lens S.M., DeLuca J.G. (2011). Temporal changes in Hec1 phosphorylation control kinetochore-microtubule attachment stability during mitosis. J. Cell Sci..

[77] Allan L.A., Camacho Reis M., Ciossani G., Huis In ‘t Veld P.J., Wohlgemuth S., Kops G.J., Musacchio A., Saurin A.T. (2020). Cyclin B1 scaffolds MAD1 at the kinetochore corona to activate the mitotic checkpoint. EMBO J..

[78] Bolanos-Garcia V.M. (2025). Mps1 kinase functions in mitotic spindle assembly and error correction. Trends Biochem. Sci..

[79] Jamasbi E., Hamelian M., Hossain M.A., Varmira K. (2022). The cell cycle, cancer development and therapy. Mol. Biol. Rep..

[80] Sarangapani K.K., Koch L.B., Nelson C.R., Asbury C.L., Biggins S. (2021). Kinetochore-bound Mps1 regulates kinetochore-microtubule attachments via Ndc80 phosphorylation. J. Cell Biol..

[81] Hayward D., Roberts E., Gruneberg U. (2022). MPS1 localizes to end-on microtubule-attached kinetochores to promote microtubule release. Curr. Biol..

[82] Zhou C.J., Wang X.Y., Dong Y.H., Wang D.H., Han Z., Zhang X.J., Sun Q.Y., Carroll J., Liang C.G. (2022). CENP-F-dependent DRP1 function regulates APC/C activity during oocyte meiosis I. Nat. Commun..

[83] Liu X., Xu L., Li J., Yao P.Y., Wang W., Ismail H., Wang H., Liao B., Yang Z., Ward T. (2020). Mitotic motor CENP-E cooperates with PRC1 in temporal control of central spindle assembly. J. Mol. Cell Biol..

[84] Adriaans I.E., Basant A., Ponsioen B., Glotzer M., Lens S.M.A. (2019). PLK1 plays dual roles in centralspindlin regulation during cytokinesis. J. Cell Biol..

[85] Babkoff A., Cohen-Kfir E., Aharon H., Ravid S. (2021). Aurora-B phosphorylates the myosin II heavy chain to promote cytokinesis. J. Biol. Chem..

[86] Kitagawa M., Fung S.Y., Onishi N., Saya H., Lee S.H. (2013). Targeting Aurora B to the equatorial cortex by MKlp2 is required for cytokinesis. PLoS ONE.

[87] Chen W., Zhu S., Zhang Y., Xiao J., Tian D. (2020). Identification of key candidate tumor biomarkers in non-small-cell lung cancer by in silico analysis. Oncol. Lett..

[88] Eggermont C., Gutierrez G.J., De Grève J., Giron P. (2023). Inhibition of PLK1 Destabilizes EGFR and Sensitizes EGFR-Mutated Lung Cancer Cells to Small Molecule Inhibitor Osimertinib. Cancers.

[89] Pan P., Liu X., Fang M., Yang S., Zhang Y., Li M., Liu Y. (2023). Silk Fibroin-Modified Liposome/Gene Editing System Knocks out the PLK1 Gene to Suppress the Growth of Lung Cancer Cells. Pharmaceutics.

[90] Van den Bossche J., Domen A., Peeters M., Deben C., De Pauw I., Jacobs J., De Bruycker S., Specenier P., Pauwels P., Vermorken J.B. (2019). Radiosensitization of Non-Small Cell Lung Cancer Cells by the Plk1 Inhibitor Volasertib Is Dependent on the p53 Status. Cancers.

[91] He Z., Ghorayeb R., Tan S., Chen K., Lorentzian A.C., Bottyan J., Aalam S.M.M., Pujana M.A., Lange P.F., Kannan N. (2022). Pathogenic BRCA1 variants disrupt PLK1-regulation of mitotic spindle orientation. Nat. Commun..

[92] Kuang P., Chen Z., Wang J., Liu Z., Wang J., Gao J., Shen L. (2017). Characterization of Aurora A and Its Impact on the Effect of Cisplatin-Based Chemotherapy in Patients with Non-Small Cell Lung Cancer. Transl. Oncol..

[93] Wang Z., Ma Z., Cao J. (2018). Effects of Repeated Aurora-A siRNA Transfection on Cilia Generation and Proliferation of SK-MES-1 or A549 Cells. Cancer Biother. Radiopharm..

[94] Schöffski P., Besse B., Gauler T., de Jonge M.J.A., Scambia G., Santoro A., Davite C., Jannuzzo M.G., Petroccione A., Delord J.P. (2015). Efficacy and safety of biweekly i.v. administrations of the Aurora kinase inhibitor danusertib hydrochloride in independent cohorts of patients with advanced or metastatic breast, ovarian, colorectal, pancreatic, small-cell and non-small-cell lung cancer: A multi-tumour, multi-institutional phase II study. Ann. Oncol..

[95] Doello S., Liang Z., Cho I.K., Kim J.B., Li Q.X. (2018). Cytotoxic Effects of 24-Methylenecyloartanyl Ferulate on A549 Nonsmall Cell Lung Cancer Cells through MYBBP1A Up-Regulation and AKT and Aurora B Kinase Inhibition. J. Agric. Food Chem..

[96] Zhou L.D., Xiong X., Long X.H., Liu Z.L., Huang S.H., Zhang W. (2014). RNA interference-mediated knockdown of Aurora-B alters the metastatic behavior of A549 cells via modulation of the phosphoinositide 3-kinase/Akt signaling pathway. Oncol. Lett..

[97] Bertran-Alamillo J., Cattan V., Schoumacher M., Codony-Servat J., Giménez-Capitán A., Cantero F., Burbridge M., Rodríguez S., Teixidó C., Roman R. (2019). AURKB as a target in non-small cell lung cancer with acquired resistance to anti-EGFR therapy. Nat. Commun..

[98] Kusakabe K., Ide N., Daigo Y., Tachibana Y., Itoh T., Yamamoto T., Hashizume H., Hato Y., Higashino K., Okano Y. (2013). Indazole-based potent and cell-active Mps1 kinase inhibitors: Rational design from pan-kinase inhibitor anthrapyrazolone (SP600125). J. Med. Chem..

[99] Tsai Y.-M., Wu K.-L., Chang Y.-Y., Hung J.-Y., Chang W.-A., Chang C.-Y., Jian S.-F., Tsai P.-H., Huang Y.-C., Chong I.-W. (2020). Upregulation of Thr/Tyr kinase Increases the Cancer Progression by Neurotensin and Dihydropyrimidinase-Like 3 in Lung Cancer. Int. J. Mol. Sci..

[100] Du L., Zhao Z., Suraokar M., Shelton S.S., Ma X., Hsiao T.-H., Minna J.D., Wistuba I., Pertsemlidis A. (2018). LMO1 functions as an oncogene by regulating TTK expression and correlates with neuroendocrine differentiation of lung cancer. Oncotarget.

[101] Chen J., Wu R., Xuan Y., Jiang M., Zeng Y. (2020). Bioinformatics analysis and experimental validation of TTK as a biomarker for prognosis in non-small cell lung cancer. Biosci. Rep..

[102] Silk A.D., Zasadil L.M., Holland A.J., Vitre B., Cleveland D.W., Weaver B.A. (2013). Chromosome missegregation rate predicts whether aneuploidy will promote or suppress tumors. Proc. Natl. Acad. Sci. USA.

[103] Weaver B.A., Silk A.D., Montagna C., Verdier-Pinard P., Cleveland D.W. (2007). Aneuploidy acts both oncogenically and as a tumor suppressor. Cancer Cell.

[104] Ma Q., Xu Y., Liao H., Cai Y., Xu L., Xiao D., Liu C., Pu W., Zhong X., Guo X. (2019). Identification and validation of key genes associated with non-small-cell lung cancer. J. Cell. Physiol..

[105] Hou S., Li N., Zhang Q., Li H., Wei X., Hao T., Li Y., Azam S., Liu C., Cheng W. (2016). XAB2 functions in mitotic cell cycle progression via transcriptional regulation of CENPE. Cell Death Dis..

[106] Shan L., Zhao M., Lu Y., Ning H., Yang S., Song Y., Chai W., Shi X. (2019). CENPE promotes lung adenocarcinoma proliferation and is directly regulated by FOXM1. Int. J. Oncol..

[107] Saijo T., Ishii G., Ochiai A., Yoh K., Goto K., Nagai K., Kato H., Nishiwaki Y., Saijo N. (2006). Eg5 expression is closely correlated with the response of advanced non-small cell lung cancer to antimitotic agents combined with platinum chemotherapy. Lung Cancer.

[108] Li Z., Yu B., Qi F., Li F. (2021). KIF11 Serves as an Independent Prognostic Factor and Therapeutic Target for Patients With Lung Adenocarcinoma. Front. Oncol..

[109] Balasundaram A., C G.P.D. (2023). In silico analysis revealed the potential circRNA-miRNA-mRNA regulative network of non-small cell lung cancer (NSCLC). Comput. Biol. Med..

[110] Jiang W., Wang P., Huang L. (2025). Upregulation of phosphatase and tensin homolog deleted on chromosome ten inhibits lung cancer cell proliferation by suppressing the oncogene polo-like kinase 1 and inducing autophagy. CytoJournal.

[111] Peng J., Zhang Q., Rao X., Allison D.B., Kong Y., Wang R., Liu J., Zhang Y., Katz W., Li Z. (2025). PLK1-mediated PDHA1 phosphorylation drives metabolic reprogramming in lung cancer. Oncogene.

[112] Zeng Y., Li N., Liu W., Zeng M., Cheng J., Huang J. (2020). Analyses of expressions and prognostic values of Polo-like kinases in non-small cell lung cancer. J. Cancer Res. Clin. Oncol..

[113] Xu R., Lee Y.J., Kim C.H., Min G.H., Kim Y.B., Park J.W., Kim D.H., Kim J.H., Yim H. (2023). Invasive FoxM1 phosphorylated by PLK1 induces the polarization of tumor-associated macrophages to promote immune escape and metastasis, amplified by IFITM1. J. Exp. Clin. Cancer Res. CR.

[114] Shin S.B., Jang H.R., Xu R., Won J.Y., Yim H. (2020). Active PLK1-driven metastasis is amplified by TGF-β signaling that forms a positive feedback loop in non-small cell lung cancer. Oncogene.

[115] Jang H.R., Shin S.B., Kim C.H., Won J.Y., Xu R., Kim D.E., Yim H. (2021). PLK1/vimentin signaling facilitates immune escape by recruiting Smad2/3 to PD-L1 promoter in metastatic lung adenocarcinoma. Cell Death Differ..

[116] Li P., Zhao Y., Lu M., Chen C., Li Y., Wang L., Zeng S., Peng Y., Liang H., Zhang G. (2025). Pharmacological inhibition of PLK1/PRC1 triggers mitotic catastrophe and sensitizes lung cancers to chemotherapy. Cell Death Dis..

[117] Li Z., Li J., Bi P., Lu Y., Burcham G., Elzey B.D., Ratliff T., Konieczny S.F., Ahmad N., Kuang S. (2014). Plk1 phosphorylation of PTEN causes a tumor-promoting metabolic state. Mol. Cell. Biol..

[118] Zhang Q., Peng J., Li Z., Rao X., Allison D.B., Qiao Q., Zhang Z., Kong Y., Zhang Y., Wang R. (2025). PLK1-mediated PDHA1 phosphorylation drives mitochondrial dysfunction, mitophagy, and cancer progression in Cr(VI)-associated lung cancer. J. Biol. Chem..

[119] Kong Y., Li C., Liu J., Wu S., Zhang M., Allison D.B., Hassan F., He D., Wang X., Mao F. (2024). Single-cell analysis identifies PLK1 as a driver of immunosuppressive tumor microenvironment in LUAD. PLoS Genet..

[120] Wang W., Zhao R., Wang Y., Pan L., Luan F., Fu G. (2025). PLK1 in cancer therapy: A comprehensive review of immunomodulatory mechanisms and therapeutic opportunities. Front. Immunol..

[121] Li C., Allison D.B., He D., Mao F., Wang X., Rychahou P., Imam I.A., Kong Y., Zhang Q., Zhang Y. (2023). Phosphorylation of AHR by PLK1 promotes metastasis of LUAD via DIO2-TH signaling. PLoS Genet..

[122] Chen M., Zhang S., Wang F., He J., Jiang W., Zhang L. (2024). DLGAP5 promotes lung adenocarcinoma growth via upregulating PLK1 and serves as a therapeutic target. J. Transl. Med..

[123] Miralaei N., Majd A., Ghaedi K., Peymani M., Safaei M. (2021). Integrated pan-cancer of AURKA expression and drug sensitivity analysis reveals increased expression of AURKA is responsible for drug resistance. Cancer Med..

[124] Zheng X., Chi J., Zhi J., Zhang H., Yue D., Zhao J., Li D., Li Y., Gao M., Guo J. (2018). Aurora-A-mediated phosphorylation of LKB1 compromises LKB1/AMPK signaling axis to facilitate NSCLC growth and migration. Oncogene.

[125] Xu H.T., Ma L., Qi F.J., Liu Y., Yu J.H., Dai S.D., Zhu J.J., Wang E.H. (2006). Expression of serine threonine kinase 15 is associated with poor differentiation in lung squamous cell carcinoma and adenocarcinoma. Pathol. Int..

[126] Dong S., Men W., Yang S., Xu S. (2020). Identification of lung adenocarcinoma biomarkers based on bioinformatic analysis and human samples. Oncol. Rep..

[127] Takeshita M., Koga T., Takayama K., Kouso H., Nishimura-Ikeda Y., Yoshino I., Maehara Y., Nakanishi Y., Sueishi K. (2008). CHFR expression is preferentially impaired in smoking-related squamous cell carcinoma of the lung, and the diminished expression significantly harms outcomes. Int. J. Cancer.

[128] Lin X., Xiang X., Hao L., Wang T., Lai Y., Abudoureyimu M., Zhou H., Feng B., Chu X., Wang R. (2020). The role of Aurora-A in human cancers and future therapeutics. Am. J. Cancer Res..

[129] Wang P., Gong Y., Guo T., Li M., Fang L., Yin S., Kamran M., Liu Y., Xu J., Xu L. (2019). Activation of Aurora A kinase increases YAP stability via blockage of autophagy. Cell Death Dis..

[130] Li M., Sun C., Bu X., Que Y., Zhang L., Zhang Y., Zhang L., Lu S., Huang J., Zhu J. (2021). ISL1 promoted tumorigenesis and EMT via Aurora kinase A-induced activation of PI3K/AKT signaling pathway in neuroblastoma. Cell Death Dis..

[131] Zhong Y., Yang J., Xu W.W., Wang Y., Zheng C.C., Li B., He Q.Y. (2017). KCTD12 promotes tumorigenesis by facilitating CDC25B/CDK1/Aurora A-dependent G2/M transition. Oncogene.

[132] Dos Santos E.O., Carneiro-Lobo T.C., Aoki M.N., Levantini E., Bassères D.S. (2016). Aurora kinase targeting in lung cancer reduces KRAS-induced transformation. Mol. Cancer.

[133] Yu J., Zhou J., Xu F., Bai W., Zhang W. (2018). High expression of Aurora-B is correlated with poor prognosis and drug resistance in non-small cell lung cancer. Int. J. Biol. Markers.

[134] Li X., Li H., Li S., Zhu F., Kim D.J., Xie H., Li Y., Nadas J., Oi N., Zykova T.A. (2012). Ceftriaxone, an FDA-approved cephalosporin antibiotic, suppresses lung cancer growth by targeting Aurora B. Carcinogenesis.

[135] Oser M.G., Fonseca R., Chakraborty A.A., Brough R., Spektor A., Jennings R.B., Flaifel A., Novak J.S., Gulati A., Buss E. (2019). Cells Lacking the RB1 Tumor Suppressor Gene Are Hyperdependent on Aurora B Kinase for Survival. Cancer Discov..

[136] Hayama S., Daigo Y., Yamabuki T., Hirata D., Kato T., Miyamoto M., Ito T., Tsuchiya E., Kondo S., Nakamura Y. (2007). Phosphorylation and activation of cell division cycle associated 8 by aurora kinase B plays a significant role in human lung carcinogenesis. Cancer Res..

[137] Takeshita M., Koga T., Takayama K., Ijichi K., Yano T., Maehara Y., Nakanishi Y., Sueishi K. (2013). Aurora-B overexpression is correlated with aneuploidy and poor prognosis in non-small cell lung cancer. Lung Cancer.

[138] Chen X., Yu C., Gao J., Zhu H., Cui B., Zhang T., Zhou Y., Liu Q., He H., Xiao R. (2018). A novel USP9X substrate TTK contributes to tumorigenesis in non-small-cell lung cancer. Theranostics.

[139] Zheng L., Chen Z., Kawakami M., Chen Y., Roszik J., Mustachio L.M., Kurie J.M., Villalobos P., Lu W., Behrens C. (2019). Tyrosine Threonine Kinase Inhibition Eliminates Lung Cancers by Augmenting Apoptosis and Polyploidy. Mol. Cancer Ther..

[140] Shi Y.-X., Dai P.-H., Jiang Y.-F., Wang Y.-Q., Liu W. (2023). A pan-cancer landscape of centromere proteins in tumorigenesis and anticancer drug sensitivity. Transl. Oncol..

[141] Pei N., Cao L., Liu Y., Wu J., Song Q., Zhang Z., Yuan J., Zhang X. (2015). XAB2 tagSNPs contribute to non-small cell lung cancer susceptibility in Chinese population. BMC Cancer.

[142] Maiti P., Sharma P., Nand M., Bhatt I.D., Ramakrishnan M.A., Mathpal S., Joshi T., Pant R., Mahmud S., Simal-Gandara J. (2022). Integrated Machine Learning and Chemoinformatics-Based Screening of Mycotic Compounds against Kinesin Spindle ProteinEg5 for Lung Cancer Therapy. Molecules.

[143] Liu J., Feng Y., Zeng X., He M., Gong Y., Liu Y. (2021). LncRNA VPS9D1-AS1 Promotes Malignant Progression of Lung Adenocarcinoma by Targeting miRNA-30a-5p/KIF11 Axis. Front. Genet..

[144] Mengyan X., Kun D., Xinming J., Yutian W., Yongqian S. (2022). Identification and verification of hub genes associated with the progression of non-small cell lung cancer by integrated analysis. Front. Pharmacol..

[145] Ling J., Wang Y., Ma L., Zheng Y., Tang H., Meng L., Zhang L. (2022). KIF11, a plus end-directed kinesin, as a key gene in benzo(a)pyrene-induced non-small cell lung cancer. Environ. Toxicol. Pharmacol..

[146] Sebastian M., Reck M., Waller C.F., Kortsik C., Frickhofen N., Schuler M., Fritsch H., Gaschler-Markefski B., Hanft G., Munzert G. (2010). The Efficacy and Safety of BI 2536, a Novel Plk-1 Inhibitor, in Patients with Stage IIIB/IV Non-small Cell Lung Cancer Who Had Relapsed after, or Failed, Chemotherapy: Results from an Open-Label, Randomized Phase II Clinical Trial. J. Thorac. Oncol..

[147] Awad M.M., Chu Q.S., Gandhi L., Stephenson J.J., Govindan R., Bradford D.S., Bonomi P.D., Ellison D.M., Eaton K.D., Fritsch H. (2017). An open-label, phase II study of the polo-like kinase-1 (Plk-1) inhibitor, BI 2536, in patients with relapsed small cell lung cancer (SCLC). Lung Cancer.

[148] Melichar B., Adenis A., Lockhart A.C., Bennouna J., Dees E.C., Kayaleh O., Obermannova R., DeMichele A., Zatloukal P., Zhang B. (2015). Safety and activity of alisertib, an investigational aurora kinase A inhibitor, in patients with breast cancer, small-cell lung cancer, non-small-cell lung cancer, head and neck squamous-cell carcinoma, and gastro-oesophageal adenocarcinoma: A five-arm phase 2 study. Lancet Oncol..

[149] Robbrecht D.G.J., Lopez J., Calvo E., He X., Hiroshi H., Soni N., Cook N., Dowlati A., Fasolo A., Moreno V. (2021). A first-in-human phase 1 and pharmacological study of TAS-119, a novel selective Aurora A kinase inhibitor in patients with advanced solid tumours. Br. J. Cancer.

[150] Chung V., Heath E.I., Schelman W.R., Johnson B.M., Kirby L.C., Lynch K.M., Botbyl J.D., Lampkin T.A., Holen K.D. (2012). First-time-in-human study of GSK923295, a novel antimitotic inhibitor of centromere-associated protein E (CENP-E), in patients with refractory cancer. Cancer Chemother. Pharmacol..

[151] Schöffski P., Awada A., de la Bigne A.M., Felloussi Z., Burbridge M., Cantero F., Colombo R., Maruzzelli S., Ammattatelli K., de Jonge M. (2022). First-in-man, first-in-class phase I study with the monopolar spindle 1 kinase inhibitor S81694 administered intravenously in adult patients with advanced, metastatic solid tumours. Eur. J. Cancer.

[152] Wakui H., Yamamoto N., Kitazono S., Mizugaki H., Nakamichi S., Fujiwara Y., Nokihara H., Yamada Y., Suzuki K., Kanda H. (2014). A phase 1 and dose-finding study of LY2523355 (litronesib), an Eg5 inhibitor, in Japanese patients with advanced solid tumors. Cancer Chemother. Pharmacol..

[153] Partscht P., Simon A., Chen N.P., Erhardt S., Schiebel E. (2023). The HIPK2/CDC14B-MeCP2 axis enhances the spindle assembly checkpoint block by promoting cyclin B translation. Sci. Adv..

[154] Serrano-del Valle A., Reina-Ortiz C., Benedi A., Anel A., Naval J., Marzo I. (2021). Future prospects for mitosis-targeted antitumor therapies. Biochem. Pharmacol..

[155] Pinto B., Novais P., Henriques A.C., Carvalho-Tavares J., Silva P.M.A., Bousbaa H. (2022). Navitoclax Enhances the Therapeutic Effects of PLK1 Targeting on Lung Cancer Cells in 2D and 3D Culture Systems. Pharmaceutics.

[156] Chinn D.C., Holland W.S., Mack P.C. (2014). Anticancer activity of the Aurora A kinase inhibitor MK-5108 in non-small-cell lung cancer (NSCLC) in vitro as monotherapy and in combination with chemotherapies. J. Cancer Res. Clin. Oncol..

[157] Sinha D., Duijf P.H.G., Khanna K.K. (2019). Mitotic slippage: An old tale with a new twist. Cell Cycle.

[158] Ghelli Luserna di Rorà A., Martinelli G., Simonetti G. (2019). The balance between mitotic death and mitotic slippage in acute leukemia: A new therapeutic window?. J. Hematol. Oncol..

[159] Lim B., Greer Y., Lipkowitz S., Takebe N. (2019). Novel Apoptosis-Inducing Agents for the Treatment of Cancer, a New Arsenal in the Toolbox. Cancers.

[160] Tsuji K., Kikuchi E., Takashima Y., Shoji T., Takahashi H., Ito S., Morinaga D., Kashima M., Maeda M., Kitai H. (2023). Inhibition of non-homologous end joining mitigates paclitaxel resistance resulting from mitotic slippage in non-small cell lung cancer. Cell Cycle.

[161] Koch A., Maia A., Janssen A., Medema R.H. (2016). Molecular basis underlying resistance to Mps1/TTK inhibitors. Oncogene.

[162] Han Y., Wu Y., Xu Y., Guo W., Zhang N., Wang X. (2019). Molecular mechanism of point mutation-induced Monopolar spindle 1 (Mps1/TTK) inhibitor resistance revealed by a comprehensive molecular modeling study. PeerJ.

[163] Hiruma Y., Koch A., Hazraty N., Tsakou F., Medema R.H., Joosten R.P., Perrakis A. (2017). Understanding inhibitor resistance in Mps1 kinase through novel biophysical assays and structures. J. Biol. Chem..

[164] Pisa R., Phua D.Y.Z., Kapoor T.M. (2020). Distinct Mechanisms of Resistance to a CENP-E Inhibitor Emerge in Near-Haploid and Diploid Cancer Cells. Cell Chem. Biol..

[165] Niu H., Shin H., Gao F., Zhang J., Bahamon B., Danaee H., Melichar B., Schilder R.J., Coleman R.L., Falchook G. (2017). Aurora A Functional Single Nucleotide Polymorphism (SNP) Correlates With Clinical Outcome in Patients With Advanced Solid Tumors Treated With Alisertib, an Investigational Aurora A Kinase Inhibitor. EBioMedicine.

[166] Yücer R., Piccinno R., Ooko E., Dawood M., Bringmann G., Efferth T. (2025). Predictive and Prognostic Relevance of ABC Transporters for Resistance to Anthracycline Derivatives. Biomolecules.

[167] Wu Z.X., Yang Y., Wang G., Wang J.Q., Teng Q.X., Sun L., Lei Z.N., Lin L., Chen Z.S., Zou C. (2020). Dual TTK/CLK2 inhibitor, CC-671, selectively antagonizes ABCG2-mediated multidrug resistance in lung cancer cells. Cancer Sci..

[168] Shin S.B., Kim D.H., Kim D.E., Aldonza M.B.D., Kim Y., Yim H. (2021). Dual Targeting of EGFR with PLK1 Exerts Therapeutic Synergism in Taxane-Resistant Lung Adenocarcinoma by Suppressing ABC Transporters. Cancers.

[169] Wu C.P., Hsiao S.H., Sim H.M., Luo S.Y., Tuo W.C., Cheng H.W., Li Y.Q., Huang Y.H., Ambudkar S.V. (2013). Human ABCB1 (P-glycoprotein) and ABCG2 mediate resistance to BI 2536, a potent and selective inhibitor of Polo-like kinase 1. Biochem. Pharmacol..

[170] Ansbro M.R., Shukla S., Ambudkar S.V., Yuspa S.H., Li L. (2013). Screening compounds with a novel high-throughput ABCB1-mediated efflux assay identifies drugs with known therapeutic targets at risk for multidrug resistance interference. PLoS ONE.

[171] Marchetti S., Pluim D., van Eijndhoven M., van Tellingen O., Mazzanti R., Beijnen J.H., Schellens J.H. (2013). Effect of the drug transporters ABCG2, Abcg2, ABCB1 and ABCC2 on the disposition, brain accumulation and myelotoxicity of the aurora kinase B inhibitor barasertib and its more active form barasertib-hydroxy-QPA. Investig. New Drugs.

[172] Lee T.D., Lee O.W., Brimacombe K.R., Chen L., Guha R., Lusvarghi S., Tebase B.G., Klumpp-Thomas C., Robey R.W., Ambudkar S.V. (2019). A High-Throughput Screen of a Library of Therapeutics Identifies Cytotoxic Substrates of P-glycoprotein. Mol. Pharmacol..

[173] Yan T., Shi J. (2024). Angiogenesis and EMT regulators in the tumor microenvironment in lung cancer and immunotherapy. Front. Immunol..

[174] De Las Rivas J., Brozovic A., Izraely S., Casas-Pais A., Witz I.P., Figueroa A. (2021). Cancer drug resistance induced by EMT: Novel therapeutic strategies. Arch. Toxicol..

[175] Ferrarotto R., Goonatilake R., Yoo S.Y., Tong P., Giri U., Peng S., Minna J., Girard L., Wang Y., Wang L. (2016). Epithelial-Mesenchymal Transition Predicts Polo-Like Kinase 1 Inhibitor-Mediated Apoptosis in Non-Small Cell Lung Cancer. Clin. Cancer Res. Off. J. Am. Assoc. Cancer Res..

[176] Lin X., Kang K., Chen P., Zeng Z., Li G., Xiong W., Yi M., Xiang B. (2024). Regulatory mechanisms of PD-1/PD-L1 in cancers. Mol. Cancer.

[177] Reda M., Ngamcherdtrakul W., Nelson M.A., Siriwon N., Wang R., Zaidan H.Y., Bejan D.S., Reda S., Hoang N.H., Crumrine N.A. (2022). Development of a nanoparticle-based immunotherapy targeting PD-L1 and PLK1 for lung cancer treatment. Nat. Commun..

[178] Ellis P.M., Leighl N.B., Hirsh V., Reaume M.N., Blais N., Wierzbicki R., Sadrolhefazi B., Gu Y., Liu D., Pilz K. (2015). A Randomized, Open-Label Phase II Trial of Volasertib as Monotherapy and in Combination With Standard-Dose Pemetrexed Compared With Pemetrexed Monotherapy in Second-Line Treatment for Non-Small-Cell Lung Cancer. Clin. Lung Cancer.

[179] Park S., Shim J., Mortimer P.G.S., Smith S.A., Godin R.E., Hollingsworth S.J., Kim H.J., Jung H.A., Sun J.M., Park W.Y. (2020). Biomarker-driven phase 2 umbrella trial study for patients with recurrent small cell lung cancer failing platinum-based chemotherapy. Cancer.

[180] Yao D., Gu P., Wang Y., Luo W., Chi H., Ge J., Qian Y. (2018). Inhibiting polo-like kinase 1 enhances radiosensitization via modulating DNA repair proteins in non-small-cell lung cancer. Biochem. Cell Biol..

[181] Rastogi S., Joshi A., Sato N., Lee S., Lee M.J., Trepel J.B., Neckers L. (2024). An update on the status of HSP90 inhibitors in cancer clinical trials. Cell Stress Chaperones.

[182] Zhou C., Yu T., Zhu R., Lu J., Ouyang X., Zhang Z., Chen Q., Li J., Cui J., Jiang F. (2023). Timosaponin AIII promotes non-small-cell lung cancer ferroptosis through targeting and facilitating HSP90 mediated GPX4 ubiquitination and degradation. Int. J. Biol. Sci..

[183] O’Connell B.C., O’Callaghan K., Tillotson B., Douglas M., Hafeez N., West K.A., Stern H., Ali J.A., Changelian P., Fritz C.C. (2014). HSP90 inhibition enhances antimitotic drug-induced mitotic arrest and cell death in preclinical models of non-small cell lung cancer. PLoS ONE.

[184] Zhou Q.Y., Gui S.Y., Zhang P., Wang M. (2021). Upregulation of miR-345-5p suppresses cell growth of lung adenocarcinoma by regulating ras homolog family member A (RhoA) and Rho/Rho associated protein kinase (Rho/ROCK) pathway. Chin. Med. J..

[185] Wang J., Hu K., Guo J., Cheng F., Lv J., Jiang W., Lu W., Liu J., Pang X., Liu M. (2016). Suppression of KRas-mutant cancer through the combined inhibition of KRAS with PLK1 and ROCK. Nat. Commun..

[186] Zhang J., Liu X., Hou P., Lv Y., Li G., Cao G., Wang H., Lin W. (2024). BRCA1 orchestrates the response to BI-2536 and its combination with alisertib in MYC-driven small cell lung cancer. Cell Death Dis..

[187] Wang Y., Singh R., Wang L., Nilsson M., Goonatilake R., Tong P., Li L., Giri U., Villalobos P., Mino B. (2016). Polo-like kinase 1 inhibition diminishes acquired resistance to epidermal growth factor receptor inhibition in non-small cell lung cancer with T790M mutations. Oncotarget.

[188] Li J., Zhang D., Wang S., Yu P., Sun J., Zhang Y., Meng X., Li J., Xiang L. (2025). Baicalein induces apoptosis by inhibiting the glutamine-mTOR metabolic pathway in lung cancer. J. Adv. Res..

[189] Montaudon E., El Botty R., Vacher S., Déas O., Naguez A., Chateau-Joubert S., Treguer D., de Plater L., Zemoura L., Némati F. (2021). High in vitro and in vivo synergistic activity between mTORC1 and PLK1 inhibition in adenocarcinoma NSCLC. Oncotarget.

[190] Zhao S., Li Y., Li G., Ye J., Wang R., Zhang X., Li F., Gao C., Li J., Jiang J. (2023). PI3K/mTOR inhibitor VS-5584 combined with PLK1 inhibitor exhibits synergistic anti-cancer effects on non-small cell lung cancer. Eur. J. Pharmacol..

[191] Pinto B., Pacheco C., Silva P., Carvalho-Tavares J., Sarmento B., Bousbaa H. (2022). Nanomedicine internalization and penetration: Why should we use spheroids?. Sci. Lett..

[192] Reda M., Ngamcherdtrakul W., Gu S., Bejan D.S., Siriwon N., Gray J.W., Yantasee W. (2019). PLK1 and EGFR targeted nanoparticle as a radiation sensitizer for non-small cell lung cancer. Cancer Lett..

[193] Ellis P.M., Chu Q.S., Leighl N., Laurie S.A., Fritsch H., Gaschler-Markefski B., Gyorffy S., Munzert G. (2013). A phase I open-label dose-escalation study of intravenous BI 2536 together with pemetrexed in previously treated patients with non-small-cell lung cancer. Clin. Lung Cancer.

[194] Sootome H., Miura A., Masuko N., Suzuki T., Uto Y., Hirai H. (2020). Aurora A Inhibitor TAS-119 Enhances Antitumor Efficacy of Taxanes In Vitro and In Vivo: Preclinical Studies as Guidance for Clinical Development and Trial Design. Mol. Cancer Ther..

[195] Lim K.H., Opyrchal M., Acharya A., Boice N., Wu N., Gao F., Webster J., Lockhart A.C., Waqar S.N., Govindan R. (2021). Phase 1 study combining alisertib with nab-paclitaxel in patients with advanced solid malignancies. Eur. J. Cancer.

[196] Owonikoko T.K., Niu H., Nackaerts K., Csoszi T., Ostoros G., Mark Z., Baik C., Joy A.A., Chouaid C., Jaime J.C. (2020). Randomized Phase II Study of Paclitaxel plus Alisertib versus Paclitaxel plus Placebo as Second-Line Therapy for SCLC: Primary and Correlative Biomarker Analyses. J. Thorac. Oncol. Off. Publ. Int. Assoc. Study Lung Cancer.

[197] Liu N., Wang Y.A., Sun Y., Ecsedy J., Sun J., Li X., Wang P. (2019). Inhibition of Aurora A enhances radiosensitivity in selected lung cancer cell lines. Respir. Res..

[198] Roberts S.K., Galgadas I., Clarke D.T., Zanetti-Domingues L.C., Gervasio F.L., Martin-Fernandez M.L. (2025). Targeting mutant EGFR in non-small cell lung cancer in the context of cell adaptation and resistance. Drug Discov. Today.

[199] Bagnyukova T., Egleston B.L., Pavlov V.A., Serebriiskii I.G., Golemis E.A., Borghaei H. (2024). Synergy of EGFR and AURKA Inhibitors in KRAS-mutated Non-small Cell Lung Cancers. Cancer Res. Commun..

[200] Shah K.N., Bhatt R., Rotow J., Rohrberg J., Olivas V., Wang V.E., Hemmati G., Martins M.M., Maynard A., Kuhn J. (2019). Aurora kinase A drives the evolution of resistance to third-generation EGFR inhibitors in lung cancer. Nat. Med..

[201] Li Y., Mahadevan N.R., Duplaquet L., Hong D., Durmaz Y.T., Jones K.L., Cho H., Morrow M., Protti A., Poitras M.J. (2023). Aurora A kinase inhibition induces accumulation of SCLC tumor cells in mitosis with restored interferon signaling to increase response to PD-L1. Cell Rep. Med..

[202] Dhanasekaran R., Deutzmann A., Mahauad-Fernandez W.D., Hansen A.S., Gouw A.M., Felsher D.W. (2022). The MYC oncogene—The grand orchestrator of cancer growth and immune evasion. Nat. Rev. Clin. Oncol..

[203] Kolla B.C., Racila E., Patel M.R. (2020). Deep and Prolonged Response to Aurora A Kinase Inhibitor and Subsequently to Nivolumab in MYCL1-Driven Small-Cell Lung Cancer: Case Report and Literature Review. Case Rep. Oncol. Med..

[204] Chang C.P., Yeh T.K., Chen C.T., Wang W.P., Chen Y.T., Tsai C.H., Chen Y.F., Ke Y.Y., Wang J.Y., Chen C.P. (2024). Discovery of a Long Half-Life AURKA Inhibitor to Treat MYC-Amplified Solid Tumors as a Monotherapy and in Combination with Everolimus. Mol. Cancer Ther..

[205] Semrad T.J., Kim E.J., Gong I.Y., Li T., Christensen S., Arora M., Riess J.W., Gandara D.R., Kelly K. (2021). Phase 1 study of alisertib (MLN8237) and weekly irinotecan in adults with advanced solid tumors. Cancer Chemother. Pharmacol..

[206] Roper N., Brown A.L., Wei J.S., Pack S., Trindade C., Kim C., Restifo O., Gao S., Sindiri S., Mehrabadi F. (2020). Clonal Evolution and Heterogeneity of Osimertinib Acquired Resistance Mechanisms in EGFR Mutant Lung Cancer. Cell Rep. Med..

[207] Tanaka K., Yu H.A., Yang S., Han S., Selcuklu S.D., Kim K., Ramani S., Ganesan Y.T., Moyer A., Sinha S. (2021). Targeting Aurora B kinase prevents and overcomes resistance to EGFR inhibitors in lung cancer by enhancing BIM- and PUMA-mediated apoptosis. Cancer Cell.

[208] Iwasa T., Okamoto I., Suzuki M., Hatashita E., Yamada Y., Fukuoka M., Ono K., Nakagawa K. (2009). Inhibition of insulin-like growth factor 1 receptor by CP-751,871 radiosensitizes non-small cell lung cancer cells. Clin. Cancer Res. Off. J. Am. Assoc. Cancer Res..

[209] Hua H., Kong Q., Yin J., Zhang J., Jiang Y. (2020). Insulin-like growth factor receptor signaling in tumorigenesis and drug resistance: A challenge for cancer therapy. J. Hematol. Oncol..

[210] Ikeda Y., Yasutake R., Yuki R., Saito Y., Nakayama Y. (2021). Combination Treatment of OSI-906 with Aurora B Inhibitor Reduces Cell Viability via Cyclin B1 Degradation-Induced Mitotic Slippage. Int. J. Mol. Sci..

[211] Sak A., Stuschke M., Groneberg M., Kübler D., Pöttgen C., Eberhardt W.E. (2012). Inhibiting the aurora B kinase potently suppresses repopulation during fractionated irradiation of human lung cancer cell lines. Int. J. Radiat. Oncol. Biol. Phys..

[212] Fan S., Chen Y., Wang W., Xu W., Tian M., Liu Y., Zhou Y., Liu D., Xia Q., Dong L. (2024). Pharmacological and Biological Targeting of FGFR1 in Cancer. Curr. Issues Mol. Biol..

[213] Spagnolo C.C., Ciappina G., Giovannetti E., Squeri A., Granata B., Lazzari C., Pretelli G., Pasello G., Santarpia M. (2023). Targeting MET in Non-Small Cell Lung Cancer (NSCLC): A New Old Story?. Int. J. Mol. Sci..

[214] Clémenson C., Chargari C., Liu W., Mondini M., Ferté C., Burbridge M.F., Cattan V., Jacquet-Bescond A., Deutsch E. (2017). The MET/AXL/FGFR Inhibitor S49076 Impairs Aurora B Activity and Improves the Antitumor Efficacy of Radiotherapy. Mol. Cancer Ther..

[215] Sumi N.J., Ctortecka C., Hu Q., Bryant A.T., Fang B., Remsing Rix L.L., Ayaz M., Kinose F., Welsh E.A., Eschrich S.A. (2019). Divergent Polypharmacology-Driven Cellular Activity of Structurally Similar Multi-Kinase Inhibitors through Cumulative Effects on Individual Targets. Cell Chem. Biol..

[216] Liu Y., Wang C., Su H., Birchler J.A., Han F. (2021). Phosphorylation of histone H3 by Haspin regulates chromosome alignment and segregation during mitosis in maize. J. Exp. Bot..

[217] Huang M., Feng X., Su D., Wang G., Wang C., Tang M., Paulucci-Holthauzen A., Hart T., Chen J. (2020). Genome-wide CRISPR screen uncovers a synergistic effect of combining Haspin and Aurora kinase B inhibition. Oncogene.

[218] Ramkumar K., Tanimoto A., Della Corte C.M., Stewart C.A., Wang Q., Shen L., Cardnell R.J., Wang J., Polanska U.M., Andersen C. (2023). Targeting BCL2 Overcomes Resistance and Augments Response to Aurora Kinase B Inhibition by AZD2811 in Small Cell Lung Cancer. Clin. Cancer Res. Off. J. Am. Assoc. Cancer Res..

[219] Xiao H., Zheng Y., Ma L., Tian L., Sun Q. (2021). Clinically-Relevant ABC Transporter for Anti-Cancer Drug Resistance. Front. Pharmacol..

[220] Kitajima S., Ivanova E., Guo S., Yoshida R., Campisi M., Sundararaman S.K., Tange S., Mitsuishi Y., Thai T.C., Masuda S. (2019). Suppression of STING Associated with LKB1 Loss in KRAS-Driven Lung Cancer. Cancer Discov..

[221] Kitajima S., Tani T., Springer B.F., Campisi M., Osaki T., Haratani K., Chen M., Knelson E.H., Mahadevan N.R., Ritter J. (2022). MPS1 inhibition primes immunogenicity of KRAS-LKB1 mutant lung cancer. Cancer Cell.

[222] Atrafi F., Boix O., Subbiah V., Diamond J.R., Chawla S.P., Tolcher A.W., LoRusso P.M., Eder J.P., Gutierrez M., Sankhala K. (2021). A Phase I Study of an MPS1 Inhibitor (BAY 1217389) in Combination with Paclitaxel Using a Novel Randomized Continual Reassessment Method for Dose Escalation. Clin. Cancer Res. Off. J. Am. Assoc. Cancer Res..

[223] Sakuma Y., Hirai S., Sumi T., Niki T., Yamaguchi M. (2023). Dual inhibition of KIF11 and BCL2L1 induces apoptosis in lung adenocarcinoma cells. Biochem. Biophys. Res. Commun..

[224] Sakuma Y., Hirai S., Yamaguchi M., Idogawa M. (2024). Small Cell Lung Carcinoma Cells Depend on KIF11 for Survival. Int. J. Mol. Sci..

[225] Wang F., Fu X., Chang M., Wei T., Lin R., Tong H., Zhang X., Yuan R., Zhou Z., Huang X. (2024). The Interaction of Calcium-Sensing Receptor with KIF11 Enhances Cisplatin Resistance in Lung Adenocarcinoma via BRCA1/cyclin B1 pathway. Int. J. Biol. Sci..

[226] Lee M.S., Johansen L., Zhang Y., Wilson A., Keegan M., Avery W., Elliott P., Borisy A.A., Keith C.T. (2007). The novel combination of chlorpromazine and pentamidine exerts synergistic antiproliferative effects through dual mitotic action. Cancer Res..

[227] Henriques A.C., Ribeiro D., Pedrosa J., Sarmento B., Silva P.M.A., Bousbaa H. (2019). Mitosis inhibitors in anticancer therapy: When blocking the exit becomes a solution. Cancer Lett..

[228] Sati P., Sharma E., Dhyani P., Attri D.C., Rana R., Kiyekbayeva L., Büsselberg D., Samuel S.M., Sharifi-Rad J. (2024). Paclitaxel and its semi-synthetic derivatives: Comprehensive insights into chemical structure, mechanisms of action, and anticancer properties. Eur. J. Med. Res..

[229] Weaver B.A. (2014). How Taxol/paclitaxel kills cancer cells. Mol. Biol. Cell.

[230] Kemp J.A., Kwon Y.J. (2021). Cancer nanotechnology: Current status and perspectives. Nano Converg..

